# Thermophysical and Electrochemical Properties of Ethereal Functionalised Cyclic Alkylammonium‐based Ionic Liquids as Potential Electrolytes for Electrochemical Applications

**DOI:** 10.1002/cphc.201700246

**Published:** 2017-06-21

**Authors:** Alex R. Neale, Sinead Murphy, Peter Goodrich, Christopher Hardacre, Johan Jacquemin

**Affiliations:** ^1^ School of Chemistry and Chemical Engineering Queen's University Belfast David Keir Building, Stranmillis Road Belfast BT9 5AG UK; ^2^ School of Chemical Engineering & Analytical Science The University of Manchester The Mill, Sackville Street Manchester M13 9PL UK; ^3^ Laboratoire PCM2E Université François Rabelais Parc de Grandmont 37200 Tours France

**Keywords:** ammonium cations, electrochemistry, electrolytes, ether functionalisation, ionic liquids

## Abstract

A series of hydrophobic room temperature ionic liquids (ILs) based on ethereal functionalised pyrrolidinium, piperidinium and azepanium cations bearing the bis[(trifluoromethyl)sulfonyl]imide, [TFSI]^−^, anion were synthesized and characterized. Their physicochemical properties such as density, viscosity and electrolytic conductivity, and thermal properties including phase transition behaviour and decomposition temperature have been measured. All of the ILs showed low melting point, low viscosity and good conductivity and the latter properties have been discussed in terms of the IL fragility, an important electrolyte feature of the transport properties of glass‐forming ILs. Furthermore, the studied [TFSI]^−^‐based ILs generally exhibit good electrochemical stabilities and, by coupling electrochemical experiments and DFT calculations, the effect of ether functionalisation at the IL cation on the electrochemical stability of the IL is discussed. Preliminary investigations into the Li‐redox chemistry at a Cu working electrode are also reported as a function of ether‐functionality within the pyrrolidinium‐based IL family. Overall, the results show that these ionic liquids are suitable for electrochemical devices such as battery systems, fuel cells or supercapacitors.

## 1 Introduction

Ionic liquids have gained considerable interest over the past few decades due to their numerous attractive properties such as extremely low vapour pressure, low flammability, high thermal stability and large liquid range. This has led to a number of groups researching ionic liquid applications particularly in the fields of catalysis^1^, ^2^, ^3^, separation ^[4, 5]^ and nanotechnology.^6^ In comparison with molecular solvents, ionic liquids also possess good ionic conductivity coupled with good electrochemical stability and, therefore, have been proposed as new electrolytes for energy devices^7^, ^8^, ^9^, ^10^, ^11^, ^12^, ^13^, ^14^, ^15^, ^16^, ^17^, ^18^ and as solvents for electrodeposition of metals.^19^, ^20^

Within the field of energy storage devices, a number of ILs have been developed with specific physiochemical applications. To date, however, most of these are based on imidazolium,^21^ quaternary ammonium,^22^, ^23^ pyridinium^24^ and quaternary phosphonium cations.^25^, ^26^ Therein, the phosphonium, aliphatic and cyclic ammonium cations show a higher resistance to electrochemical reduction than corresponding imidazolium and pyridinium analogues and are, therefore, considered to be more promising as electrolytes. Within the cyclic aliphatic ammonium cations, typically pyrrolidinium,^27^, ^28^, ^29^ piperidinium^28^, ^30^ and more recently azepanium^31^, ^32^ cationic based structures have been chosen for a large number of investigations for potential electrochemical devices. However, the downside to this improved stability is an increase in viscosity which results in significant drops in performance at mid‐to‐high current rates.^12^, ^13^

Attempts to reduce the viscosity of ILs based on cyclic alkylammonium‐[TFSI] based ILs have been achieved by blending with appropriate organic solvents,^33^, ^34^, ^35^ utilising alternative anion structures,^14^, ^36^, ^37^ and functionalisation of the alkyl chains of the cyclic aliphatic ammonium cations with different group functionalities. In this regard, some studies have highlighted the positive influence of ethereal functionalisation on the transport properties of acyclic and cyclic ammonium‐based ionic liquids.^37^, ^38^, ^39^, ^40^, ^41^ In contrast, ionic liquids containing two or more ether groups appended to the cation have been somewhat understudied. Functionalised quaternary ammonium ILs based on di‐, tri‐ and tetraethereal‐based cations have recently been reported as promising candidates for energy applications.^42^, ^43^, ^44^, ^45^ In addition, a series of novel pyrrolidinium and piperidinium ILs based on cations with two identical monoether groups have been developed which showed promising applications for energy devices.^28^

In order to develop ILs with good physiochemical properties for possible energy applications, we have investigated the effect of cation structural modifications for a family of ILs based on pyrrolidinium, piperidinium and azepanium cations functionalised with two or four ether groups paired with the benchmark [TFSI]^−^ anion. The [TFSI]^−^ anion was utilised, herein, owing to its reasonable cost and commercial availability, and also due to the very good promotion of hydrophobicity and thermal, chemical and electrochemical stability of the resulting ILs. The physiochemical and electrochemical properties of these new ILs have been compared against the corresponding non‐functionalised ionic liquids.

## 2 Results and Discussion

### 2.1 Synthesis of Ether‐functionalised Cyclic Alkylammonium ILs

**Scheme 1:**
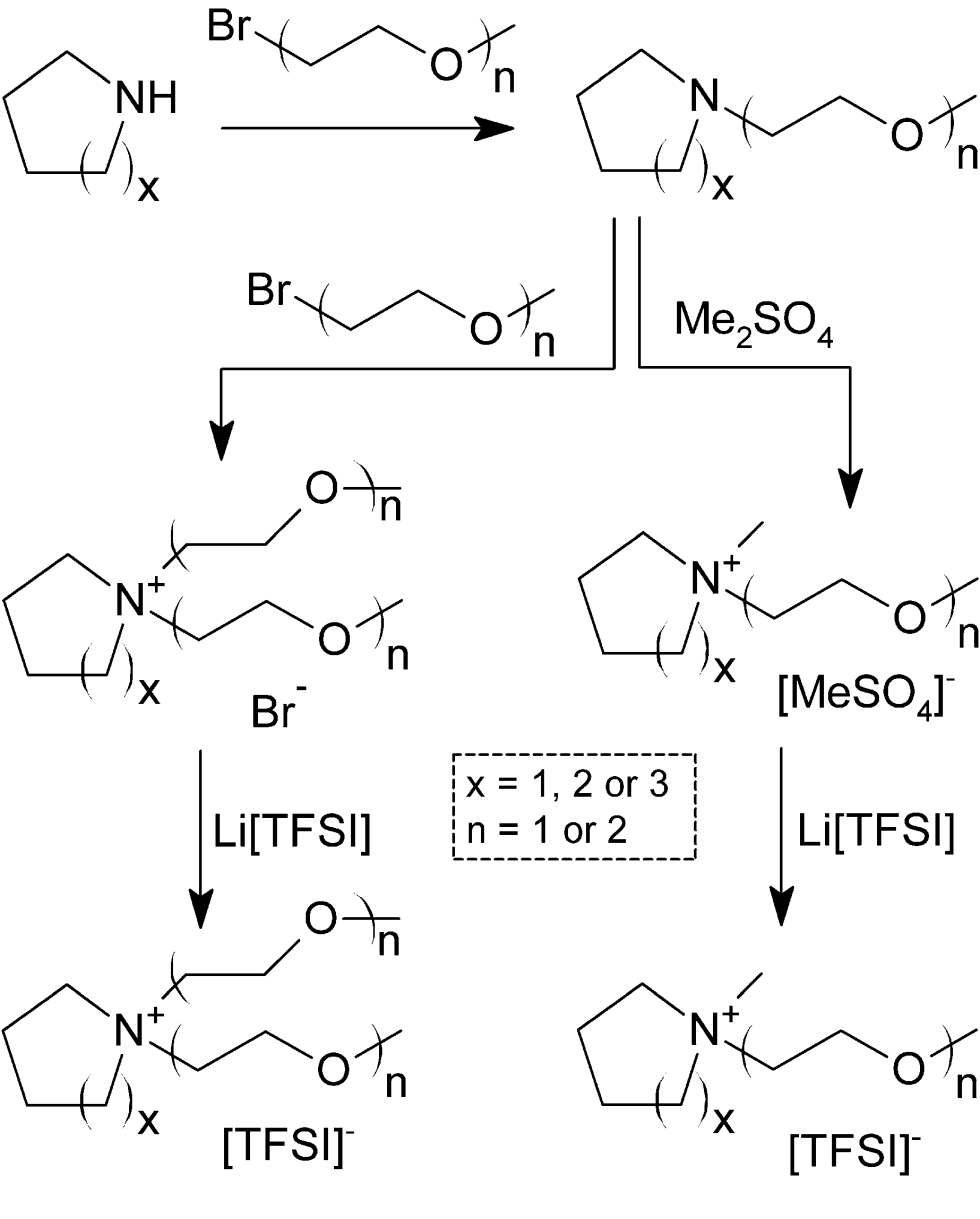
Strategic routes for the synthesis of ether‐functionalised cyclicalkylammonium ILs.

Several synthetic strategies were undertaken to develop these ILs with the maximum yield and purity. Initial attempts to synthesise these materials using conventional alkylation procedures employed for imidazolium‐based ILs proved problematic due to the low alkylation yield observed between the alkylamine and the ethereal precursors. Therein, low to moderate yields of deeply coloured ILs were achieved. Therefore, the proposed strategy was to firstly synthesise the corresponding aminoethereal based species and then perform the alkylation as the final step. These aminoethereal compounds were easily synthesised using the conventional Williamson procedure under aqueous conditions (Scheme 1).

Initially, the secondary cyclic amine (pyrrolidine, piperidine or azepane) was reacted with one molar equivalent of the desired alkylating agent (RBr, where R=butyl, 2‐methoxyethyl or 2‐(methoxyethoxy)ethyl). The reactions were carried out in water which upon completion resulted in the formation of a monophasic system. The corresponding hydrobromide salt was then treated with potassium hydroxide and after stirring for ca. 24 h at room temperature a biphasic system formed. The resulting crude amine was then fractionally distilled in vacuo resulting in good yields (61–71 %). Mono‐functionalised ILs were obtained by reacting these tertiary amine compounds with dimethylsulfate generating the corresponding methylsulfate ILs in almost quantitative yields. Di‐functionalised ILs were obtained by reacting the tertiary amine compounds with either 2‐methoxyethyl or 2‐(methoxyethoxy)ethyl bromide.

These reactions proceeded with lower reactivity due to the reduced nucleophilicty of the N‐centre of the glyme‐functionalised tertiary amines. Metathesis of the bromide or methylsulfate anion‐containing ILs with lithium bis[(trifluoromethyl)sulfonyl]imide (Li[TFSI]) in biphasic water/dichloromethane systems yielded the corresponding [TFSI]^−^ salts in good yields (92–97 %). The prepared [TFSI]^−^ salts were dried in vacuo for at least 48 h and stored in a glove box.

### 2.2 Characterisation of Ether‐functionalised Cyclic Alkylammonium ILs

**Figure 1:**
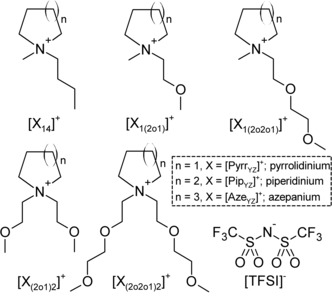
Chemical structures, and respective abbreviations, of the ether‐functionalised cyclic alkylammonium cations and the bis[(trifluoromethyl)sulfonyl]imide, [TFSI]^−^, anion.

This family of ILs were functionalised with different combinations of alkyl and ethereal chains in order to introduce ethereal‐like properties to the liquid. The abbreviations [Pyrr]^+^, [Pip]^+^ and [Aze]^+^ are used to express the cations with 5, 6 and 7‐membered alkylammonium rings, respectively. The subscript text is used to express two additional groups bonded to the nitrogen of the ring; 1=methyl‐, 4=butyl‐, (2o1)=methoxyethyl‐, (2o2o1)=methoxyethoxyethyl‐. The chemical structure of all synthesised ether‐functionalised cations, and their respective abbreviations are shown in Figure 1. Table 1 summarises the thermal behaviour and physiochemical properties at 298.15 K of all the ILs.

**Table 1:** The thermal properties and physiochemical properties at 298.15 K of all the [TFSI]^−^ ionic liquids.

Ionic liquid	*M_r_* [g mol^−1^]	*T*_g_ [K]	*T_d_* [K]	*ρ* [g cm^−3^]	*η* [mPa s]	*σ* [mS cm^−1^]	*Λ* [S cm^2^ mol^−1^]
[Pyrr_14_][TFSI]	422.83		679	1.4054	77.15	2.72	0.818
[Pyrr_1(2o1)_][TFSI]	424.40	187	636	1.4547	49.22	3.69	1.076
[Pyrr_1(2o2o1)_][TFSI]	468.46		643	1.4137	51.19	2.80	0.929
[Pyrr_(2o1)2_][TFSI]	468.46	198	561	1.4308	74.58	1.72	0.563
[Pyrr_(2o2o1)2_][TFSI]	556.56	199	560	1.4010	124.49	1.24	0.494
[Pip_14_][TFSI]	436.86	198	639	1.3775	170.71	1.04	0.329
[Pip_1(2o1)_][TFSI]	438.43	200	630	1.4543	104.46	1.74	0.526
[Pip_1(2o2o1)_][TFSI]	482.49	193	606	1.3956	83.64	1.62	0.560
[Pip_(2o1)2_][TFSI]	482.49	206	631	1.4087	108.01	1.63	0.557
[Pip_(2o2o1)2_][TFSI]	570.59	201	604	1.3531	137.42	0.98	0.411
[Aze_14_][TFSI]	450.89	205	649	1.3729	307.15	0.60	0.198
[Aze_1(2o1)_][TFSI]	452.46	198	641	1.4179	134.87	1.10	0.350
[Aze_1(2o2o1)_][TFSI]	496.52	195	625	1.3949	127.80	1.09	0.388
[Aze_(2o1)2_][TFSI]	496.52	207	605	1.3393	135.74	0.70	0.259
[Aze_(2o2o1)2_][TFSI]	584.62	201	639	1.3527	209.99	0.56	0.241

#### 2.2.1 Thermal Properties

The decomposition temperatures (*T*_d_) of the studied ILs was determined by dynamic thermogravimetric analysis (TGA) wherein the temperature of the sample is increased at a rate of 10 K min^−1^. The *T*_d_ values for range of studied ILs, determined as the temperature at which a 5 % mass loss is recorded, were in the range of 560–679 K. The TGA mass loss traces for each IL are shown in Figure S1 in the Supporting Information. For the appending alkyl group an increase in the number of ether linkages generally resulted in a decrease in thermal stability. The exception to this was the [Aze_(2o2o1)2_][TFSI] which showed increased stability compared to the corresponding [Aze_1(2o1)_]^+^, [Aze_1(2o2o1)_]^+^, [Aze_(2o1)2_]^+^‐based ILs.

A similar study involving imidazolium and morpholinium cations also observed that the replacement of the alkyl group with an ether functionalised group also decreased the thermal stability of the corresponding ionic liquid.^46^

**Figure 2:**
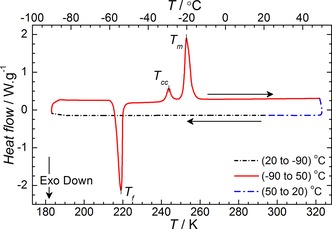
Differential scanning calorimeter (DSC) thermogram of [Pyrr_14_][TFSI].

**Figure 3:**
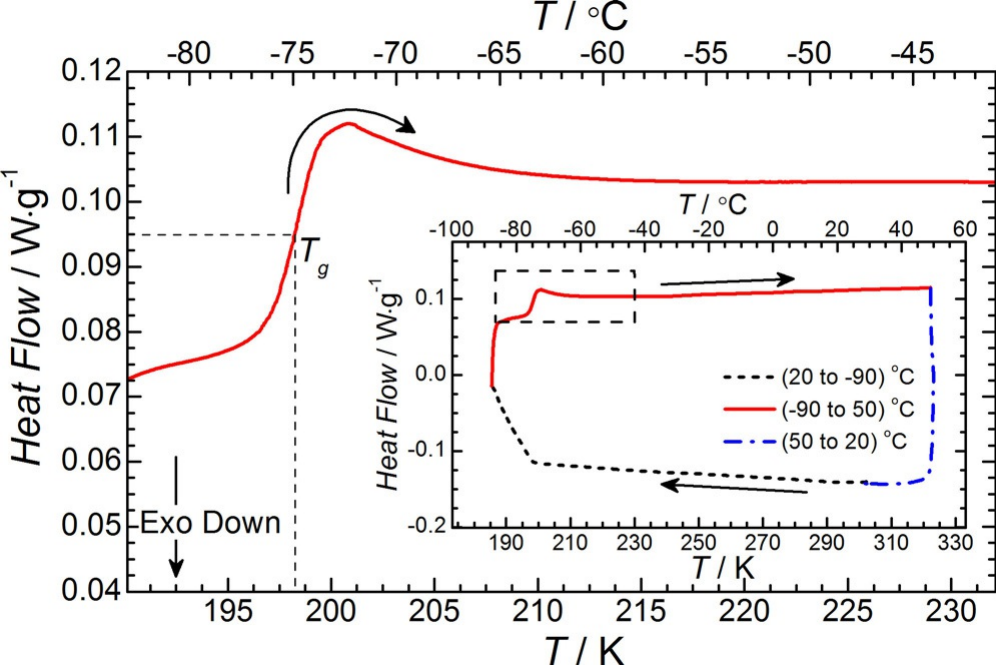
Exemplary DSC thermogram of [Aze_1(2o1)_][TFSI]. Glass transition temperature, *T*_g_, is determined at the midpoint of the curve as highlighted by the dashed lines. Inset graph shows the DSC trace across the full temperature range.

The thermal behaviour of the synthesised ILs was also investigated by differential scanning calorimetry (DSC) within the temperature range of 183.13 K to 323.15 K. Only the [Pyrr_14_][TFSI] IL showed observable transitions associated with crystallisation (*T*_f_) and melting (*T*_m_) (Figure 2). The exotherm (*T*_f_) observed on the heating step is associated with the crystallization of the supercooled liquid at 219 K. Further warming of the sample results in the observation of a small endothermic process (*T*_cc_) at 243.7 K prior to the main endotherm associated with melting (*T*_m_) of the IL at 253 K. The endothermic feature *T*_cc_ is associated with a crystal–crystal phase transition between two metastable phases. The existence of these polymorphs in [Pyrr_14_][TFSI] has been previously reported using DSC^47^ and, more recently, by temperature‐dependent Raman spectroscopy investigations of the phase changes occurring in this IL.^48^ The results of the latter study suggested that the solid–solid phase transition occurs with a suppression of the presence of the *cis*‐conformer of [TFSI]^−^ anions in favour of a system predominantly containing the lower energy *trans*‐[TFSI]^−^ conformer. All of the other ILs investigated showed no observable *T*_f_ or *T*_m_. The majority of the ILs, with the exception of [Pyrr_14_][TFSI] and [Pyrr_1(2o2o1)_][TFSI], showed glass transition temperatures (*T*_g_) between 187 K and 207 K. Figure 3 shows the DSC thermogram of [Aze_1(2o1)_][TFSI] as a representative example of a typical trace observed for these ILs, with the exception of [Pyrr_14_][TFSI] and [Pyrr_1(2o2o1)_][TFSI]. The *T*_g_ value was estimated from the half‐maximum on the rise of the broad endothermic peak. The DSC heating traces of the remaining ILs are presented in Figure S2. Functionalisation of ionic liquid alkyl chains with ether groups has been previously reported to result in a decrease in melting point.^49^ Therein, the reason for the observed *T*_m_ and *T*_g_ depression was thought to be due to the increased rotational freedom and subsequent reduction in lattice energy as has been described elsewhere.^38^ No such clear trend is observed for this family of ILs. For the two ILs which did not show any obvious glass transition, it is likely that the actual *T*_g_ falls outside of the measured temperature range. Notably, slight evidence for a peak associated with a glass transition can be seen in the DSC trace of [Pyrr_14_][TFSI] (Figure 2). Unfortunately, the position of this feature is too close to the minimum temperature of the DSC measurement, ca. 183 K, to be accurately distinguished.

#### 2.2.2 Density

**Figure 4:**
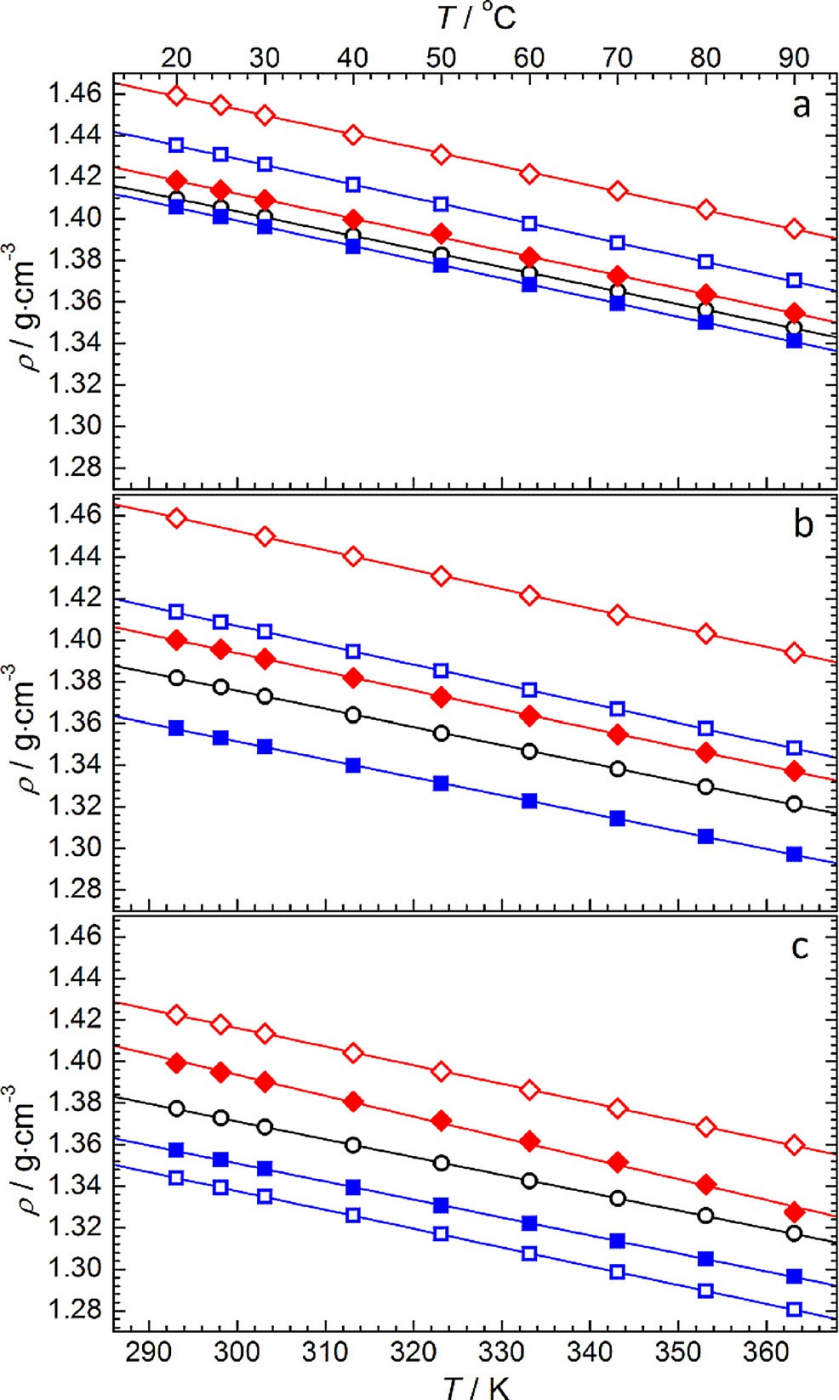
Density variation as a function of temperature for (a) pyrrolidinium, (b) piperidinium and (c) azepanium‐based [TFSI]^−^‐based ILs. Solid lines represent the linear fit of each dataset based on the equation *ρ*=A+BT. Data point symbols represent the functional groups of a given cyclic alkylammonium cation; ○=14, ◊=1(2o1), ♦=1(2o2o1), □=(2o1)_2_, ▪=(2o2o1)_2_.

Density measurements of the alkyl‐ and ether‐functionalised ILs were performed between 293–363 K and the experimental data are summarised in Figure 4. The numerical raw data is presented in Table S1. As is typical for ionic liquids, the density was observed to decrease linearly as a function of temperature. This data has, therefore, been fitted to Equation (1):

(1) ρ=A+BT

where *ρ* is the density (in g cm^−3^), *T* is the temperature (in K) and *A* and *B* are the linear fitting parameters, see Table 2. Regardless of the cationic scaffold, the density distinctly increases in the order of [X_14_][TFSI]<[X_1(2o2o1)_][TFSI]<[X_1(2o1)_][TFSI] (where X represents either pyrrolidinium, piperidinium or azepanium). This observation is in accordance with previous literature values concerning the study of ether‐ and alkyl‐functionalised pyrrolidinium, piperidinium^50^ and azepanium [TFSI]‐based ILs.^31^ Additionally, the specific density values at 298 K for the pyrrolidinium‐ and azepanium‐based ILs are in reasonable agreement with the equivalent literature values (Table 2). In addition, where the alkyl functional groups remain constant, the observed density generally decreases as the cation size increases; in the order [Pyrr_1X_][TFSI]>[Pip_1X_][TFSI]>[Aze_1X_][TFSI]. However, for the ILs functionalised with the methoxyethyl group, a minimal difference is observed between the density of the pyrrolidinium (1.4594 g cm^−3^ at 293 K) and piperidinium (1.4588 g cm^−3^ at 293 K). The values in Table 2 also show that, with the exception of [Aze_1(2o2o1)_][TFSI], the fitting parameters obtained from a linear correlation are also in reasonable agreement with the literature values.

**Table 2:** Density values, *ρ*, of the [TFSI]^−^‐based ILs at 298.15 K and the fitting parameters, *A* and *B*, for the linear fitting equation *ρ=A+BT*.

IL cation	*ρ* [g cm^−3^]	*A* [g cm^−3^]	10^4^ *B* [g cm^‐3^ K^−1^]	Adj. *R* ^2^
[Pyrr_14_]^+^	1.4054 (1.3950)^[a]^	1.6709	−8.9107	0.99993
[Pyrr_1(2o1)_]^+^	1.4547 (1.4546)^[a]^	1.7268	−9.1390	0.99990
[Pyrr_1(2o2o1)_]^+^	1.4137	1.6856	−9.1160	0.99883
[Pyrr_(2o1)2_]^+^	1.4308 (1.39)^[b]^	1.7091	−9.3433	0.99989
[Pyrr_(2o2o1)2_]^+^	1.4010	1.6762	−9.2370	0.99988
[Pip_14_]^+^	1.3775 (1.3786)^[b]^	1.6358	−8.6690	0.99989
[Pip_1(2o1)_]^+^	1.4543 (1.4355)^[b]^	1.7316	−9.3024	0.99994
[Pip_1(2o2o1)_]^+^	1.3956	1.6633	−8.9843	0.99993
[Pip_(2o1)2_]^+^	1.4087 (1.36)^[c]^	1.6865	−9.3173	0.99997
[Pip_(2o2o1)2_]^+^	1.3531	1.6104	−8.6300	0.99994
[Aze_14_]^+^	1.3729 (1.3661)^[d]^	1.6284 (1.6183)^[d]^	−8.5737 (−8.4670)^[d]^	0.99990
[Aze_1(2o1)_]^+^	1.4179 (1.4156)^[d]^	1.6851 (1.6801)^[d]^	−8.9633 (−8.8730)^[d]^	0.99994
[Aze_1(2o2o1)_]^+^	1.3949 (1.3815)^[d]^	1.6947 (1.6404)^[d]^	−10.036 (−8.6893)^[d]^	0.99709
[Aze_(2o1)2_]^+^	1.3393	1.6092	−9.0517	0.99997
[Aze_(2o2o1)2_]^+^	1.3527	1.6108	−8.6610	0.99987

Literature values shown in brackets: [a] Ref. 39, [b] Ref. 50, [c] Ref. 28, d. Ref. 31

Furthermore, the isobaric coefficient of thermal expansion, *α*_p_, for the studied ILs was calculated using Equation (2):

(2) $$\alpha_{p}=\frac{1}{V_{m}}{\left(\frac{\partial V_{m}}{\partial T}\right)}_{p}=-\frac{1}{\rho }{\left(\frac{\partial \rho }{\partial T}\right)}_{p}$$

where *V*_m_, the quotient of molar mass (in g mol^−1^) divided by the density (in g cm^−3^), is the molar volume (in cm^3^ mol^−1^) of the IL. For all of the ILs reported in this work, the value of *α*_p_ lies within the range of 5.7×10^−4^–7.5×10^−4^ K^−1^ across the studied temperature range, with the exception of several seemingly anomalous data points. Notably, *α*_p_ of [Aze_1(2o2o1)_][TFSI] increases to ca. 9×10^−4^–9.9×10^−4^ K^−1^ in the higher temperature region 353–363 K. This significant deviation in *α_p_* is consistent with the apparent lack of linearity observed for the high temperature density of this IL, as represented by the red filled diamonds in Figure 4 c. However, the observed range of coefficients for this set of ILs is in reasonable accordance with previously reported ILs with similar structures (examples given at 298 K): [Pyrr_14_][TFSI], *α*_p_=6.56×10^−4^ K^−1^;^51^ [1‐butyl‐3‐methylimidazolium][TFSI], *α*_p_=7.24×10^−4^ K^−1^;^51^ [Pyrr_14_][thiocyanate], *α*_p_=5.08×10^−4^ K^−1^;^52^ [Pip_14_][thiocyanate], *α*_p_=5.5×10^−4^ K^−1^;^52^ [Pyrr_14_][dicyanimide], *α_p_*=5.56×10^−4^ K^−1^.^53^ The calculated values for *α*_p_ are given for all ILs across the full studied temperature range in Table S2.

#### 2.2.3 Viscosity

**Figure 5:**
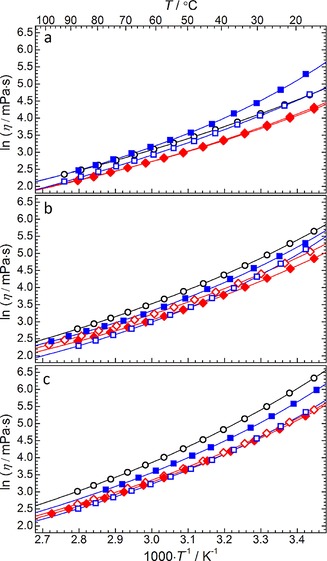
Viscosity variation as a function of temperature for (a) pyrrolidinium, (b) piperidinium and (c) azepanium‐based [TFSI]^−^‐based ILs. Solid lines represent the correlation of the data based on the VTF equation [Eq. (3)]. Data point symbols represent the functional groups of a given cyclic alkylammonium cation; ○=14, ◊=1(2o1), ♦=1(2o2o1), □=(2o1)_2_, ▪=(2o2o1)_2_.

The viscosity of an electrolyte is an important parameter which contributes to the transport capability of the active substances during electrochemical processes. To study the effect of the cation structure on this property, the viscosity of the ILs was measured between the temperature range of ca. 293–363 K. The temperature dependences of the viscosity are presented in the form of Arrhenius‐type plots in Figure 5. The numerical raw viscosity data is presented in Table S3. These plots show that the majority of the ILs do not follow Arrhenius‐type behaviour. As is common for ILs, the temperature dependence of viscosity, *η*, can be well described using the Vogel–Tammann–Fulcher (VTF) Equation (3):

(3) $$\eta =\eta_{o}exp\left(\frac{B^{\eta }}{T-T_{o}^{\eta }}\right)$$

where *η_o_*, *B* ^*η*^ and *T_o_* ^*η*^ are the fitting parameters and *T* is the temperature. The best fit parameters for each IL are listed in Table 3, where *B* ^*η*^ is related to the pseudo activation energy barrier linked to the energy barrier needed to be overcome for ions to move past each other (i.e. *B^η^=E_a_*/*R,* where *R* is the gas constant).^54^ *T_o_* is related to the glass transition temperature of the ionic liquid and, by its position in the VTF equation, the temperature at which the viscosity would approach infinity. Good correlation of the temperature dependence of viscosity of the ILs was achieved with the VTF equation within the measured range.

**Table 3:** Viscosity, *η*, data at 298.15 K and VTF fitting parameters for the ether and alkyl functionalised ammonium [TFSI]^‐^‐based ILs.

IL cation	*η* [mPa s]	*η_o_* [mPa s]	*B^η^* [K]	*T_o_^η^* [K]	Adj. *R* ^2^
[Pyrr_14_]^+^	77.15 (79.6)^[a]^	0.154	846.38	161.98	0.99999
[Pyrr_1(2o1)_]^+^	49.22 (54.8)^[a]^	0.269	633.58	176.56	0.99999
[Pyrr_1(2o2o1)_]^+^	51.19	0.237	654.17	176.46	0.99995
[Pyrr_(2o1)2_]^+^	74.58 (55)^[c]^	0.081	933.84	161.31	0.99989
[Pyrr_(2o2o1)2_]^+^	124.50	0.364	510.70	210.61	0.99973
[Pip_14_]^+^	170.71 (182)^[b]^	0.159	815.32	181.29	0.99999
[Pip_1(2o1)_]^+^	104.46 (102)^[b]^	0.215	718.86	181.92	0.99997
[Pip_1(2o2o1)_]^+^	83.64	0.220	694.30	181.27	0.99992
[Pip_(2o1)2_]^+^	108.01 (122)^[c]^	0.255	548.19	207.51	0.99997
[Pip_(2o2o1)2_]^+^	137.42	0.342	579.01	201.60	0.99963
[Aze_14_]^+^	307.15 (315)^[d]^	0.129	862.49	187.22	0.99999
[Aze_1(2o1)_]^+^	134.87 (160.3)^[d]^	0.206	720.29	187.09	0.99998
[Aze_1(2o2o1)_]^+^	127.80 (136.5)^[d]^	0.174	746.08	185.10	0.99995
[Aze_(2o1)2_]^+^	135.74	0.208	650.48	197.80	0.99997
[Aze_(2o2o1)2_]^+^	209.99	0.186	724.94	195.01	0.99996

Literature values shown in brackets: [a] Ref. 39, [b] Ref. 50, [c] Ref. 28, [d] Ref. 31

Further discussion of the coefficients of the VTF correlation is detailed in a following section. The results demonstrate that the viscosity of the IL tends to increase as the size of the alkyl ring of the cation is increased and in general the viscosity of the IL follows the trend [Pyrr_X_][TFSI]<[Pip_X_][TFSI]<[Aze_X_][TFSI] (where X represents a particular set of functional groups). Comparing ILs of the same cation ring size, the data also shows that substituting the butyl group with a methoxyethyl group reduces the measured viscosity of the IL. The ether group has been previously reported to reduce the viscosity of ILs relative to similarly sized alkyl‐functionalised equivalents.^38^, ^39^, ^50^ This effect has been associated with the flexibility of ether moiety due to the free rotation of the C−O−C bond.^39^ Alternatively, the results of a molecular dynamics study of similarly sized butyl‐ and 2‐methoxyethyl‐functionalised acyclic ammonium ILs suggested that the reduction in IL viscosity is related to changes in cation–cation interactions; that is, short range van der Waals interaction between alkyl chains are more efficient than for the alkoxy‐group equivalent.^55^ However, increasing the length of this ether chain appears to have a varying effect; for pyrrolidinium and azepanium ILs, the viscosity decreases in the order of [X_14_][TFSI]>[X_1(2o2o1)_][TFSI]>[X_1(2o1)_][TFSI]. This behaviour suggests there is a balance between increasing the flexibility of the ether chain (reducing viscosity) and increasing the cation size (increasing viscosity). However, for the piperidinium class of ILs, the introduction of the methoxyethoxyethyl chain yields a very significant drop in the measured viscosity.

The ILs for which the cations have been functionalised with two ether groups ([X_(2o1)2_][TFSI] and [X_(2o2o1)2_][TFSI]) exhibit different behaviours depending on the size of the ring. For the pyrrolidinium‐based ILs, the additional groups lead to a large increase in the IL viscosity from 58.8 mPa s for [Pyrr_14_][TFSI] to 72.9 mPa s and 124.4 mPa s for [Pyrr_(2o1)2_][TFSI] and [Pyrr_(2o2o1)2_][TFSI], respectively. For the piperidinium‐ and azepanium‐based ILs, the effect of the additional ether chains (in place of the original methyl group) does not follow a simple trend. Firstly, the viscosity of [Pip_(2o1)2_][TFSI] and [Pip_(2o2o1)2_][TFSI] is lower than the analogous [Pip_14_][TFSI]. Secondly, compared to [Aze_14_][TFSI], the viscosity of [Aze_(2o1)2_][TFSI] is lower while the viscosity of [Aze_(2o2o1)2_][TFSI] is higher. These observed variations between the ILs with different ring sizes could possibly arise again from a combination of opposing factors which positively or negatively impact the viscosity. Increasing ring size and ether chain length may increase the number of available low energy conformers, thus increasing the potential void space promoting mass transport of the ions, that is, decreasing the viscosity. However, increasing the molecular weight of the cation increases the van der Waals interactions, inhibiting the mass transport leading to an increased viscosity.

#### 2.2.4 Conductivity

**Figure 6:**
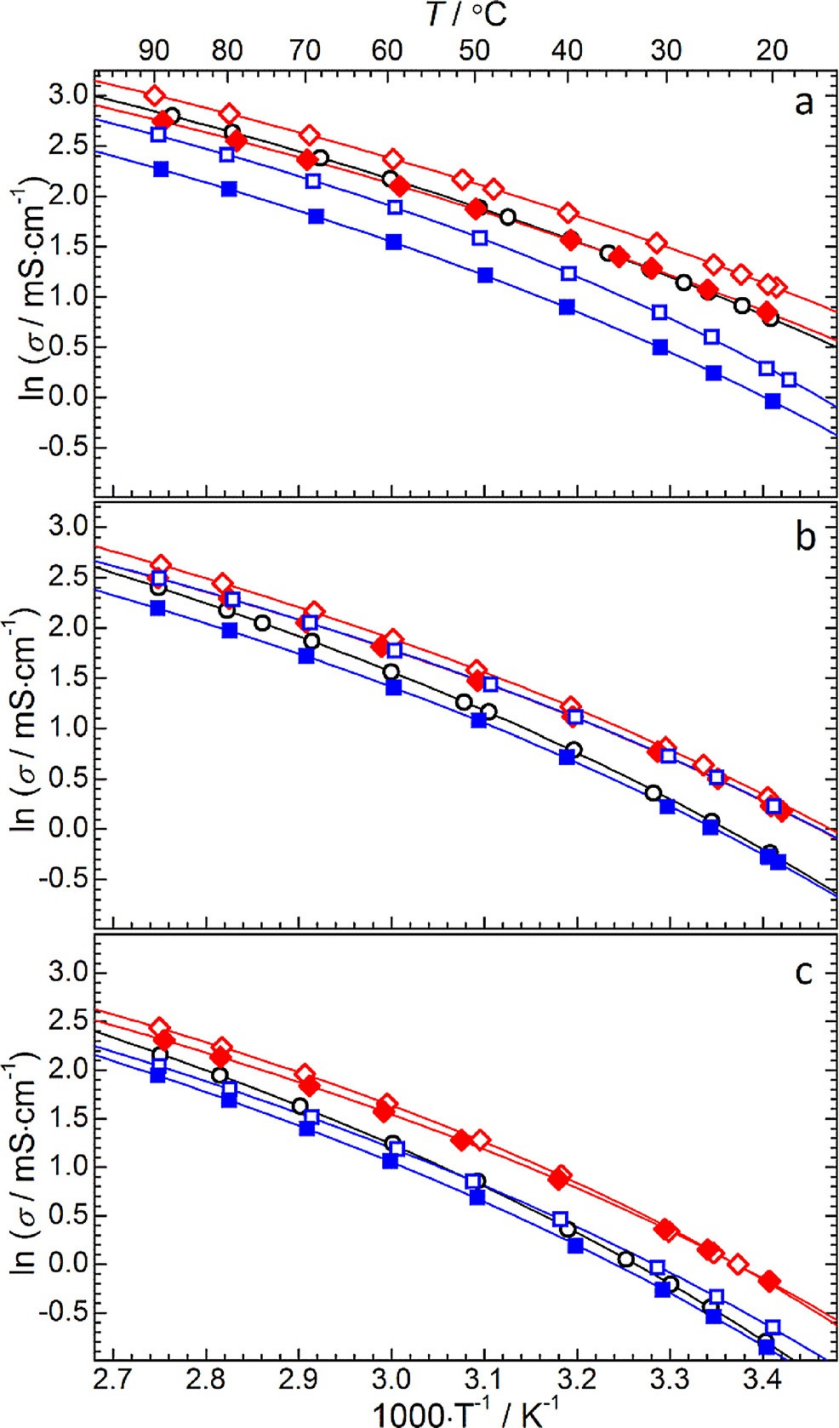
Conductivity variation as a function of temperature for (a) pyrrolidinium, (b) piperidinium and (c) azepanium‐based [TFSI]^−^‐based ILs. Solid lines represent correlation of the data based on the VTF equation [Eq. (4)]. Data point symbols represent the functional groups of a given cyclic alkylammonium cation; ○=14, ◊=1(2o1)=1(2o1), ♦=1(2o2o1), □=(2o1)_2_, ▪=(2o2o1)_2_.

The electrolytic conductivity, *σ*, of the ether‐functionalised ILs was measured across the temperature range of 293–363 K. The experimental results, organised by the size of the alkyl ring of the cations, are presented as Arrhenius‐type plots in Figure 6. The numerical conductivity data are presented in Table S4. The solid lines represent the non‐linear fitting of the data with respect to the VTF Equation [Eq. (4)]:

(4) $$\sigma =\sigma_{o}exp\left(\frac{{-B}^{\sigma }}{T-T_{o}^{\sigma }}\right)$$

where *T* is the absolute temperature and *σ_o_, B* ^*σ*^ and *T_o_* ^*σ*^ represent the fitting parameters. Like viscosity, the VTF fitting equation is commonly used to accurately fit the temperature dependency of the conductivity of ionic liquids which do not follow the Arrhenius Law.^56^

Analogous to the parameters of the VTF equation used to describe IL viscosity in the previous section, *B* ^*σ*^ is related to a pseudo‐activation energy for conduction (*B^σ^= E*_a_/*R,* where *R* is the gas constant) and *T_o_* is related to the glass‐transition temperature of the IL, theoretically representing the temperature at which the conductivity of the system would disappear. Good correlation of the temperature dependence of the conductivity was achieved by correlation with Equation (4). The VTF correlation parameters for the conductivity data of the studied ILs are presented in Table 4.

**Table 4:** Conductivity, *σ*, data at 298.15 K and VTF fitting parameters for the ether and alkyl functionalised ammonium [TFSI]^‐^‐based ILs.

IL cation	*σ* [mS cm^−1^]	*σ_o_* [mS cm^−1^]	*B* ^*σ*^ [K]	*T_o_* ^*σ*^ [K]	Adj. *R* ^2^
[Pyrr_14_]^+^	2.72 (2.62)^[a]^	574.90	672.95	172.47	0.99999
[Pyrr_1(2o1)_]^+^	3.69 (3.72)^[a]^	593.50	666.73	166.94	0.99999
[Pyrr_1(2o2o1)_]^+^	2.80	465.61	658.85	169.28	0.99999
[Pyrr_(2o1)2_]^+^	1.72 (2.29)^[c]^	286.63	496.88	201.04	0.99990
[Pyrr_(2o2o1)2_]^+^	1.24	324.48	622.46	186.28	0.99997
[Pip_14_]^+^	1.04 (1.1)^[b]^	695.27	747.58	183.30	0.99985
[Pip_1(2o1)_]^+^	1.74 (2.0)^[b]^	535.11	661.16	182.68	0.99996
[Pip_1(2o2o1)_]^+^	1.62	390.93	623.07	184.59	0.99996
[Pip_(2o1)2_]^+^	1.63 (1.08)^[c]^	385.28	618.76	184.98	0.99996
[Pip_(2o2o1)2_]^+^	0.98	360.71	647.83	188.59	0.99995
[Aze_14_]^+^	0.60 (0.56)^[d]^	736.69	770.50	189.77	0.99989
[Aze_1(2o1)_]^+^	1.10 (1.12)^[d]^	384.32	576.29	199.80	0.99969
[Aze_1(2o2o1)_]^+^	1.09 (0.97)^[d]^	384.79	623.40	191.88	0.99998
[Aze_(2o1)2_]^+^	0.70	442.69	713.74	187.50	0.99994
[Aze_(2o2o1)2_]^+^	0.56	501.72	756.22	186.96	0.99993

Literature values shown in brackets: [a] Ref. 39, [b] Ref. 50, [c] Ref. 28, [d] Ref. 31

The reported data shows that increasing the alkyl ring size of the cation has a negative effect on the resulting conductivity. This observation corresponds with the increase in viscosity (Table 3, Figure 5) and the fact that increasing the overall cation size is typically linked to a drop in conductivity. Since conductivity is dependent on ionic mobilities (as well as the number of charge carriers), the inverse relationship between the two quantities (conductivity and viscosity) is expected. The effect of substituting the butyl group for the methoxyethyl group results in an increase in conductivity regardless of the alkylammonium ring size. These observations correlate well with the observed reduction in IL viscosity and is attributed to the increased ionic mobility.

Increasing the size of the ether chain, from methoxyethyl to methoxyethoxyethyl, has a negative effect on the conductivity across the measured temperature range. Furthermore, increasing the overall cation size by substituting the methyl group for a second ether functional group yields a small drop in the observed conductivity for the piperidinium‐based ILs but a significant drop in the measured conductance of the pyrrolidinium and azepanium‐based ILs. This observation is likely to be the result of a trade‐off between increasing the degrees of rotational freedom of the flexible ether chains and increasing the size of the cation which reduces the mobility of the cation and hinders the conductivity. As discussed in terms of the measured IL viscosities, there will exist a balance between factors either positively or negatively impacting the conductivity of the IL. In the case of the majority of the cations functionalised with two ether chains, the increased cation size appears to dominate these factors leading to lower conductance than the equivalent IL functionalised with only one ether chain.

This is also consistent with previously reported observations which demonstrate the positive effect of the small flexible alkoxy chains.^39^, ^50^ A further comparison of the effect of the ether functionalisation versus cation size within this class of ILs could be made by measuring the respective properties of dibutyl‐ or diheptyl‐functionalised cations. Such IL cations would be of similar size to the studied diether cations, [X_(2o1)2_][TFSI] and [X_(2o2o1)2_][TFSI].

#### 2.2.5 Ionicity

**Figure 7:**
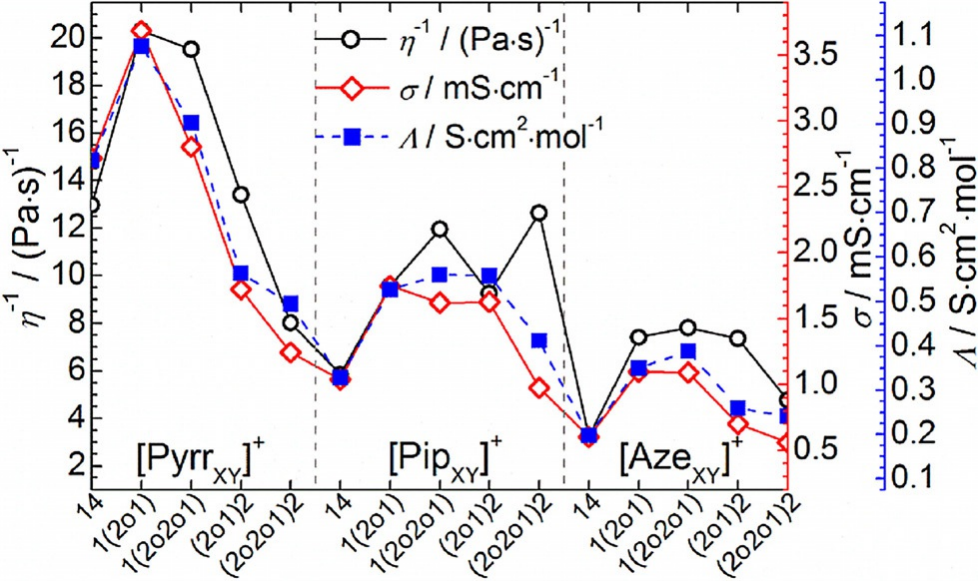
Variations in the fluidity (*η* ^−1^, reciprocal viscosity, left *y*‐axis), conductivity (*σ*, right inner *y*‐axis) and molar conductivity (*Λ*, right outer *y*‐axis) in the studied [TFSI]^−^‐based ILs at 298 K. The *x*‐axis markers highlight the abbreviations of the functional groups X and Y attached to the labelled cyclic alkylammonium cation.

A typical observation made when characterising ILs is the aforementioned inverse relationship between the viscosity and conductivity of the liquid. The cooperative dependency of these behaviours is unsurprising given that viscosity describes a liquids resistance to flow while the conductance of an ionic liquid relies on the mobility of the ions carrying the charge. However, considering the data discussed in the previous sections, the lower viscosities of certain ILs did not directly translate to higher conductivities. For example, the viscosity of [Pip_1(2o2o1)_][TFSI] (83.6 mPa s at 298 K) was lower than its single ether‐group analogue, [Pip_1(2o1)_][TFSI] (108.0 mPa s at 298 K), yet the conductivity of the latter example was found to be higher (1.62 mS cm^−1^ and 1.74 mS cm^−1^ at 298 K for [Pip_1(2o2o1)_][TFSI] and [Pip_1(2o1)_][TFSI], respectively). Some of these variations are highlighted graphically in Figure 7. The lack of a direct trend between viscosity and conductivity shows other factors and properties intrinsic to each ionic liquid are clearly contributing to the viscosity and conductivity in different ways.

**Figure 8:**
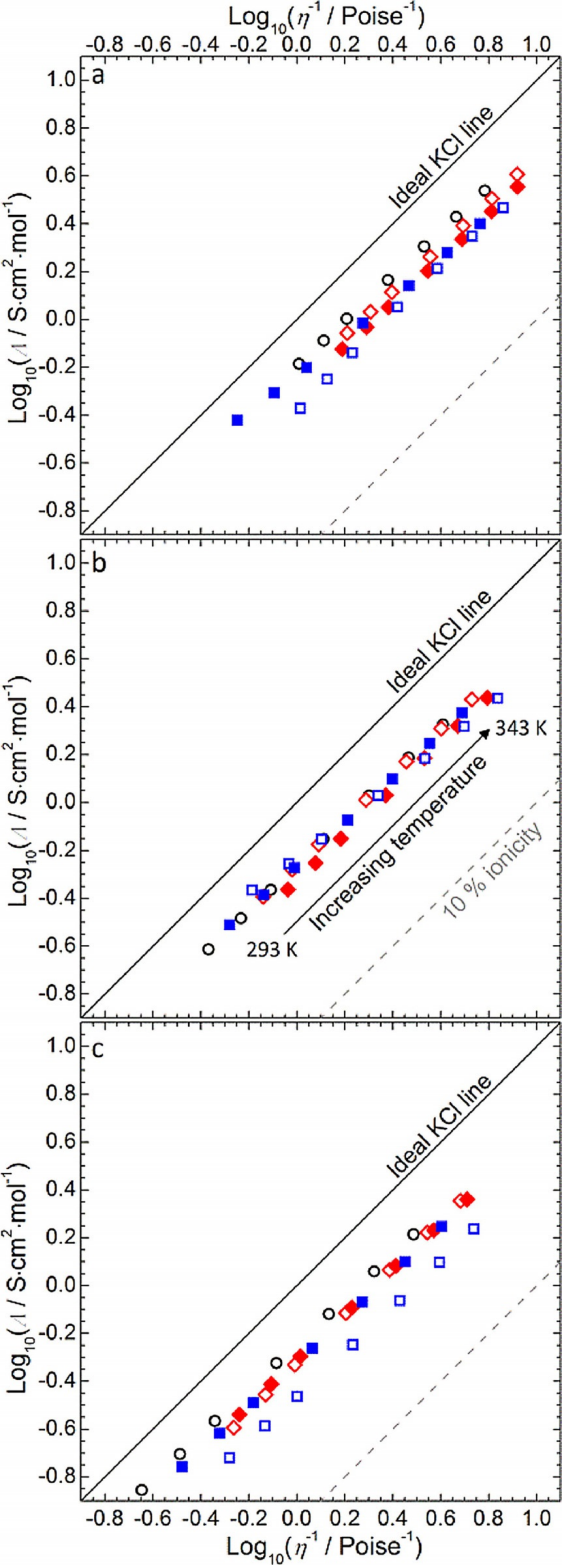
Walden plots for a temperature range of 293–343 K for (a) pyrrolidinium, (b) piperidinium and (c) azepanium‐based [TFSI]^−^‐based ILs. Data point symbols represent the functional groups of a given cyclic alkylammonium cation; ○=14, ◊=1(2o1), ♦=1(2o2o1), □=(2o1)_2_, ▪=(2o2o1)_2_. The solid lines represent the ideal Walden behaviour of an aqueous KCl solution. The dashed lines represent a ΔW of 1 (% *ionicity*=10 %), beneath which ILs are classed as poorly ionic (or poor ionic liquids).

The discrepancies between these quantities have been discussed previously in terms of the ionicity, or degree of ionic association, of the ILs and the Walden rule; *Λη*=constant. This can be straightforwardly represented with the use of a Walden plot; a plot of log(*η* ^−1^) vs. log(*Λ*), where the reciprocal viscosity (or fluidity, *η* ^−1^) is given in Poise^−1^ and the molar conductivity, *Λ*, is given in S cm^2^ mol^−1^. The derived *Λ* values for each IL at 298.15 K are provided in Table 5. The Walden plots within the temperature range of 298–343 K for the 15 ILs are shown in Figure 8. Herein the viscosity and conductivity of each IL is calculated by accurate interpolation of VTF correlation discussed previously and the molar conductivity is calculated by division of electrolytic conductivity by the IL concentration, *c*, (*Λ*=*σ* [S cm^−1^]/*c* [mol cm^−3^]). The IL concentration was calculated by linear interpolation of the temperature‐dependency of the IL density and the IL molecular weight (*c*=*ρ*[g cm^−3^]/ *M_r_* [g mol^−1^]). The calculated values for *Λ* and *η* ^−1^ over a temperature range of 293 K−343 K are presented in Table S5. The solid lines in Figure 8 (a–c) are the ideal KCl lines representing the ideal Walden behaviour taken from extrapolation of the behaviour of a 0.01 mol dm^−3^ KCl aqueous solution, a strong electrolyte where the ions are known to be fully dissociated and considered equally mobile.^57^ Deviations below this calibration line, that is, where the conductivity of the IL is lower than may be expected at a given viscosity according to the Walden rule, imply the existence of significant ionic association between the cation and anion with the tendency to behave as ion pairs. The magnitude of this deviation, Δ*W* (where Δ*W* represents the vertical displacement from the *ideal KCl line*) has been used to classify the ionicity of the liquid.^57^

**Table 5:** The derived molar conductivity, *Λ*, at 298.15 K for each of the alkyl‐ and ether‐functionalised [TFSI]^‐^ ILs. Also shown are the linear fitting coefficients, Log_10_(*c* ^*′*^) and *α_W_*, of Equation (6) for the temperature dependence of Walden behaviour and the *% ionicity* derived from the fractional Walden rule.

IL cation	*Λ* [S cm^2^⋅mol^−1^]	*% ionicity*	Log_10_(*c* ^*′*^)	*α_W_*	Adj*. R* ^2^
[Pyrr_14_]^+^	0.818	64.17	−0.193	0.935	0.99998
[Pyrr_1(2o1)_]^+^	1.076	55.28	−0.257	0.938	0.99992
[Pyrr_1(2o2o1)_]^+^	0.929	49.85	−0.302	0.929	0.99995
[Pyrr_(2o1)2_]^+^	0.563	42.18	−0.375	0.991	0.99907
[Pyrr_(2o2o1)2_]^+^	0.494	58.97	−0.229	0.809	0.99920
[Pip_14_]^+^	0.329	54.89	−0.261	0.959	1.00000
[Pip_1(2o1)_]^+^	0.526	54.81	−0.261	0.947	1.00000
[Pip_1(2o2o1)_]^+^	0.560	47.14	−0.327	0.961	1.00000
[Pip_(2o1)2_]^+^	0.557	59.10	−0.228	0.780	0.99936
[Pip_(2o2o1)2_]^+^	0.411	54.92	−0.260	0.911	0.99978
[Aze_14_]^+^	0.198	56.96	−0.244	0.943	1.00000
[Aze_1(2o1)_]^+^	0.350	47.27	−0.325	1.004	0.99989
[Aze_1(2o2o1)_]^+^	0.388	48.96	−0.310	0.948	0.99998
[Aze_(2o1)2_]^+^	0.259	34.52	−0.462	0.939	0.99986
[Aze_(2o2o1)2_]^+^	0.241	47.98	−0.319	0.927	0.99992

For example, ILs for which Δ*W* is greater than one order of magnitude below ideal Walden behaviour (Δ*W*>1, where Δ*W*=1 is represented by the diagonal dashed lines in Figure 8) lie towards the bottom‐right of the Walden plot and can be classified as poor ionic liquids since the molar conductivity is much lower than possibly expected from a given low viscosity (high *η* ^−1^).^58^ Conversely, if the Walden behaviour of an IL lies closer to the ideal KCl line, where Δ*W* is small, it can be understood that these liquids exhibit more dissociated ionic character in which the constituent ions are more independently mobile.

The data in Figure 8 shows that all the ILs in this study lie below the ideal calibration line, highlighting a certain degree of ionic association or aggregation between the constituent ions of the IL. However, the deviation is not so strong as to classify any of the ILs as poor and Δ*W*<0.4 (% *ionicity*>40 %) for all studied ILs at 298 K, with the single exception of [Aze_(2o1)2_][TFSI] (for which Δ*W*=0.45 and *% ionicity*=35 %). Using the example discussed earlier in this section concerning [Pip_1(2o2o1)_][TFSI] and [Pip_1(2o1)_][TFSI], it can be seen that the IL functionalised with the longer ether chain lies further from the ideal line. This indicates that slightly stronger ionic interactions may be present in [Pip_1(2o2o1)_][TFSI] and possibly account for the fact that this IL exhibits a lower conductivity even though it is less viscous than the analogous piperidinium IL functionalised with the shorter chain.

Furthermore, the temperature dependant Walden behaviour of the ILs (Figure 8) shows that the apparent degree of dissociation is not independent of the temperature according to the Walden rule (i.e. the slope of the data points is not parallel to the ideal KCl line). To account for these variations as a function of the temperature an additional exponent, *α_W_*, can be introduced to give the fractional Walden rule [Eqs. (5), (6)]:

(5) $$\Lambda \eta^{\alpha_{W}}=c{}^{'}=constant$$

(6) $$\log_{10}\left(\Lambda \right)=\log_{10}\left(c{}^{'}\right)+{\alpha_{W}\log}_{10}\left(\eta^{-1}\right)$$

where *c′* is analogous to the aforementioned Walden product. The linear fitting coefficients of Equation (6) and the calculated *% ionicity* as calculated from the fractional Walden rule for each IL are presented in Table 5.

As a result of extrapolation of the seemingly ideal behaviour of aqueous KCl solutions, *α*_W_ is considered to be unity for the ideal KCl line. Conversely, the ionicity of majority of the ILs reported in this work appears temperature dependant and *α*_W_ values between 0.9–0.96 are exhibited, showing that, at increasing temperatures, Δ*W* increases and the IL viscosity decreases at greater rate than the increase in conductivity that may be expected from so‐called ideal Walden behaviour. However, comparison between the *α_W_* values for ILs and dilute electrolytic salt‐in‐solvent solutions has been previously described as largely meaningless; particularly since *real α*_w_ values relating the viscosity and conductivity of aqueous KCl solutions are found to roughly equal 0.87.^59^ Nevertheless, relative comparison of the temperature‐dependant Walden behaviour of ILs shows that the relationship between IL conductivity and fluidity, whereby *α_w_* values roughly equate to 0.9–0.95, is in reasonable agreement with previously reported ILs; for example, [EMIm][TFSI], *α*_w_=0.906;^59^ [EMIm][dicyanamide], *α*_w_=0.95;^59^ [BMIm][TFSI], *α*_w_=0.998;^51^ and [Pyrr_14_]][TFSI], *α*_w_=0.971.^51^

The effects contributing to ion‐pair formation or aggregation will relate partly to delocalisation of the ionic charge surrounding the cation/anion centre and structural contributions of bulky side chains contributing to steric hindrance of coulombic interaction. Within this family of ILs, the negative charge of the bulky [TFSI]^−^ anion is highly delocalised via the electron withdrawing (trifluoromethane)sulfonyl groups, reducing affinity for ion‐pair formation. Within the cyclic alkylammonium cations, generally the apparent *% ionicity* is greatest for the alkyl‐functionalised cations. Where ether functionality is introduced and the cation remains of similar size (i.e. butyl groups substituted by the 2‐methoxyethyl group), electron donation from the lone pairs of the ethereal oxygen atoms would be expected to contribute somewhat to the localisation of the positive charge and, in turn, increase the propensity for ionic interaction with the counter ion. We reported a similar reduction in apparent ionic dissociation for ether vs. alkyl functionalised cyclic sulfonium [TFSI]^−^‐based ILs based on the tetrahydrothiophenium cation.^60^ For larger degrees of ether functionalisation, the tendency for ion–ion pair formation would be further dependant on additional factors including steric hindrance, organisation/freedom and interaction of flexible ether chains, varying ring conformers and anion solvation/interaction in competition with ethereal groups and cation centres. As such, a complex variation in the *% ionicity* is not unexpected.

#### 2.2.6 VTF Parameters and Fragility

Table 6 shows a comparison of the *T*_g_ derived from DSC measurements and the VTF derived parameter, *T_o_* ^*η/σ*^, from temperature dependant viscosity and conductivity measurements. The VTF parameters should agree if both charge transport properties, viscosity and conductivity, are closely coupled. However, the derived VTF fitting parameters are very sensitive to small deviations within the measurements and, as such, direct comparison can become problematic. Nevertheless, in many cases, both *T_o_* values show a reasonable match for the studied ILs and the most significant discrepancies appear with the diether‐functionalised ILs. Furthermore, due to the intricate relationship between IL viscosity and conductivity (or more specifically molar conductivity), differences in the activation energy barriers for the two processes should also be related to changes in the apparent ionicity of the ILs with temperature (i.e. *α*_W_).^61^ Since typical Arrhenius plots [ln(*η*) or ln(*Λ*) vs. 1/*T*] of the viscosity and molar conductivity of ILs yield non‐linear behaviour, it is inferred that the apparent activation energy derived from Arrhenius equations [Eqs. (7), (8)] is temperature dependant.

(7) $$\eta =A_{\eta }exp\left(\frac{E_{a}^{\eta }}{RT}\right)$$

(8) $$\Lambda =A_{\Lambda }exp\left(\frac{{-E}_{a}^{\Lambda }}{RT}\right)$$

**Table 6:** Comparison of measured *T_g_* and the VTF correlation parameters, *T_o_* ^*η*/*σ*^, from the viscosity and conductivity measurements for each of the alkyl‐/ether‐functionalised [TFSI]^‐^‐based ILs. The ratio between Arrhenius‐type activation energies, *E*_a_^*Λ*^/*E*_a_^*η*^, for viscosity and molar conductivity is shown and the value in brackets represent the percentage deviation between *E*_a_^*Λ*^/*E*_a_^*η*^ and *α*_W_. The calculated fragility indices, *m*_*η*/*σ*_, and the ratios between measured *T_g_* and the *T_o_* ^*f*^ correlation parameters of the modified VTF equation [Eqs. (9) and (10)] are also shown.

IL cation	*T*_g_ [K]	*T_o_* ^*ηf*^ [K]	*T_o_* ^*σf*^ [K]	*E*_a_^*Λ*^ */ E*_a_^*η*^		*m*_*η*_	*m*_*σ*_	*T*_g_/*T_o_* ^*ηf*^	*T*_g_/*T_o_* ^*σf*^
[Pyrr_14_]^+^	186^[b]^	161.98	172.47	0.94	(−0.1)^[a]^	124^[c]^	126^[c]^	1.15^[c]^	1.13^[c]^
[Pyrr_1(2o1)_]^+^	187	176.56	166.94	0.93	(0.4)^[a]^	147	142	1.12	1.12
[Pyrr_1(2o2o1)_]^+^		176.46	169.28	0.93	(0.3)^[a]^				
[Pyrr_(2o1)2_]^+^	198	161.31	201.04	1.01	(−2.0)^[a]^	151	141	1.12	1.12
[Pyrr_(2o2o1)2_]^+^	199	210.61	186.28	0.79	(2.9)^[a]^	134	141	1.14	1.12
[Pip_14_]^+^	198	181.29	183.30	0.95	(0.8)^[a]^	126	119	1.15	1.14
[Pip_1(2o1)_]^+^	200	181.92	182.68	0.95	(0.2)^[a]^	153	125	1.11	1.14
[Pip_1(2o2o1)_]^+^	193	181.27	184.59	0.99	(−2.8)^[a]^	138	129	1.13	1.13
[Pip_(2o1)2_]^+^	206	207.51	184.98	0.78	(0.0)^[a]^	153	170	1.12	1.09
[Pip_(2o2o1)2_]^+^	201	210.92	188.59	0.92	(−1.1)^[a]^	139	134	1.13	1.12
[Aze_14_]^+^	205	187.22	189.77	0.93	(1.4)^[a]^	125	119	1.15	1.15
[Aze_1(2o1)_]^+^	198	187.09	199.80	0.97	(3.5)^[a]^	133	118	1.14	1.15
[Aze_1(2o2o1)_]^+^	195	185.10	191.88	0.94	(0.7)^[a]^	125	117	1.15	1.15
[Aze_(2o1)2_]^+^	207	197.80	187.50	0.93	(1.2)^[a]^	154	139	1.12	1.12
[Aze_(2o2o1)2_]^+^	201	195.01	186.96	0.90	(2.5)^[a]^	121	115	1.15	1.15

[a] the percentage deviation between *E*_a_^*Λ*^ */ E*_a_^*η*^ and *α_W_* shown in Table 5, [b] literature value,^47^ [c] calculated using literature *T_g_* value.

where *R* represents the gas constant (8.3145 J mol^−1^ K^−1^) and *A*_*η*_/*A*_*Λ*_ and *E*_a_^*η*^/*E*_a_^*Λ*^ represent the pre‐exponential factor and the activation energy barrier, respectively for the two quantities, viscosity and molar conductivity. As such, following a procedure described previously,^61^ the Arrhenius plots for each respective quantity were firstly fitted by a third‐order polynomial within the range of 293–343 K. By subsequent differentiation of the derived polynomial equation, the gradient (*E*_a_/*R*) was calculated at a series of tangents to the curve. The activation energy (i.e. *E*_a_=*R⋅*gradient) was then taken as an average of the calculated values within the stated temperature range. The coefficients of the polynomial expansions and the derived average *E*_a_ values are shown in Tables S6 and S7. Interestingly, the ratio between the two activation energies, *E*_a_^*Λ*^/*E*_a_^*η*^ (detailed in Table 6) closely represent the derived values for the exponent of the fractional Walden rule, *α*_W_. In fact, the majority of the values for the studied ILs differ by less than 2 % (bracketed values in Table 6). This is in agreement with the observations, and the original hypothesis of this relation, detailed by Schreiner et al.^61^

As mentioned previously, the *T_o_* values derived from VTF correlation of viscosity and conductivity data are related to the IL′s glass transition temperature, *T*_g_ (as measured by DSC). Typically for many ILs, *T_o_* usually falls approximately between 10 and 50 K below the *T*_g_ of the IL.^61^, ^62^ However, the larger differences between *T*_g_ and the theoretical *T_o_* are considered to depend on the liquid fragility or strength. Furthermore, while lower values of *T*_g_ would be expected to yield lower viscosity, or higher conductivity, it has been shown for ILs under ambient conditions that these properties are dependent on both fragility and *T*_g_.^58^, ^63^ The concept of fragility, introduced by Angell,^64^, ^65^ describes the rate at which the transport properties and relaxation dynamics of a glass‐forming material changes as the temperature approaches *T*_g_. Materials described as highly fragile, or low strength, exhibit higher susceptibility to structural change and, in turn, large changes in transport properties over small temperature variations close to the *T*_g_.

Conversely, strong materials are considered more impervious to structural changes upon heating above their *T_g_*.^61^ Ionic liquids, materials known for undergoing glass transitions and commonly inadequately described by normal Arrhenius‐type temperature dependence (in terms of transport properties), have been frequently described as exhibiting intermediate to high fragility, such that their transport and relaxation dynamics change several orders of magnitude over relatively small temperature deviations close to their *T*_g_.^58^, ^61^, ^63^, ^66^, ^67^

**Figure 9:**
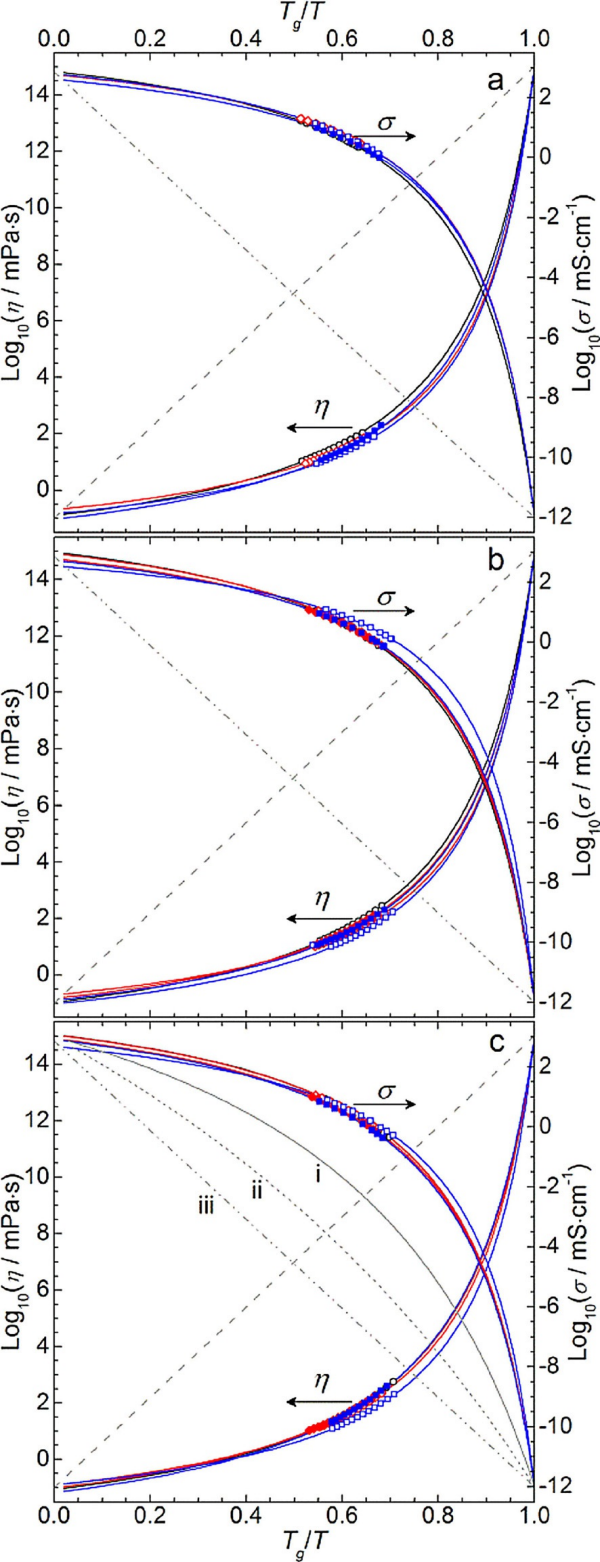
*T*_g_/*T* scaled Angell‐plots for viscosity (left *y*‐axis) and conductivity (right *y*‐axis) behaviour of the (a) pyrrolidinium, (b) piperidinium and (c) azepanium [TFSI]^−^‐based ILs. Data point symbols represent the functional groups of a given cyclic alkylammonium cation; ○=14, ◊=1(2o1), ♦=1(2o2o1), □=(2o1)_2_, ▪=(2o2o1)_2_. Solid, coloured lines represent correlation by the modified VTF equation [Eqs. (9) and (10)]. Diagonal dashed and dash‐dot‐dot straight lines represent ideal Arrhenius type dependence of a strong materials and additional curves in (c) represent the effect of varying m/D: i) D=15, *m*=54; ii) D=50, *m*=27; iii) *m*=15.

To assess the apparent fragility of the studied ionic liquids, quantified by the fragility index *m*, the viscosity and conductivity data was further correlated as a function of temperature by a modified VTF equation [Eqs. (9), (10)]:

(9) $$\eta =\eta_{o}^{f}\exp\left(\frac{D^{\eta }T_{o}^{\eta f}}{T-T_{o}^{\eta f}}\right)$$

(10) $$\sigma =\sigma_{o}^{f}\exp\left(\frac{-D^{\sigma }T_{o}^{\sigma f}}{T-T_{o}^{\sigma f}}\right)$$

where *D* ^*η*/*σ*^ represents the strength parameter, inversely proportional to the fragility index, *m*, and the remaining parameters are equivalent to the previously described VTF equation [Eqs. (3) and (4); additional notation of a superscript *f* is included here to differentiate between parameters used in these and the previous equations]. For each dataset, an additional point of *η*(*T*_g_)=10^15^ mPa s or *σ*(*T*_g_)=10^−12^ mS cm^−1^ was included in the correlation leading to slightly different correlation parameters. These values represent an approximation of the sample viscosity and conductivity, respectively, at the *T*_g_ of the material.^61^, ^63^ The correlation parameters of these fits, detailed in Table S8, are then used to extrapolate the temperature dependant behaviour of viscosity/conductivity towards the *T*_g_. This is presented in the form of an Angell‐type plot (Figure 9), where the temperature along the *x*‐axis is scaled by the material's *T*_g_. The fragility index, *m*, calculated using Equation (11) (and by differentiation of the fitted slope as *T*→*T*_g_), is representative of the gradient of approach of each respective property towards the *T*_g_ (higher *m* corresponds to a steeper approach, greater changes in viscosity/conductivity as *T* approaches *T*_g_ and a higher fragility). Full derivation of Equation (11) is explained in Ref. 61.

(11) $$m=\frac{{DT}_{o}}{ln10}\cdot \frac{T_{g}}{{\left(T_{g}-T_{o}\right)}^{2}}$$

Within the Angell plots of Figure 9, an approximation of ideal Arrhenius‐type behaviour of the transport properties of a strong (or non‐fragile) material is represented by the diagonal dash and dash‐dot‐dot straight lines. For viscosity, this represents a minimum fragility of 16 determined by the logarithmic ratio of the viscosity at *T*_g_ and an approximation of the limiting viscosity (*m*_min_=Log_10_[*η*(*T_g_*)/*η_o_* ^*f*^]=16 where *η_o_* ^*f*^ ≈0.1 mPa s). This trend is supported by the behaviour of strong glass formers, for example, SiO_2_ and GeO_2_.^58^ For the conductivity plot, since the limiting conductivity is generally considerably more varied, the *y*‐axis intercept of the hypothetical Arrhenius line on the Angell plots was approximated from the average *σ_o_* ^*f*^ correlation parameter for the studied ILs [i.e. *σ_o_* ^*f*^=2.86 mS cm^−1^, Eq. (10)]. This yielded an approximate value of 15 for the fragility index of the ideal Arrhenius line for conductivity.

The data presented in Figure 9 shows the studied ILs all exhibit very similar, high fragility behaviour. This similarity is expected considering the shared anion and the closely related cationic structures. The values for *m*_*η*/*σ*_ derived from the viscosity and conductivity data are shown in Table 6 and, in ranging from 115–170, are of a similar order to values previously reported for fragile ILs.^61^, ^63^, ^68^ Furthermore, the values for the fragility indices derived from the two different quantities, viscosity (*m*_*η*_) and conductivity (*m*_*σ*_), show reasonable agreement between their magnitudes and respective trends. Within the different ring‐size families of ILs studied, no clear trend is observed. However, for all cation ring sizes, the bis(2‐methoxyethyl) functionalised ILs (e.g. [Pip_(2o1)2_][TFSI]) appear to exhibit the highest fragilities; explaining why the ambient temperature fluidity/conductivity of these ILs is not too drastically low, despite possessing some of the highest *T*_g_ values (particularly the piperidinium and azepanium ILs).

Conversely, the fragility indices of the butyl/methyl functionalised ILs (e.g. [Pyrr_14_][TFSI]) tend to lie at the lower end of the measured values. Though the deviation of derived values is relatively small, and all studied ILs would be classed as highly fragile, the lower apparent fragility of the dialkyl‐functionalised cations could be attributed to more efficient van der Waals interactions between alkyl substituents which are effectively dampened by substitution with ether groups.

Following the analysis of the data by Equations (9) and (10), values of the derived coefficients *T_o_* ^*ηf*^ and *T_o_* ^*σf*^ for viscosity and conductivity, respectively, consistently lie within a range of 17.6–27.4 K below the measured *T*_g_. Furthermore, the ratios between these coefficients and the measured *T*_g_ of the studied ILs (*T*_g_/*T_o_* ^*ηf*^ and *T*_g_/*T_o_* ^*σf*^) are shown in Table 6. An average value for *T*_g_/*T_o_* ^*ηf*^ of 1.13±0.004 is obtained from both viscosity and conductivity derived coefficients. All of these ratios satisfy the original observation of Angell and described by Equation (12):

(12) $$\frac{T_{g}}{T_{o}^{f}}=1+\frac{D}{m_{min}\cdot ln\left(10\right)}$$

where *m*_min_ refers to the minimum fragility index for each respective property (*m*_min_=16 and 15 for the viscosity and conductivity data, respectively).^65^

#### 2.2.7 Electrochemical Stability

The electrochemical stability of the alkyl and ether functionalised cyclic alkylammonium [TFSI]^−^ ILs was determined by cyclic voltammetry (CV) at a 3 mm diameter glassy carbon (GC) macrodisk electrode. Under dry, and inert conditions of an Ar‐filled glovebox, the potential of the working electrode cycled at 2 mV s^−1^ until a current density boundary of ±0.5 mA cm^−2^ was recorded, at which point the potential sweep was reversed. The temperature of all CV measurements was ca. 303±1 K as dictated by the internal atmosphere of the glovebox. The electrochemical windows of the studied ILs are presented in full in Figure S3 in the ESI. The CVs of the studied ILs all show the onset of massive electrolyte decomposition by reduction and oxidation in the regions of (−3.4 to −3.0) V vs. Fc^+^/Fc and (1.7 to 2.6) V vs. Fc^+^/Fc. Furthermore, additional small oxidation peaks are observed in many of the scans at ca. (0.4 to 0.6) V vs. Fc^+^/Fc. These are generally attributed to the oxidation of small quantities of protic impurities (possibly residual water). However, by varying the voltage/current limits of the initial reducing sweep (which is always completed before the oxidising sweep), we were able to show that the existence of this small additional current response is related to the oxidation of products formed during reductive decomposition of the IL. Figure S4 in the ESI shows an exemplary CV in [Pip_1(2o2o1)_][TFSI] wherein the reductive current limits are sequentially increased. The CV traces show that the magnitude of the impurity peak at ca. 0.7 V vs. Ag[NO_3_]/Ag increases as more charge is passed on reduction. In fact, there appears to be a proportionality between the two processes (as shown in Figure S4a, inset graph) and the oxidative integral charge of the impurity peak corresponds to ca. 22–25 % of the total charge passed on the reductive sweep.

**Figure 10:**
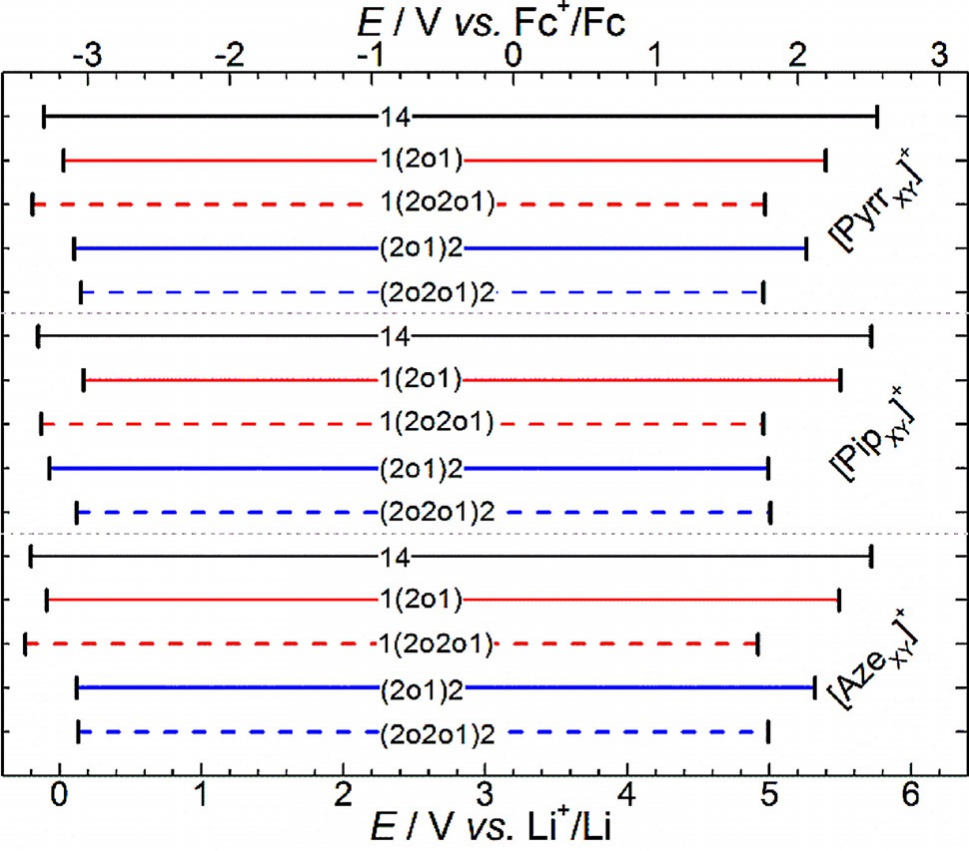
Graphical representation of the electrochemical stability limits of the [TFSI]^−^‐based ILs at a GC electrode vs. Fc^+^/Fc (top *x*‐axis) and Li^+^/Li (bottom *x*‐axis) reference potentials. The abbreviations on each horizontal line represent the functional groups, X and Y, attached to the labelled cyclic alkylammonium cation.

The electrochemical stability window, defined as the potential range between the reductive and oxidative decomposition potentials of the electrolyte, is indicative of the stable operating voltage of the material in electrochemical systems. The wide potential window exhibited by many different IL structures is one of the most attractive features of applying ILs as electrolytes in electrochemical energy storage devices like Li‐batteries and high‐voltage electrochemical double layer capacitors (EDLCs).^16^, ^69^, ^70^, ^71^, ^72^ For EDLC devices in particular, extension of the maximum operative voltage (*U*_max_) range, typically limited by the electrolyte formulation, can result in direct enhancement in device specific energy (*E_Sp_*) and specific power (*P*) capabilities due to the proportionalities; *E_Sp_*=0.5⋅C⋅*U*_max_^2^ and P=*U*_max_^2^/(4⋅*ESR*) where *C* and *ESR* represent the capacitance and equivalent series resistance, respectively.^70^ The onset potentials of reductive and oxidative decomposition (*E*_a_ and *E*_c_, respectively) of the IL electrolytes studied herein are approximated using a cut‐off current density of ±0.1 mA cm^−2^. As such, the electrochemical stability window (Δ*E*) is defined by the difference between these upper and lower potential limits (i.e. Δ*E*=(*E*_a_−*E*_c_) V). The numerical potential limits determined by cyclic voltammetry are presented in Table 7 and represented graphically in Figure 10. With the exception of the bis(methoxyethoxyethyl) functionalised ILs (e.g. [Pyrr_(2o2o1)2_][TFSI]), the studied ILs show electrochemical windows greater than 5 V, wherein reductive decomposition occurs close to the Li^+^/Li reduction potential and oxidative decomposition occurs at ca. 4.9–5.9 V vs. Li^+^/Li. As described in the experimental section, an internal ferrocene redox couple was used to normalise the reference potential vs. the Li^+^/Li reduction potential following determination of electrochemical windows (based on the approximation *E*_Fc+/Fc_ ≈3.2 V vs. *E*_Li+/Li_). An exemplary CV of the ferrocene redox couple versus the Ag[NO_3_]/Ag reference and versus a Li‐metal reference electrode is shown in Figure S5a in the ESI to justify this approximation.

**Table 7:** Onset potentials of anodic (*E_a_*) and cathodic (*E_c_*) decomposition and the respective electrochemical stability windows, Δ*E*, for the [TFSI]^‐^‐based ILs as determined by cyclic voltammetry at a GC working electrode.

IL cation	*E*_c_ [V vs. Li^+^/Li]	*E*_a_ [V vs. Li^+^/Li]	Δ*E* [V]
[Pyrr_14_]^+^	−0.11	5.76	5.87 (5.8)^[a,f]^
[Pyrr_1(2o1)_]^+^	0.03	5.40	5.37 (5.0)^[b,f]^
[Pyrr_1(2o2o1)_]^+^	−0.19	4.97	5.16
[Pyrr_(2o1)2_]^+^	0.10	5.26	5.16 (5.0)^[c,e]^
[Pyrr_(2o2o1)2_]^+^	0.15	4.96	4.81
[Pip_14_]^+^	−0.15	5.72	5.87^[g]^
[Pip_1(2o1)_]^+^	0.17	5.50	5.33
[Pip_1(2o2o1)_]^+^	−0.13	4.96	5.09
[Pip_(2o1)2_]^+^	−0.07	4.99	5.06 (5.5)^[c,e]^
[Pip_(2o2o1)2_]^+^	0.12	5.01	4.89
[Aze_14_]^+^	−0.20	5.72	5.92 (6.5)^[d,e]^
[Aze_1(2o1)_]^+^	−0.09	5.49	5.58 (5.5)^[d,e]^
[Aze_1(2o2o1)_]^+^	−0.24	4.92	5.16 (5.5)^[d,e]^
[Aze_(2o1)2_]^+^	0.12	5.32	5.20
[Aze_(2o2o1)2_]^+^	0.13	4.99	4.86

Literature values shown in brackets: [a] Ref. 78, [b] Ref. 79, [c] Ref. 28, [d] Ref. 31. [e] measured at a GC working electrode, [f] measured at a platinum carbon working electrode, [g] analogous IL 1‐methyl‐1‐propylpiperidinium [TFSI]^−^ reported window of 5.9 V in Ref. 12.

The data presented in Table 7 and Figure 10 straightforwardly shows some apparent trends in the electrochemical stabilities of the studied ILs. Firstly, as may be expected, the dialkyl functionalised ILs, [Pyrr_14_][TFSI], [Pip_14_][TFSI] and [Aze_14_][TFSI], exhibit the largest reductive and oxidative stabilities and, in turn, the largest electrochemical windows (ca. 5.9 V). Beyond this, increasing the degree of ethereal group functionalisation on the three alkylammonium rings appears to yield subsequent reductions in the magnitude of the electrochemical windows, Δ*E*. Specifically, substitution of butyl groups for the 2‐methoxyethyl group reduces *E*_c_ slightly but affects a larger reduction in the oxidative stability, *E*_a_. Lengthening of this ethereal group (e.g. from [Pyrr_1(2o1)_]^+^ to [Pyrr_1(2o2o1)_]^+^) for all three ring sizes promotes a further significant reduction in the oxidative stabilities of the studied ILs. This, and the large degree of variation in oxidative stability of the remaining ILs, is interesting since the more traditional consensus aligns that the oxidative decomposition is generally attributed to oxidation of the anion (and vice versa for the cathodic reductive limits).^73^, ^74^ However, molecular dynamics (MD) simulation and density functional theory (DFT) calculations have suggested this may not always be so straightforward.^75^, ^76^, ^77^ To compare the trends in oxidative and reductive stabilities of the studied ILs as a function of the variation in cation structure, the HOMO/LUMO energy levels of many geometrically optimized conformers of cations, anions and ion pairs were determined from DFT calculations. An extensive summary of the calculated HOMO/LUMO energy levels and surface diagrams of single ions and ion pairs is provided in Table S8 in the ESI.

Within the cation ring size families, increasing ether functionality has almost identical effects on the computed HOMO and LUMO values based on ion calculations; that is, HOMO: [X_14_]^+^<[X_1(2o1)_]^+^≈[X_(2o1)2_]^+^ <[X_1(2o2o1)_]^+^≈[X_(2o2o1)2_]^+^; and LUMO: [X_14_]^+^≈[X_1(2o1)_]^+^<[X_(2o1)2_]^+^<[X_1(2o2o1)_]^+^ <[X_(2o2o1)2_]^+^.

However, the functional group modifications described affect a much larger change in HOMO values (e.g. ≈3 eV range between lowest cation, [Pyrr_14_]^+^=−12.39 eV, and highest cation, [Pyrr_(2o2o1)2_]^+^=−9.46 eV) than the computed single ion LUMO values (where the range is ≈0.7 eV). For the analogous functional group pairing, the effect of the cation ring size on the computed values is generally small but the LUMO follows the trend of [Aze_X_]^+^≳[Pip_X_]^+^≳[Pyrr_X_]^+^, in approximate agreement with trends in observed *E*_c_ values. Furthermore, the agreements in the computed LUMO values and *E*_c_ limits can be further extended to include our previously reported experimental and computational observations for a series of [TFSI]^−^ ILs based on ether‐functionalised acyclic sulfonium cations.^77^ These sulfonium based ILs undergo reductive decomposition at ca. 1 V higher under identical conditions and the computed LUMO values for the sulfonium cations are ca. 1 eV more negative than calculated for the cyclic alkylammonium cations reported here.

Additionally, the trend in cation HOMO values is the same approximate trend observed for *E*_a_. A plot of the computed cation HOMO values vs. the experimentally observed oxidative limits (see Figure S6) for each IL displays good linear correlation; wherein only [Pip_(2o1)2_][TFSI] appears an outlier. However, given that the electrochemical window of an IL is limited by the least stable component in both oxidative or reductive potential regions, the variation in cation HOMO values does not alone justify the observed *E*_a_ trends since the single ion HOMO values computed for the [TFSI]^−^ anion component are much higher (i.e. less stable towards oxidation; −4.19 eV, −3.97 eV and −3.89 eV for the three [TFSI]^−^ conformers). However, we recently reported similar experimental and computational observations for the series of ether‐functionalised acyclic sulfonium [TFSI]^−^ ILs.^77^ There are several possible explanations for the prominent effect of the cation structure on the oxidative stability of the IL. Firstly, the cation may undergo direct oxidation at the electrode interface. Given the well‐structured nature of IL electrical double layers, the presence of cationic species directly at highly positive electrode surface appears counter‐intuitive. However, the long and flexible 2‐(methoxyethoxy)ethyl group will somewhat change the organisation of any IL double layer with this ether group. Further, the observed *E*_a_ of these ILs is similar to the oxidative stability of analogous glyme type solvents (e.g. tri/tetra ethylene glycol dimethyl ether; 4.6–4.9 V vs. Li^+^/Li)^80^, ^81^ indicating the possibility of the terminal ethereal oxygen (where the cation HOMO resides, see surface diagrams in Table S8) behaving more as a free ether group.

A second rationale relates to many possible changes in cation‐anion interactions as a function of the increasing ether functionality. In a previous section, we demonstrated using the Walden behaviour that, with a few exceptions, the ionicity (or degree of dissociation) of the ILs decreased with the introduction of more ethereal groups. Electron donation from the ethereal oxygen atoms into the nitrogen centre of the cation decreases the delocalisation of the positive charge and, in turn, the increased tendency for the formation of ion pairs and the associated restricted freedom of the ions may increase the possibility of direct participation of the cation during oxidative decomposition. Additionally, increased localisation of the cation charge contributes to reductions in the partial positive charge of the alpha methyl/methylene protons and reductions in the computed cation LUMO values. However, in terms of oxidative stabilities, the increased Coulombic sharing of available orbitals between cation and anion trough ion‐pair formation would be expected to increase HOMO energy levels relative to single ion computed levels, that is, reducing the anodic potential barrier of HOMO oxidation and reducing oxidative stability of the IL. Additionally, in our observations reported for the ether‐functionalised sulfonium [TFSI]^−^ ILs,^77^ the DFT calculations of ion pairs indicated that when increasing the level of ether‐functionality on the sulfonium cation, the *cis*‐conformer of the [TFSI]^−^ anion becomes more favoured over the *trans*‐conformer. Based on the single ion DFT calculations, *cis*‐[TFSI]^−^ should exhibit a lower electrochemical oxidative stability (by 0.22 eV) than the *trans* alternative. However, while the comparative ion‐pair simulations of the alkyl and ether functionalised ammonium‐based ILs did not reveal a clear‐cut change in the global minimum energy conformer of the [TFSI]^−^ anion, the relative energy difference between *trans‐* and *cis*‐ conformers was lower for [Pyrr_1(2o2o1)_][TFSI] than for [Pyrr_14_][TFSI] (see Table S9). We relate this observation, in conjunction with the reduced apparent ionicity through increased cation‐anion interaction, to the restriction of the anion positioning around the cation centre as a result of the ether chain presence; that is, interaction between anion and ethereal oxygen atoms is unfavourable and, thus, the freedom of ion coordination around the cation centre is reduced. Consequently, the probability of the less electrochemically stable *cis*‐[TFSI]^−^ conformers existing in the ether functionalised IL is higher than may be expected for the alkyl functionalised analogue. In the chosen example, the calculated HOMO values for [Pyrr_14_][*trans*‐TFSI] and [Pyrr_1(2o2o1)_][*cis*‐TFSI] were −6.626 eV and −6.191 eV, respectively. Coupling this set of calculations with the observed reduction in ionicity of the ether functionalised [Pyrr_1(2o2o1)_][TFSI] IL (15 % less than [Pyrr_14_][TFSI]) can possibly account for the reduced oxidative stabilities observed. A third consideration relates to the possibility of the ether‐functionalised cations readily reacting with any radical products of [TFSI]^−^ oxidation. Such a process would be autocatalytic and somewhat enable a reduction in the oxidative potential barrier required to proceed the reactions. Ultimately, the overall processes may be combinations of the discussed ideas and we are currently devising experimental procedures to understand the reactions taking place during decomposition to make sense of the prominent effects of cation structure.

#### 2.2.8 Li‐redox in Pyrrolidinium ILs

**Figure 11:**
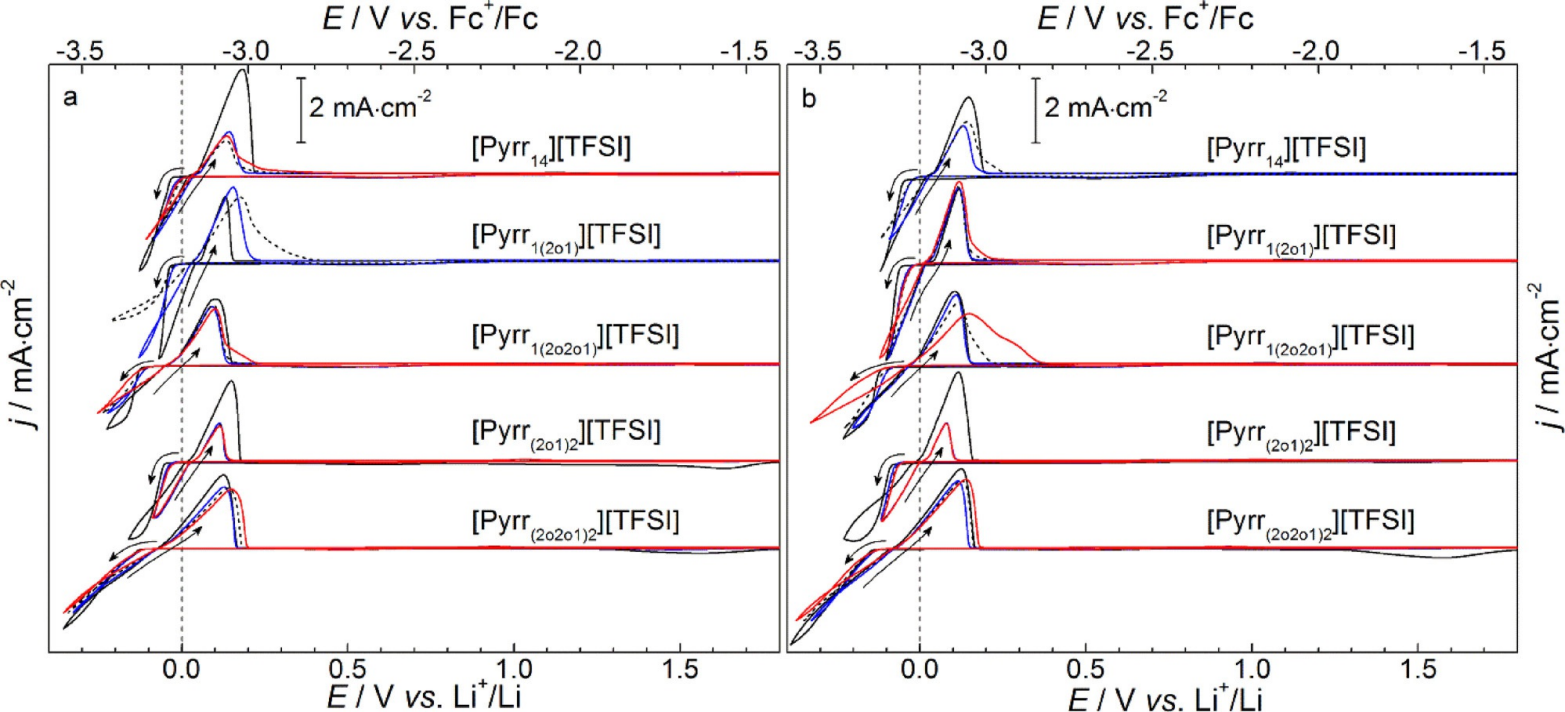
Cyclic voltammograms of Li electrodeposition/stripping in (0.9)IL—(0.1)Li‐salt binary mixtures at a Cu working electrode for the 5 pyrrolidinium‐based ILs containing (a) Li[TFSI] (left panel) and (b) Li[FSI] (right panel) as the Li‐salt component, respectively. The 1st (—), 10th (—), 20th (‐ ‐ ‐ ‐) and 30th (—) cycles are shown and the IL component is given above each respective trace. The arrows show the scan direction. The scan rate was 10 mV s^−1^ and the potentials are displayed vs. Fc^+^/Fc (top *x*‐axis) and vs. Li^+^/Li (bottom *x*‐axis).

One of the most desirable applications for electrochemical stable ILs, in addition to EDLC electrolyte components, is as electrolytes for alkali metal batteries, notably Li and Na. To utilise Li/Na‐metal anodes in place of traditional graphitic anodes, the electrolyte must facilitate deposition and stripping of the alkali metal ions with a high cycling stability. In the context of electrodeposition and stripping of Li, the effect of the cation ether‐group functionality on the Li‐redox chemistry in the family of pyrrolidinium‐based ILs is explored in preliminary CV experiments at a Cu working electrode. Herein, binary mixtures (by mole fraction) of (0.9)IL–(0.1)Li‐salt compositions were prepared with Li[TFSI] and lithium bis(fluorosulfonyl)imide (Li[FSI]) as the Li‐salt component. These molar fraction compositions, corresponding to ca. 0.25–0.18 mol kg^−1^ Li‐salt for the different ILs, were selected to limit the impact of the Li‐salt component on the transport properties of the IL. Further, Li[FSI] was selected as a comparative salt as it displays improved conductivity in IL/salt mixtures relative to Li[TFSI] in these ILs,^82^ and the [FSI]^−^ also shows evidence of good SEI (solid electrolyte interphase) formation during Li anode plating/stripping processes^12^, ^36^ and also during intercalation into Li‐ion graphitic anodes.^30^, ^82^ Figure 11 shows the 1^st^, 10^th^, 20^th^, and 30^th^ cycles of CV experiments at a Cu macrodisk working electrode performed at 10 mV s^−1^ under the inert atmosphere of the Ar‐filled glovebox with (a) Li[TFSI] and (b) Li[FSI] as the salt component. All ILs studied, herein, display cathodic Li metal deposition at potentials more negative than −3.2 V vs. Fc^+^/Fc which is succeeded by an anodic stripping current peak on the reverse scan. As for the measurement of the electrochemical windows at the GC electrode, the reference potentials were normalised initially vs. an internal Fc^+^/Fc couple in each sample and further normalised using the approximation *E*_Fc+/Fc_ ≈3.2 V vs. *E*_Li+/Li_. Likewise, exemplary CVs of the Fc^+^/Fc couple vs. the Ag[OTf]/Ag and Li‐metal reference electrodes at the Cu working electrode are presented in Figure S5b in the ESI to support this approximation.

During the first cycles, independently of the Li‐salt component, the onset of Li electrodeposition occurs at ca.‐30 to −60 mV vs. Li^+^/Li in the [Pyrr_14_]^+^, [Pyrr_1(2o1)_]^+^ and [Pyrr_(2o1)2_]^+^‐based ILs. On the reverse scan, following a characteristic nucleation loop, anodic oxidation associated with stripping of the new Li surfaces begins at ca. 0–50 mV vs. Li^+^/Li for the same ILs. However, one of the most obvious differences between the electrolyte mixtures can be seen for the ILs functionalised with the methoxyethoxyethyl‐group, [Pyrr_1(2o2o1)_][TFSI] and [Pyrr_(2o2o1)2_][TFSI]. In these mixtures, the onsets of the cathodic deposition and anodic stripping currents shift to more negative potentials vs. the Fc^+^/Fc potential by ca. 100 mV. This shift is consistent with a previous analogous system containing Li[TFSI] in [Pip_1(2o2o1)_][TFSI],^41^ and is more pronounced for both examples containing to two ether chains, [Pyrr_(2o2o1)2_][TFSI]. This behaviour prompts the consideration that the secondary ethereal oxygen in the cation functional groups (i.e. the oxygen furthest from the positive nitrogen centre) is involved significantly in the Li^+^‐ion solvation complex in these ILs. Raman/NMR investigations have shown previously that substitution of the butyl group for the short methoxyethyl group enables direct IL‐cation‐Li^+^ interactions (via the weak Lewis basic ethereal oxygen atom), reducing the coordination number of [TFSI]^−^ anions which form the Li^+^‐solvation sphere.^83^ Furthermore, the presence of the longer ether groups in a *N*‐ethyl*‐N*,*N*,*N*‐tri[2‐(2‐methoxyethoxy)ethyl]ammonium‐[TFSI] IL has been recently reported to promote the solubility of Na[TFSI] and display correlated relaxation dynamics between IL and Na^+^ ions, indicative of significant interactions, and potential cation‐cation cross‐linking, between alkali metal cation and IL cation.^43^ As such based on these interesting observations, and the apparent increased cycling stability of the di[2‐(2‐methoxyethoxy)ethyl]‐functionalised IL (relative to [Pyrr_1(2o2o1)_][TFSI]), further investigations in the IL/Li‐salt interactions and the effects of IL‐salt concentrations in these ILs are ongoing.

Beyond the first cycle in all studied ILs, the onset of Li electrodeposition shifted to less cathodic potentials slightly but the potential to drive the electrodeposition process at higher currents shifted to more cathodic potentials, associated with evolution of higher overpotentials required to drive the process. Similarly, with progressive cycling, anodic shifts of the stripping peak can be observed for most of the IL formulations. However, with both Li[TFSI] and Li[FSI] salts, the [Pyrr_(2o1)2_][TFSI] IL displayed uniform cycling up to 30 cycles; albeit with very low charge utilisation. This performance, and the apparent reversibility of the [Pyrr_(2o2o1)2_][TFSI] IL, is interesting given these di‐ether functionalised ILs display some of the poorest cathodic stability limits (see Figure 10, Table 7) at the GC electrode. Evidence for the apparent stabilisation of some [TFSI]^−^‐based ILs towards reductive decomposition in the presence of dissolved Li‐salts has, however, been reported numerous times previously.^28^, ^41^, ^79^

The best cycling performance, taking into consideration stability on repeat cycling and the maximum utilised current densities, appears to be the combination of [Pyrr_1(2o1)_][TFSI] with Li[FSI] salt. This may be expected given this IL exhibits the best transport properties from the all ILs described in this work, the positive Li^+^ conducting SEI film‐forming properties of the [FSI]^−^ anion,^12^, ^36^ and the good stripping/plating performance reported previously for this IL.^79^ However, the applicability of these ILs in for Li‐metal systems (and possibly Na systems also) could be further optimised by investigation of alternative salts, salt concentration ranges and SEI film forming additive compounds.

## 3 Conclusions

The synthesis and systematic thermophysical and electrochemical investigations of 15 cyclic alkylammonium [TFSI]^−^‐based ILs with and without varying degrees of ether functionality is reported. All the studied ILs exhibit low meting temperatures and high thermal stabilities. Ether functionality on the cation slightly reduces upper limits of thermal stabilities but all ILs exist in the liquid phase under wide range of potential operating temperatures for electrochemical devices (e.g. −20 to >100 °C). The transport properties, relating to the IL viscosity and conductivity, are strongly affected by cation structure. Initial substitution of alkyl groups for like‐sized ether groups affords important reductions in IL viscosity and enhances IL conductivities observed, in line with previous observations. However, increasing the degree of ether functionality engages more complex trends in viscosity and conductivity depending on cation ring size. For example, the longer flexible ether chains may increase degrees of rotational freedom and reduce cation–cation interactions, increasing IL fluidity but, in most cases, reducing IL conductivity. The bulky side chains obviously increase cation size, likely reducing ionic mobilities (most prominent for the small pyrrolidinium cations), but also increase the propensity for ion‐pair formation, as evidenced by the assessment of the Walden‐type behaviour for the studied ILs. Herein, all but one of the studied ILs exhibited >40 % *ionicity* and may be classed as *good ionic liquids* but, as stated, the introduction of ether functionality at the cation generally affected reductions in the observed ionicity. Furthermore, estimations of the IL fragilities were completed using extrapolation of both the viscosity and conductivity measurements towards glass transition temperatures. These results further highlighted the coupled nature of both physical properties and revealed the studied ILs to be highly fragile materials; a desirable quality for IL electrolytes.

Furthermore, all of the studied ILs exhibited wide electrochemical windows (>4.5 V), an important feature for potential IL electrolytes. Nevertheless, initial substitution of the alkyl groups for ether groups reduces the magnitude of the observed windows slightly and further lengthening of the ether group yields greater reductions. Most interestingly, despite the ILs sharing the equivalent [TFSI]^−^ anion, the modifications of the cation structure affect the greatest changes in the oxidative stability of the IL, contrary to the classical understanding. Calculations of the HOMO/LUMO energies of single ion conformers reveal the least oxidatively stable orbital of the cation lies on the terminal oxygen of the ether groups but the calculated magnitudes still indicate the lowest thermodynamic pathway towards IL oxidation should be removal of the electron from the HOMO of the [TFSI]^−^ anion. However, increasing ether functionalisation at the cation, firstly, increases the propensity for ion‐pair formation (Walden behaviour) and, secondly, increases the geometric stability of the less electrochemically stable *cis*‐[TFSI]^−^ conformer (DFT calculations). These factors may contribute to the observed reduction in the oxidative stability limits of the ILs but further experimental work is required to fully understand the sequence of events occurring at the electrode/electrolyte interface during electrochemical decomposition of the IL. Preliminary investigations also revealed, or further supported, the applicability of these ILs for plating/stripping processes at Li‐metal anodes. Some of these results were indicative of interesting IL‐cation—Li‐cation interaction effects observed with higher degree of ether‐group functionalisation, prompting further investigations into these IL/Li‐salt compositions.

## Experimental Section

### Materials

The materials used to synthesise the ionic liquids were sourced as follows: pyrrolidine (99 %), piperidine (99 %) *N*‐butylpyrrolidine (99 %), dimethyl sulfate (>99 %), 1‐bromobutane (98 %), ferrocene (98 %) ex Sigma–Aldrich; azepane (99 %) ex INVISTA; 2‐bromoethyl methyl ether (99 %) and 1‐bromo‐2‐(2‐methoxyethoxy)ethane (95 %) ex Fluorochem; lithium bis{(trifluoromethyl)sulfonyl}imide (battery grade) ex 3M All synthesis reagents were used as received. Lithium bis(fluorosulfonyl)imide (<99.5 %) used for Li‐redox experiments was sourced from Kanto Chemical Co. Inc.

### Ionic Liquid Synthesis

Full description of the synthesis of the 15 studied ILs is provided in the Supporting Informatoin in conjunction with results of NMR, Microanalysis, and Li‐content analysis.

### Preparation of Materials for Physiochemical Measurements

Prior to all physical and electrochemical measurements, all ILs were dried under vacuum (ca. 10^−3^ mbar) at 353–373 K with stirring for at least 2 days. This treatment was found to reduce the water content to below 100 ppm, as measured by Karl Fischer coulometric titration. The resolution of the water content measurements was 0.001 wt/wt % (or 10 ppm) and measurements were completed in duplicate. Once dried, the IL samples were stored in the Ar‐filled glovebox with moisture levels less than 3 ppm water. Lithium content of the ILs was analysed by inductively coupled plasma optical emission spectroscopy (ICP‐OES) on an Agilent 5100 ICP‐OES, and along with Microanalysis, was performed by Analytical Services at Queen's University, Belfast. ^1^H and ^13^C NMR spectra were recorded at 293 K on a Bruker Avance DPX spectrometer at 300 MHz and 75 MHz, respectively. ^1^H and ^13^C NMR spectra for all [TFSI]^−^ ILs are shown in Figures S7 to S36 in the ESI.

### Physical Measurements

Density measurements were performed using a DM40 oscillating tube density meter (Mettler Toledo, ±1×10^−4^ g cm^−3^) in the range of 293.15–363.15 K (±0.1 K). The instrument was cleaned using acetone and dried using dehumidified air prior to any measurements. The viscosity of the ILs was measured using a Bohlin Gemini Rotonetic Drive 2 cone and plate rheometer (±1 %) from 293–363 K (±0.01 K) at atmospheric pressure. The rheometer was calibrated using ultra‐pure water and an oil viscosity standard (ASTM Oil Standard S600, Cannon, 1053 mPa s at 298.15 K). Dynamic thermal decomposition profiles were collected by thermogravimetric analysis (TGA) using a TGA Q5000 (TA Instruments). Heating profiles were performed at a rate of 10 K min^−1^ under nitrogen flow from room temperature to 773 K (±1 K). Thermal phase transitions were recorded using differential scanning calorimetry (DSC) analysis on a DSC Q2000 (TA Instruments) with ca. 5 mg IL samples in hermetically sealed Al pans. A heating gradient of 5 K min^−1^ was used and 183 K and 323 K (±0.1 K) were used as the lower and upper temperature limits, respectively.

Conductivity measurements were conducted using a sensION+ EC71 benchtop meter with a 3‐pole platinum sensION+ 5070 conductivity probe (<0.5 % of range) with an in‐built Pt1000 temperature probe (Hach Lange). Regular calibration of the probe was completed using aqueous KCl standard conductivity solutions (147 μS cm^−1^, 1413 μS cm^−1^, and 12.88 mS cm^−1^ at 298 K). The immersion and sealing of the conductivity probe in the liquid sample was carried out in an Ar‐filled glovebox. The conductivity probe, when disconnected from the meter, was immersed in the liquid sample (ca. 3 cm^3^) inside a glass sample tube also containing a small magnetic stirrer bar. The sample was sealed with the probe in the glass tube using an O‐ring seal and Parafilm. The conductivity of the sample was then recorded with stirring as a function of temperature (using the temperature reading built into the conductivity probe). The temperature of the sample was varied from 293–363 K (±0.2 K) using a small oil bath and a hot‐plate with a thermocouple control. The sample conductivity and temperature were recorded when the observed values were stable for ca. 1 min.

### Electrochemical Measurements

All electrochemical measurements of the electrochemical windows and the Li‐redox chemistry were completed using cyclic voltammetry using either an Autolab PGSTAT 302 workstation (Metrohm) or a VMP3 multi‐channel workstation (BioLogic). A custom 3 port glass cell was prepared in‐house which allowed completion of all electrochemical measurements with ca. 1 cm^3^ of electrolyte. Cell preparation and electrochemical measurements were conducted inside the Ar‐filled glovebox. The temperature of the electrochemical measurements was ca. 303 K, as determined by the internal atmosphere of the glovebox. For the determination of electrochemical windows, the working electrode was a 3 mm diameter glassy carbon macrodisk electrode (ALS Co., Ltd). For Li‐redox chemistry, the working electrode was a 1.6 mm diameter copper macrodisk electrode (ALS Co., Ltd). Prior to all measurements, the working electrodes were cleaned with acetonitrile and ultra‐pure water and then polished using alumina slurries of decreasing grain size (1.0 μm, 0.3 μm and 0.05 μm, Buehler) in ultra‐pure water on soft microcloth lapping pads (Kemet Ltd.). Following the polishing, the working electrodes were sonicated in ultra‐pure water for 2 minutes. The GC electrode was dried in an oven at ca. 353 K and the Cu electrode was dried under a nitrogen stream. The counter electrode was a platinum wire coil heat sealed in a glass capillary and the reference electrodes used were based on a silver wire in a 0.1 mol dm^−3^ solution of either Ag[NO_3_] in 1‐butyl‐3‐methylimidazolium nitrate or silver trifluoromethanesulfonate (Ag[OTf]) in [Pyrr_14_][TFSI] separated from the bulk solution by a glass frit. The reference potentials were normalized vs. an internal ferrocene redox couple wherein a small amount of ferrocene was dissolved in the IL following completion each measurement. Cyclic voltammetry was then used to approximate the formal potential of the ferrocene/ferrocenium redox couple. To verify the approximation of the reference potential vs. Li^+^/Li, a small quantity of Li[TFSI] was dissolved in the ferrocene‐containing IL and a small strip of fresh Li‐metal was used as the reference electrode for cyclic voltammetry measurements of the ferrocene couple.

For the measurements of Li‐redox chemistry, both Li[TFSI] and Li[FSI] salts were firstly dried at 373 K under vacuum (ca. 10^−3^ mbar followed by ca. 10^−5^ mbar, turbomolecular vacuum pump station) for several days. IL/Li‐salt binary mixtures were then prepared accurately by mass inside the Ar‐filled glovebox with molar fractions of (0.9)IL—(0.1)Li‐salt and left to stir overnight to ensure complete dissolution. The cells were then prepared using the Cu working electrode, the Pt‐wire counter electrode and the Ag[OTf]/[Pyrr_14_][TFSI] reference electrode and CV was used at a 10 mV s^−1^ scan rate to assess Li plating/stripping with repeated cycling. Therein, the cathodic current density was limited to −3 mA cm^−2^ or, alternatively, to a portion (ca. 3/4 to 2/3 ) of the maximum peak current observed on the first cycle. Following completion of the cycling, the reference potential was normalised vs. the ferrocene couple as described previously.

### Computational Methods

Calculations of single‐ion and ion pair HOMO and LUMO energies were completed using the Turbomole 7.0 program package,^84^ as described by our group previously.^77^ Before the visualization of the HOMO/LUMO orbitals using TmoleX (version 4.1.1), single ion structures were optimized in the gas phase, with a convergence criterion of 10^−8^ Hartree, using DFT calculations combining the Resolution of Identity (RI) approximation^85^, ^86^ within the Turbomole 7.0 program package using the B3LYP function with the def‐TZVP basis set.^87^, ^88^, ^89^ The resulting optimized structures were then used as inputs in the COSMOconfX program (version 4.0) to generate the conformers of each species. Single point energy calculations (DFT/B3LYP/def‐TZVP + RI approximation) within Turbomole were then utilized to determine the HOMO/LUMO orbitals of the predefined conformers of each species.

## Conflict of interest

*The authors declare no conflict of interest*.

## Supporting information

As a service to our authors and readers, this journal provides supporting information supplied by the authors. Such materials are peer reviewed and may be re‐organized for online delivery, but are not copy‐edited or typeset. Technical support issues arising from supporting information (other than missing files) should be addressed to the authors.

Supplementary
