# Supplementary material for: Thermophysical and Electrochemical Properties of Ethereal Functionalised Cyclic Alkylammonium‐based Ionic Liquids as Potential Electrolytes for Electrochemical Applications
Source: Chemphyschem. 2017 Jun 21;18(15):2040–57. doi: 10.1002/cphc.201700246 (PMC5575513; doi:10.1002/cphc.201700246)
Supplement: Supplementary file 1 — Supplementary [file CPHC-18-2040-s001.pdf]

CHEMPHYSCHEM

Supporting Information

Thermophysical and Electrochemical Properties of Ethereal Functionalised Cyclic Alkylammonium-based Ionic Liquids as Potential Electrolytes for Electrochemical Applications

Alex R. Neale,*[a] Sinead Murphy,[a] Peter Goodrich,[a] Christopher Hardacre,[a, b] and
Johan Jacquemin*[a, c]

cphc_201700246_sm_miscellaneous_information.pdf

The ionic liquids were synthesized according to the procedures described below.

Synthesis of the tertiary amines

Under an inert atmosphere, the haloalkane (1-bromobutane, 2-bromoethyl methyl ether or 1-bromo-2-(2-methoxyethoxy)ethane, 0.1 mol) was added dropwise to a stirring solution of amine (pyrrolidine, piperidine or azepane, 0.1 mol) in deionised water (100 cm^3) cooled in an ice water bath. Four hours later potassium hydroxide (11.2 g, 0.2 mol) was then added to the reaction mixture and the mixture was vigorously stirred under reflux for 24 h. The resulting suspension was then filtrated and the solution was then concentrated under reduce pressure in a rotary evaporator to leave the crude product.

1-Butylpiperidine

1-Butylpiperidine was obtained as a colourless liquid after distillation in vacuo (59-60 °C @ 10.0mbar), 9.48 g, 63% yield. ^{1}H NMR (300 MHz, $CDCl_3$): δ 2.45 – 2.51 (m, 6H), 1.74-1.62 (m, 2H), 1.55 – 1.37 (m, 6H), 1.30 (dq, *J* = 14.3, 7.1 Hz, 2H), 0.88 (t, *J* = 7.1 Hz, 3H).

1-Butylazepane

1-Butylazepane was obtained as a colourless liquid after distillation in vacuo (67-69 °C @ 10.0mbar), 9.48 g, 61% yield. ^{1}H NMR (300 MHz, $CDCl_3$): *δ* 2.70 – 2.55 (m, 4H), 2.52 – 2.37 (m, 2H), 1.60 (brs, 8H), 1.52 – 1.37 (m, 2H), 1.30 (dq, *J* = 14.5, 7.1 Hz, 2H), 0.91 (t, *J* = 7.2 Hz, 3H).

1-(2-Methoxyethyl)pyrrolidine, $Pyrr_{2o1}$

1-(2-Methoxyethyl)pyrrolidine was obtained as a colourless liquid after distillation in vacuo (47-49 °C @ 10.1 mbar), 8.91 g, 69% yield. ^{1}H NMR (300 MHz, $CDCl_3$): *δ* 3.51 (t, *J* = 5.7 Hz, 2H), 3.37 (s, 3H), 2.67 (t, *J* = 5.7 Hz, 2H), 2.54 (s, 4H), 1.79 (s, 4H).

1-(2-Methoxyethyl)piperidine, Pip_{2o1}

1-(2-Methoxyethyl)piperidine was obtained as a colourless liquid after distillation in vacuo (58-60 °C @ 9.9mbar), 9.16 g, 64% yield. ^{1}H NMR (300 MHz, $CDCl_3$): *δ* 3.50 (dd, *J* = 11.9, 6.1 Hz, 2H), 3.35 (s, 3H), 2.52 (dd, *J* = 13.2, 7.4 Hz, 2H), 2.41 (s, 4H), 1.60 (dt, *J* = 10.5, 5.3 Hz, 4H), 1.43 (d, *J* = 5.0 Hz, 2H)

1-(2-Methoxyethyl)azepane, Aze_{2o1}

1-(2-Methoxyethyl)azepane was obtained as a colourless liquid after distillation in vacuo (103-105°C @ 4.3mbar) ^{1}H NMR (300 MHz, $CDCl_3$): *δ* 3.36 (s, 3H, OCH_3), 3.48 (t, *J* = 5.3 Hz 2H), 2.72 (m, 4H), 1.65 (m, 4H), 1.58 (m, 4H)

1-[2-(2-Methoxyethoxy)ethyl]pyrrolidine, $Pyrr_{2o2o1}$

1-[2-(2-Methoxyethoxy)ethyl]pyrrolidine was obtained as a colourless liquid after distillation in vacuo (118-120 °C @ 3.8mbar); ^{1}H NMR (300 MHz, $CDCl_3$): *δ* 3.62 (t, *J* = 5.3 Hz, 4H), 3.56 (d, *J* = 5.4 Hz, 2H), 3.39 (s, 3H), 2.67 (dd, *J* = 25.9, 19.7 Hz, 2H), 2.55 (brs, 4H), 1.78 (brs, 4H).

1-[2-(2-Methoxyethoxy)ethyl]piperidine, Pip_{2o2o1}

1-[2-(2-Methoxyethoxy)ethyl]piperidine was obtained as a colourless liquid after distillation in vacuo (137-141 °C @ 3.8mbar); ^{1}H NMR (300 MHz, $CDCl_3$): *δ* 3.70 – 3.57 (m, 4H), 3.54 (dd, *J* = 3.5, 1.7 Hz, 2H), 3.38 (s, 3H), 2.57 (t, *J* = 6.3 Hz, 2H), 2.43 (brs, 4H), 1.67 – 1.51 (m, 4H), 1.43 (d, *J* = 5.0 Hz, 2H)

1-[2-(2-Methoxyethoxy)ethyl]azepane, Aze_{2o2o1}

1-[2-(2-Methoxyethoxy)ethyl]azepane was obtained as a colourless liquid after distillation in vacuo (51-53 °C @ 0.2mbar); ^{1}H NMR (300 MHz, $CDCl_3$): *δ* 3.66 (m, 2H), 3.59 (m, 2H), 3.56 (m, 2H,), 3.37 (s, 3H,), 2.75 (t, *J*= 6.6 Hz, 2H), 2.70 (m, 4H,), 1.66 (m, 4H,), 1.58 (m, 4H,),

Synthesis of *N*-methyl-*N*-alkyl functionalised cyclic ammonium $[TFSI]^-$ ionic liquids

Under an inert atmosphere, dimethylsulfate (0.055 mol) was added dropwise to a stirred solution of alkylamine (0.05 mol) in toluene (50 cm^3) cooled in an ice-water bath. The mixture was left to reach room temperature and stirred vigorously overnight. After settling, the lower ionic liquid phase was collected and washed with further portions of toluene (2 x 10 cm^3). Volatiles were removed to yield the corresponding methylsulfate-based ILs, which were checked by ^{1}H-NMR. A solution of lithium bis{(trifluoromethyl)sulfonyl}imide (15.2 g, 0.053 mol) in water (50 cm^3) was mixed with a solution of *N*-alkyl-*N*-methylcyclic ammonium methylsulfate (0.05 mol) in dichloromethane (50 cm^3) in a 250 cm^3 round bottom flask and vigorously stirred for 24 h. The two phases were separated with a separating funnel. The organic phase containing the ionic liquid was the washed multiple times with water (10 x 10 cm^3). The solvent was then removed and the ionic liquid was then dried in vacuo at (60 °C, 0.02 mbar) for 72 h leaving an almost colourless liquid.

1-Butyl-1-methylpyrrolidinium methylsulfate, $[Pyrr_{14}][MeSO_4]$

^{1}H NMR (300 MHz, $CDCl_3$): *δ* 3.65 (s, 3H,), 3.62 (m, 4H), 3.43 (m, 2H), 3.12 (s, 3H), 2.23 (m, 4H), 1.72 (m, 2H), 1.39 (m 2H,) 0.95 (t, 3H, *J*= 7.4 Hz)

1-Butyl-1-methylpyrrolidinium bis{(trifluoromethyl)sulfonyl}imide, $[Pyrr_{14}][TFSI]$

^{1}H NMR (300 MHz, DMSO): δ 3.57 – 3.38 (m, 4H), 3.34 – 3.24 (m, 2H), 3.03 (s, 3H), 2.10 (s, 4H), 1.78 – 1.61 (m, 2H), 1.42 – 1.25 (m, 2H), 0.95 (t, *J* = 7.3 Hz, 3H). ^{13}C NMR (75 MHz, DMSO): *δ* 126.26 (s), 122.00 (s), 117.73 (s), 113.46 (s), 63.78 (s), 63.28 (s), 47.85 (s), 25.29 (s), 21.43 (s), 19.67 (s), 13.81 (s). CHNS calc.: C, 31.28%; H, 4.77%; N, 6.63%; S, 15.18%; found: C, 31.69%; H, 5.15%; N, 6.28%, S, 15.01%. Li content 23 ppm

1-Butyl-1-methylpiperidinium methylsulfate $[Pip_{14}][MeSO_4]$

^{1}H NMR (300 MHz, $CDCl_3$): δ 3.73 (dd, *J* = 14.7, 8.3 Hz, 4H), 3.45 (s, 3H), 3.34 (s, 3H), 1.91 (t, *J* = 11.5 Hz, 4H), 1.87 – 1.66 (m, 4H), 1.57 – 1.37 (m, 2H), 1.21 (dd, *J* = 11.7, 4.7 Hz, 2H), 1.01 (t, *J* = 7.3 Hz, 3H)

1-Butyl-1-methylpiperidinium bis{(trifluoromethyl)sulfonyl}imide, [Pip_{14}][TFSI]

^{1}H NMR (300 MHz, DMSO): δ 3.39 – 3.23 (m, 6H), 3.00 (s, 3H), 1.82 (brs, 4H), 1.71 – 1.52 (m, 4H), 1.39 – 1.27 (m, 2H), 0.96 (t, J = 7.3 Hz, 3H). ^{13}C NMR (75 MHz, DMSO): δ 126.25 (s), 121.99 (s), 117.73 (s), 113.48 (s), 62.65 (s), 60.36 (s), 47.33 (s), 23.31 (s), 21.04 (s), 19.61 (s), 13.77 (s). CHNS calc.: C, 33.02%; H, 5.08%; N, 6.42%; S, 14.69%; found: C, 33.01%; H, 4.93%; N, 6.42%; S, 14.69%. Li content 36 ppm

1-Butyl-1-methylazepinium methylsulfate, [Aze_{14}][$MeSO_4$]

^{1}H NMR (300 MHz, $CDCl_3$): δ 3.52 (m, 4H), 3.29 (s, 3H), 3.00 (s, 3H), 1.99 (m, 4H), 1.80 (m, 4H), 1.61 (m, 4H), 1.48 – 1.30 (m, 2H), 0.98 (t, J = 7.3 Hz, 3H)

1-Butyl-1-methylazepinium bis{(trifluoromethyl)sulfonyl}imide, [Aze_{14}][TFSI]

^{1}H NMR (300 MHz, DMSO): δ 3.55 – 3.34 (m, 4H), 3.29 (dd, J = 16.5, 8.1 Hz, 2H), 3.03 (s, 3H), 1.84 (s, 4H), 1.76 – 1.64 (m, 2H), 1.61 (s, 4H), 1.33 (dq, J = 14.4, 7.2 Hz, 2H), 0.96 (t, J = 7.3 Hz, 3H). ^{13}C NMR (75 MHz, $CDCl_3$): δ 126.49 (s), 122.38 (s), 118.12 (s), 113.96 (s), 65.90 (s), 65.17 (s), 50.85 (s), 27.85 (s), 24.83 (s), 22.13 (s), 19.90 (s), 13.74 (s). CHNS calc.: C, 34.68%; H, 5.37%; N, 6.22%; S, 14.20%; found: C, 34.28; H, 5.18%; N, 6.17%; S, 13.70%. Li content 14 ppm.

1-(2-Methoxyethyl)-1-methylpyrrolidinium methylsulfate, [$Pyrr_{1(2o1)}$][$MeSO_4$]

^{1}H NMR (300 MHz, DMSO): δ 3.99 (s, 2H), 3.73 (dd, J = 12.4, 7.6 Hz, 2H), 3.33(s, 3H), 3.35 (m, 4H), 3.26 (s, 3H), 3.00 (s, 3H), 1.88 (s, 4H),

1-(2-Methoxyethyl)-1-methylpyrrolidinium bis{(trifluoromethyl)sulfonyl}imide, [$Pyrr_{1(2o1)}$][TFSI]

^{1}H-NMR 300 MHz, DMSO): δ 3.78 (m, 2H), 3.62 – 3.40 (m, 6H), 3.30 (s, 3H), 3.05 (s, 3H), 2.09 (brs, 4H). ^{13}C NMR (75 MHz, DMSO): δ 126.26 (s), 122.00 (s),117.73 (s), 113.46 (s), 66.36 (s), 64.53 (s), 62.43 (s), 58.42 (s), 48.37 (s), 21.20 (s). CHNS cal.: C, 28.30%; H, 4.27%; N, 6.60%; S, 15.11%; found: C, 27.99%; H, 5.15%; N, 6.48%; S, 15.03%. Li content 33 ppm.

1-(2-Methoxyethyl)-1-methylpiperidinium methylsulfate, [$Pip_{1(2o1)}$][$MeSO_4$]

^{1}H NMR (300 MHz, DMSO): δ 3.75 (s, 2H), 3.57 (dd, J = 12.6, 7.9 Hz, 2H), 3.38(s, 3H), 3.35 (m, 4H), 3.30 (s, 3H), 3.05 (s, 3H), 1.78 (s, 4H), 1.63 – 1.41 (m, 2H).

1-(2-Methoxyethyl)-1-methylpiperidinium bis{(trifluoromethyl)sulfonyl}imide, [$Pip_{1(2o1)}$][TFSI]

^{1}H NMR (300 MHz, $CDCl_3$): δ 3.95 (d, J= 4.2 Hz, 2H), 3.79–3.72 (m, 4H), 3.70–3.60(m, 2H), 3.30 (s, 3H), 3.28 (s, 3H), 1.78 (brs, 4H), 1.73 – 1.65 (m, 2H).^{13}C NMR (75 MHz, $CDCl_3$): δ 126.16 (s),121.89 (s), 117.48 (s), 113.26 (s) 65.39 (s), 62.07 (s), 61.20 (s), 58.48 (s), 48.33 (s), 20.93 (s), 19.66 (s). CHNS calc.; C, 30.14%; H, 4.60%; N, 6.39%; S, 14.63%; found: C, 30.62%; H, 5.01%; N, 6.38%, S, 14.31%. Li content 44 ppm

1-(2-Methoxyethyl)-1-methylazepanium methylsulfate, [$Aze_{1(2o1)}$][$MeSO_4$]

^{1}H NMR (300 MHz, DMSO): δ 3.76 (s, 2H), 3.60 – 3.46 (m, 4H), 3.46 – 3.35 (m, 5H), 3.31 (s, 3H), 3.05 (s, 3H), 1.82 (s, 4H), 1.59 (s, 4H)

1-(2-Methoxyethyl)-1-methylazepanium bis{(trifluoromethyl)sulfonyl}imide, [$Aze_{1(2o1)}$][TFSI]

^{1}H NMR (300 MHz, DMSO): δ 3.79 (m, 2H), 3.55 – 3.44 (m, 4H), 3.37 (m, 2H), 3.30 (s, 3H), 3.03 (s, 3H), 1.79 (m, 4H), 1.57 (m, 4H). ^{13}C NMR (75 MHz, DMSO): δ 126.26 (s), 122.00 (s), 117.73 (s), 113.47 (s), 65.87 (s), 64.50 (s), 63.63 (s), 58.37 (s), 51.20 (s), 27.36 (s), 21.17 (s). CHNS calc.: C, 31.86%; H, 4.90%; N, 6.19%; S, 14.17%; found: C, 32.06%; H, 4.35%; N, 6.33%; S, 13.72%. Li content 59 ppm.

1-[2-(2-Methoxyethoxy)ethyl]-1-methylpyrrolidinium methylsulfate, [$Pyrr_{1(2o2o1)}$][$MeSO_4$]

^{1}H NMR (300 MHz, DMSO): δ 3.79 (s, 2H), 3.64 – 3.52 (m, 4H), 3.55 – 3.43 (m, 6H), 3.36 (s, 3H), 3.26 (s, 3H), 3.03 (s, 3H), 2.08 (s, 4H).

1-[2-(2-Methoxyethoxy)ethyl]-1-methylpyrrolidinium bis{(trifluoromethyl)sulfonyl}imide, [$Pyrr_{1(2o2o1)}$][TFSI]

^{1}H NMR (300 MHz, DMSO): δ 3.81 (m, 2H), 3.63 (m, 6H), 3.49 (m, 4H), 3.32 (s, 3H), 3.09 (s, 3H), 2.18 (m, 4H). ^{13}C NMR (75 MHz, DMSO): δ 126.27 (s), 122.00 (s), 117.74 (s), 113.47 (s), 71.45 (s), 69.57 (s), 64.86 (s), 64.52 (s), 62.51 (s), 58.13 (s), 48.41 (s), 22.73. CHNS calc.: C, 30.78%; H, 4.74%; N, 5.98%; S, 13.66%; found: C, 31.06%; H, 4.85%; N, 5.93%; S, 13.72%. Li content 43 ppm

1-[2-(2-Methoxyethoxy)ethyl]-1-methylpiperidinium methylsulfate, [$Pip_{1(2o2o1)}$][$MeSO_4$]

^{1}H NMR (300 MHz, $CDCl_3$): δ 3.84 (s, 2H), 3.58 (dd, *J* = 5.4, 3.4 Hz, 4H), 3.50 – 3.41 (m, 2H), 3.42 – 3.28 (m, 7H), 3.25 (s, 3H), 3.06 (s, 3H), 1.78 (d, *J* = 5.1 Hz, 4H), 1.52 (m, 2H)

1-[2-(2-Methoxyethoxy)ethyl]-1-methylpiperidinium bis{(trifluoromethyl)sulfonyl}imide, [$Pip_{1(2o2o1)}$][TFSI]

^{1}H NMR (300 MHz, DMSO): δ 3.87 (s, 2H), 3.58 (t, *J* = 10.5 Hz, 4H), 3.48 (dd, *J* = 10.9, 7.3 Hz, 2H), 3.46 – 3.29 (m, 4H), 3.28 (s, 3H), 3.09 (s, 3H), 1.94 – 1.72 (m, 4H), 1.57 (d, *J* = 4.6 Hz, 2H). ^{13}C NMR (75 MHz, DMSO): δ 126.27 (s), 122.00 (s), 117.74 (s), 113.47 (s), 71.33 (s), 69.72 (s), 63.87 (s), 61.70 (d, *J* = 47.4 Hz), 61.25 (s), 58.32 (s), 48.48 (s), 20.89 (s), 19.66 (s). CHNS calc. C, 32.36%; H, 5.01%; N, 5.81%; S, 13.29%; found: C, 31.84%; H, 5.45%; N, 5.42%; S, 12.82%. Li content 59 ppm

1-[2-(2-Methoxyethoxy)ethyl]-1-methylazepanium methylsulfate, [$Aze_{1(2o2o1)}$][$MeSO_4$]

^{1}H NMR (300 MHz, DMSO): δ 3.84 (s, 2H), 3.64 – 3.49 (m, 6H), 3.46 (dd, *J* = 5.7, 3.3 Hz, 2H), 3.44 – 3.31 (m, 5H), 3.25 (s, 3H), 3.03 (d, *J* = 10.5 Hz, 3H), 1.81 (s, 4H), 1.59 (d, *J* = 5.1 Hz, 4H).

1-[2-(2-Methoxyethoxy)ethyl]-1-methylazepanium bis{(trifluoromethyl)sulfonyl}imide, [$Aze_{1(2o2o1)}$][TFSI]

^{1}H NMR (300 MHz, DMSO): δ 3.84 (s, 2H), 3.63 – 3.54 (m, 2H), 3.51 (dd, *J* = 8.5, 4.2 Hz, 2H), 3.49 – 3.42 (m, 2H), 3.42 – 3.28 (m, 4H), 3.28 (s, 3H) 3.05 (s, 3H), 1.81 (s, 4H), 1.58 (s, 4H). ^{13}C NMR (75 MHz, DMSO): δ

126.26 (s), 121.99 (s), 117.73 (s), 113.46 (s), 71.35 (s), 69.72 (s), 64.41 (s), 63.65 (s), 58.41 (s), 51.37 (s), 27.57 (s), 21.22 (s). CHNS calc.: C, 33.87%; H, 5.28%; N, 5.64%; S, 12.92%; found: C, 34.12%; H, 5.04%; N, 5.95%; S, 13.3%.

Synthesis of diether functionalised ammonium $[TFSI]^-$ ionic liquids

Under an inert atmosphere, the appropriate bromoether (2-bromoethyl methyl ether or 1-bromo-2-(2-methoxyethoxy)ethane, 0.055 mol) was added dropwise to a stirred solution of monoether tertiary amine (0.05 mol) in acetonitrile (50 cm^3). The mixture was stirred vigorously overnight under reflux conditions. Volatiles were removed to yield the corresponding crude diether functionalized ammonium bromide ILs which were verified by ^{1}H-NMR. A solution of lithium bis{(trifluoromethyl)sulfonyl}imide (15.2 g, 0.053 mol) in water (50 cm^3) was mixed with a solution of diether cyclicammonium bromide (0.04 mol) in dichloromethane (50 cm^3) in a 250 cm^3 round bottom flask and vigorously stirred. The two phases were separated with a separating funnel. The organic phase containing the ionic liquid was the washed multiple times with water (10 x 10 cm^3). The solvent was then removed and the ionic liquid was then dried in vacuo at (60 °C, 0.02 mbar) for 72 h leaving an almost colourless liquid.

1,1-Di(2-methoxyethyl)-pyrrolidinium bromide, $[Pyrr_{(2o1)2}]Br$

^{1}H NMR (300 MHz, DMSO): δ 3.70 (t, *J* = 10.4 Hz, 4H), 3.67 – 3.57 (m, 4H), 3.56 – 3.46 (m, 4H), 3.28 (s, 6H), 1.82 (s, 4H).

1,1-Di(2-methoxyethyl)pyrrolidinium bis{(trifluoromethyl)sulfonyl}imide, $[Pyrr_{(2o1)2}][TFSI]$

^{1}H NMR (300 MHz, DMSO): δ 3.73 (s, 4H), 3.68 – 3.50 (m, 8H), 3.33 (s, 6H), 2.07 (s, 4H). ^{13}C NMR (75 MHz, DMSO): δ 126.26 (s), 122.00 (s), 117.73 (s), 113.33 (s), 66.31 (s), 63.46 (s), 58.99 (s), 58.54 (s), 21.26 (s). CHNS calc.: C, 30.77%; H, 4.73%; N, 5.98%; S, 13.69%; found: C, 30.82%; H, 5.06%; N, 5.63%; S, 12.86%. Li content 86 ppm

1,1-Di(2-methoxyethyl)piperidinium bromide, $[Pip_{(2o1)2}]Br$

^{1}H NMR (300 MHz, DMSO): δ 3.78 (t, *J* = 11.0 Hz, 4H), 3.66 – 3.52 (m, 12H), 3.51 – 3.40 (m, 4H), 3.25 (s, 6H), 2.03 (s, 4H).

1,1-Di(2-methoxyethyl)piperidinium bis{(trifluoromethyl)sulfonyl}imide, $[Pip_{(2o1)2}][TFSI]$

^{1}H NMR (300 MHz, DMSO): δ 3.82 (t, *J* = 14.5 Hz, 2H), 3.65 – 3.53 (m, 4H), 3.53 – 3.44 (m, 2H), 3.36 (dd, *J* = 11.4, 6.8 Hz, 4H), 3.27 (s, 3H), 3.08 (s, 3H), 1.92 – 1.70 (m, 4H), 1.66 – 1.44 (m, 2H). ^{13}C NMR (75 MHz, DMSO): δ 126.16 (s), 122.00 (s), 117.73 (s), 113.46 (s), 71.34 (s), 69.72 (s), 63.88 (s), 62.02 (s), 61.23 (s), 58.37 (s), 48.48 (s), 20.92 (s), 19.67 (s). CHNS calc.: C, 32.26%; H, 5.01%; N, 5.81%; S, 13.29%; found: C, 32.82%; H, 4.89%; N, 5.47%; S, 13.06%. Li content 23ppm

1,1-Di(2-methoxyethyl)azepanium bromide, [$Aze_{(2o1)2}$]Br

^{1}H NMR (300 MHz, DMSO): δ 4.13 (d, J = 3.7 Hz, 4H), 4.00 (t, J = 10.2 Hz, 4H), 3.98 – 3.86 (m, 4H), 3.70 (s, 6H) 2.24 (s, 4H), 2.00 (s, 4H)

1,1-Di(2-methoxyethyl)azepanium bis{(trifluoromethyl)sulfonyl}imide, [$Aze_{(2o1)2}$][TFSI]

^{1}H NMR (300 MHz, DMSO): δ 3.71 (d, *J* = 3.8 Hz, 4H), 3.58 (dd, *J* = 12.0, 8.0 Hz, 4H), 3.50 (m, 4H), 3.28 (s, 6H), 1.82 (s, 4H), 1.58 (s, 4H). ^{13}C NMR (75 MHz, DMSO): δ 126.26 (s), 121.99 (s), 117.73 (s), 113.47 (s), 65.89 (s), 63.20 (s), 61.27 (s), 58.55 (s), 27.75 (s), 21.24 (s). CHNS calc.: C, 34.01%; H, 4.89%; N, 5.67%; S, 12.97%; found: C, 34.72%; H, 5.16%; N, 5.13%; S, 12.43%. Li content 32 ppm

1,1-Di[2-(2-methoxyethoxy)ethyl]pyrrolidinium bromide, [$Pyrr_{(2o2o1)2}$]Br

^{1}H NMR (300 MHz, DMSO): δ 3.87 – 3.72 (m, 4H), 3.67 – 3.50 (m, 12H), 3.50 – 3.41 (m, 4H), 3.25 (s, 6H), 1.82 (s, 4H)

1,1-Di[2-(2-methoxyethoxy)ethyl]piperidinium bis{(trifluoromethyl)sulfonyl}imide, [$Pyrr_{(2o2o1)2}$][TFSI]

^{1}H NMR (300 MHz, DMSO): δ 3.65 – 3.56 (m, 4H), 3.43 – 3.32 (m, 12H), 3.29 – 3.24 (m, 4H), 3.06 (s, 6H) 1.87 (brs, 4H). ^{13}C NMR (75 MHz, DMSO): δ 126.65 (s), 122.39 (s), 118.13 (s), 113.87 (s), 78.70 (s), 77.74 (s), 77.30 (s), 71.87 (s), 70.72 (s), 64.64 (s), 61.68 (s), 59.94 (s), 59.21 (s), 53.90 (s), 21.08 (s), 20.26 (s). CHNS calc.: C, 34.53%; H, 5.43%; N, 5.03%; S, 11.52%; found: C, 33.92%; H, 4.96%; N, 4.63%; S, 10.99%; Li content 71 ppm

1,1-Di[2-(2-methoxyethoxy)ethyl]piperidinium bromide, [$Pip_{(2o2o1)2}$]Br

^{1}H NMR (300 MHz, $CDCl_3$): δ 3.88 (s, 4H), 3.80 (dd, *J* = 16.1, 10.8 Hz, 4H), 3.75 – 3.59 (m, 4H), 3.61 – 3.47 (m, 8H), 3.56 (s, 6H), 1.90 (s, 4H), 1.60 (s, 2H)

1,1-Di[2-(2-methoxyethoxy)ethyl]piperidinium bis{(trifluoromethyl)sulfonyl}imide, [$Pip_{(2o2o1)2}$][TFSI]

^{1}H NMR (300 MHz, DMSO): δ 3.79 (s, 4H), 3.70 – 3.64 (m, 4H), 3.64 – 3.52 (m, 4H), 3.52 – 3.38 (m, 8H), 3.27 (s, 6H), 1.76 (brs, 4H), 1.68 – 1.50 (m, 2H). ^{13}C NMR (75 MHz, DMSO): δ 126.47 (s), 122.00 (s), 117.73 (s), 113.46 (s), 71.36 (s), 69.74 (s), 63.92 (s), 60.16 (s), 58.70 (s), 20.75 (s), 19.57 (s). CHNS calc.: C, 35.54%; H, 6.32%; N, 4.88%; S, 11.16%; found: C, 35.61%; H, 5.74%; N, 4.90%; S, 10.73%. Li content 45ppm

1,1-Di[2-(2-methoxyethoxy)ethyl]azepanium bromide, [$Aze_{(2o2o1)2}$]Br

1H NMR (300 MHz, DMSO): δ 3.87 – 3.72 (m, 4H), 3.67 – 3.50 (m, 8H), 3.50 – 3.41 (m, 8H), 3.25 (s, 6H), 1.88 (s, 4H), 1.60 (s, 4H).

1,1-Di[2-(2-methoxyethoxy)ethyl]azepanium bis{(trifluoromethyl)sulfonyl}imide, [$Aze_{(2o2o1)2}$][TFSI]

^{1}H NMR (300 MHz, $CDCl_3$): δ 3.77 (d, *J* = 18.2 Hz, 4H), 3.66 – 3.57 (m, 6H), 3.57 – 3.50 (m, 6H), 3.50 – 3.41 (m, 4H), 3.26 (s, 6H), 1.82 (s, 4H), 1.58 (s, 4H). ^{13}C NMR (75 MHz, $CDCl_3$): δ 126.67 (s), 122.41 (s), 118.15 (s), 113.89 (s), 71.85 (s), 70.74 (s), 65.13 (s), 64.72 (s), 62.92 (s), 59.24 (s), 28.33 (s), 26.84 (s), 22.13 (s), CHNS calc.: C, 36.73%; H, 6.51%; N, 4.76%; S, 10.90%; found: C, 36.61%; H, 5.84%; N, 4.75%; S, 10.14%. Li content 26 ppm

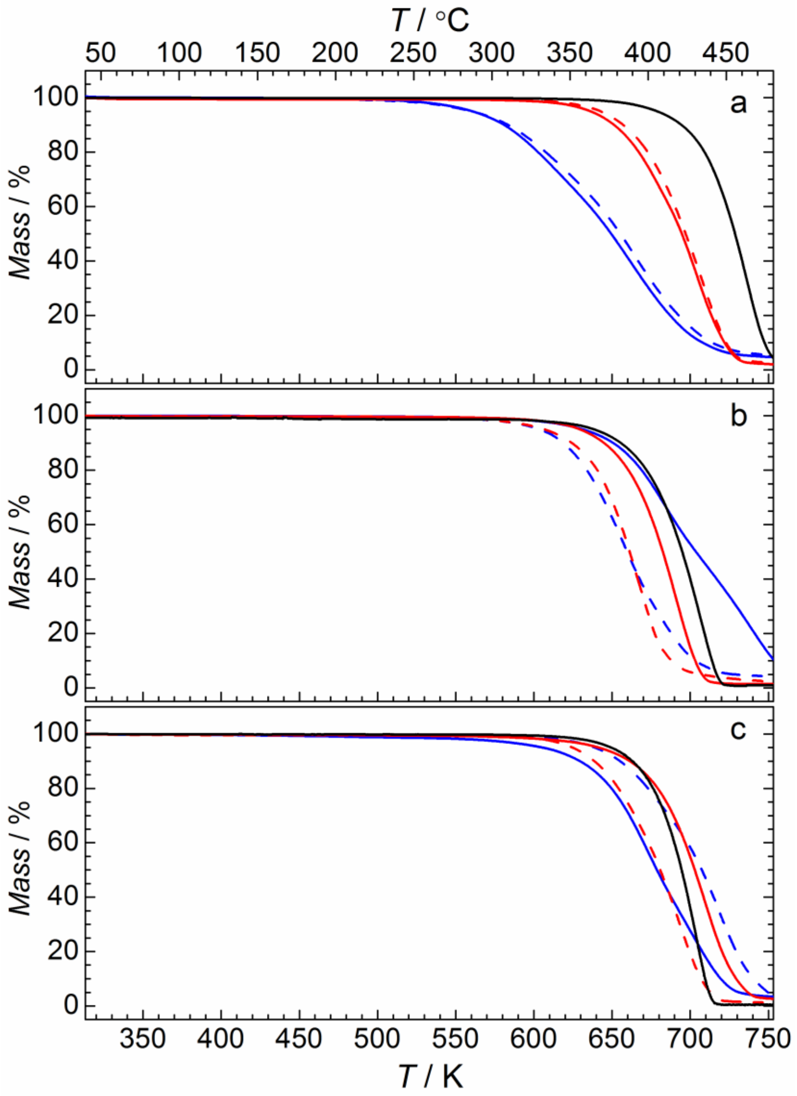

Figure S 1. Thermogravimetric analysis (TGA) curves of the (a) pyrrolidinium, (b) piperidinium and (c) azepanium-based [TFSI]$^-$-based ILs. Line styles represent the represent the functional groups of a given cyclic alkylammonium cation; — = 14, — = 1(2o1), - - - = 1(2o2o1), — = $(2o1)_2$, - - - = $(2o2o1)_2$.

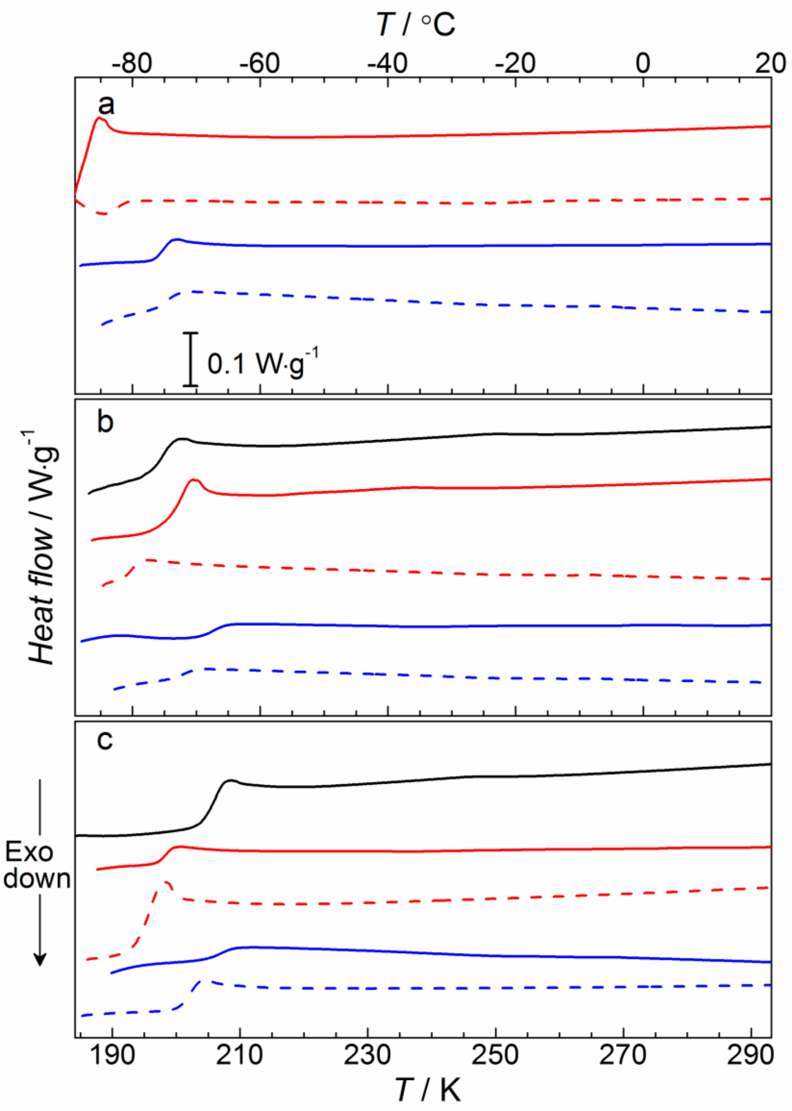

Figure S 2. Heating traces of the DSC thermograms of the (a) pyrrolidinium, (b) piperidinium and (c) azepanium-based [TFSI]⁻-based ILs. Line styles represent the represent the functional groups of a given cyclic alkylammonium cation; — = 14, — = 1(2o1), - - -= 1(2o2o1), — = $(2o1)_2$, - - - = $(2o2o1)_2$.

Table S 1. Experimental density, *ρ*, as a function of temperature of the pyrrolidinium, piperidinium and azepanium-based $[TFSI]^-$-based ILs. Column titles highlight the cation abbreviation of the respective $[TFSI]^-$ IL.

***T* / K**	***ρ* / g·cm⁻³**				
	$[Pyrr_{14}]^+$	$[Pyrr_{1(2o1)}]^+$	$[Pyrr_{1(2o2o1)}]^+$	$[Pyrr_{(2o1)2}]^+$	$[Pyrr_{(2o2o1)2}]^+$
293.15	1.4096	1.4594	1.4183	1.4354	1.4057
298.15	1.4054	1.4547	1.4137	1.4308	1.4010
303.15	1.4009	1.4499	1.4090	1.4260	1.3962
313.15	1.3917	1.4403	1.3996	1.4163	1.3867
323.15	1.3827	1.4309	1.3929	1.4069	1.3774
333.15	1.3738	1.4216	1.3815	1.3976	1.3682
343.15	1.3650	1.4134	1.3724	1.3884	1.3591
353.15	1.3562	1.4045	1.3635	1.3792	1.3501
363.15	1.3475	1.3953	1.3546	1.3702	1.3411
***T* / K**	***ρ* / g·cm⁻³**				
	$[Pip_{14}]^+$	$[Pip_{1(2o1)}]^+$	$[Pip_{1(2o2o1)}]^+$	$[Pip_{(2o1)2}]^+$	$[Pip_{(2o2o1)2}]^+$
293.15	1.3819	1.4588	1.4001	1.4135	1.3576
298.15	1.3775		1.3956	1.4087	1.3531
303.15	1.3730	1.4500	1.3910	1.4040	1.3488
313.15	1.3641	1.4403	1.3818	1.3945	1.3399
323.15	1.3554	1.4309	1.3727	1.3853	1.3312
333.15	1.3467	1.4216	1.3638	1.3759	1.3227
343.15	1.3382	1.4123	1.3549	1.3667	1.3142
353.15	1.3297	1.4031	1.3461	1.3575	1.3057
363.15	1.3213	1.394	1.3373	1.3482	1.2971
***T* / K**	***ρ* / g·cm⁻³**				
	$[Aze_{14}]^+$	$[Aze_{1(2o1)}]^+$	$[Aze_{1(2o2o1)}]^+$	$[Aze_{(2o1)2}]^+$	$[Aze_{(2o2o1)2}]^+$
293.15	1.3773	1.4226	1.3992	1.3438	1.3572
298.15	1.3729	1.4179	1.3949	1.3393	1.3527
303.15	1.3685	1.4134	1.3902	1.3349	1.3483
313.15	1.3597	1.4042	1.3808	1.3258	1.3393
323.15	1.3510	1.3952	1.3715	1.3170	1.3306
333.15	1.3425	1.3863	1.3617	1.3075	1.3220
343.15	1.3341	1.3775	1.3516	1.2986	1.3135
353.15	1.3257	1.3686	1.3408	1.2896	1.3050
363.15	1.3173	1.3598	1.3275	1.2805	1.2966

Table S 2. Isobaric coefficients of thermal expansion, α_p, of the pyrrolidinium, piperidinium and azepanium-based $[TFSI]^-$-based ILs. Column titles highlight the cation abbreviation of the respective $[TFSI]^-$ IL. Calculated from the experimental density using Equation 2 in the main text.

***T* / K**	$10^4 \cdot \alpha_p$ / K^{-1}				
	$[Pyrr_{14}]^+$	$[Pyrr_{1(2o1)}]^+$	$[Pyrr_{1(2o2o1)}]^+$	$[Pyrr_{(2o1)2}]^+$	$[Pyrr_{(2o2o1)2}]^+$
293.15	5.98	6.46	6.51	6.43	6.71
298.15	6.19	6.53	6.58	6.57	6.78
303.15	6.51	6.63	6.68	6.78	6.85
313.15	6.54	6.60	5.74	6.74	6.78
323.15	6.47	6.53	6.52	6.65	6.72
333.15	6.44	6.15	7.41	6.62	6.69
343.15	6.45	6.05	6.56	6.63	6.66
353.15	6.45	6.45	6.53	6.60	6.67
363.15	6.41	6.55	6.53	6.53	6.67
***T* / K**	$10^4 \cdot \alpha_p$ / K^{-1}				
	$[Pip_{14}]^+$	$[Pip_{1(2o1)}]^+$	$[Pip_{1(2o2o1)}]^+$	$[Pip_{(2o1)2}]^+$	$[Pip_{(2o2o1)2}]^+$
293.15	6.39	6.07	6.45	6.81	6.65
298.15	6.46		6.52	6.74	6.50
303.15	6.53	6.38	6.63	6.74	6.50
313.15	6.45	6.63	6.62	6.70	6.57
323.15	6.42	6.53	6.56	6.71	6.46
333.15	6.39	6.54	6.53	6.76	6.43
343.15	6.35	6.55	6.53	6.73	6.47
353.15	6.35	6.52	6.54	6.81	6.55
363.15	6.32	6.49	6.54	6.85	6.59
***T* / K**	$10^4 \cdot \alpha_p$ / K^{-1}				
	$[Aze_{14}]^+$	$[Aze_{1(2o1)}]^+$	$[Aze_{1(2o2o1)}]^+$	$[Aze_{(2o1)2}]^+$	$[Aze_{(2o2o1)2}]^+$
293.15	6.41	6.63	6.17	6.72	6.65
298.15	6.41	6.49	6.45	6.64	6.58
303.15	6.44	6.45	6.77	6.72	6.61
313.15	6.44	6.48	6.77	6.75	6.61
323.15	6.36	6.41	6.97	6.95	6.50
333.15	6.29	6.38	7.31	7.03	6.47
343.15	6.30	6.43	7.74	6.89	6.47
353.15	6.34	6.47	9.00	7.02	6.47
363.15	6.34	6.43	9.92	7.06	6.44

Table S 3. Experimental viscosity, *η*, as a function of temperature of the pyrrolidinium, piperidinium and azepanium-based $[TFSI]^-$-based ILs. Column titles highlight the cation abbreviation of the respective $[TFSI]^-$ IL.

***T* / K**	***η* / mPa·s**	***T* / K**	***η* / mPa·s**	***T* / K**	***η* / mPa·s**	***T* / K**	***η* / mPa·s**	***T* / K**	***η* / mPa·s**
$[Pyrr_{14}]^+$		$[Pyrr_{1(2o1)}]^+$		$[Pyrr_{1(2o2o1)}]^+$		$[Pyrr_{(2o1)2}]^+$		$[Pyrr_{(2o2o1)2}]^+$	
291.15	108.00	290.15	71.05	290.15	74.72	291.15	109.00	292.05	197.80
297.15	80.85	295.85	54.44	295.85	56.93	297.15	78.42	298.05	124.70
303.05	62.13	301.45	42.78	301.45	44.25	303.15	58.76	304.05	84.30
309.05	48.68	307.15	34.40	307.15	35.25	309.05	45.03	309.95	62.39
314.95	38.95	312.75	28.14	312.75	28.73	314.95	34.98	315.85	45.72
320.85	31.69	318.35	23.41	318.35	23.74	320.85	27.81	321.75	35.56
326.75	26.22	323.95	19.76	323.95	20.01	326.75	22.59	327.75	28.62
332.65	21.95	329.65	16.91	329.65	17.03	332.75	18.67	333.65	23.39
338.65	18.66	335.15	14.62	335.25	14.69	338.65	15.65	339.55	19.43
344.55	15.93	340.85	12.74	340.85	12.74	344.55	13.26	345.45	16.25
350.45	13.74	346.35	11.22	346.45	11.14	350.45	11.34	351.35	13.72
356.35	11.95	352.05	9.93	352.05	9.74	356.45	9.79	357.25	11.75
362.35	10.53	357.55	8.85	357.55	8.74	362.35	8.52		

***T* / K**	***η* / mPa·s**	***T* / K**	***η* / mPa·s**	***T* / K**	***η* / mPa·s**	***T* / K**	***η* / mPa·s**	***T* / K**	***η* / mPa·s**
$[Pip_{14}]^+$		$[Pip_{1(2o1)}]^+$		$[Pip_{1(2o2o1)}]^+$		$[Pip_{(2o1)2}]^+$		$[Pip_{(2o2o1)2}]^+$	
290.15	283.30	291.05	156.20	290.15	127.70	292.05	167.10	292.15	199.20
295.85	197.70	297.05	111.30	295.75	94.90	298.05	109.30	298.35	135.90
301.45	141.40	302.95	81.63	301.45	71.82	303.95	75.00	304.75	95.90
307.05	104.00	308.85	62.00	307.05	55.29	309.95	53.58	310.95	70.04
312.65	78.93	314.85	48.05	312.75	43.67	315.85	39.98	317.15	51.99
318.25	61.32	320.75	38.19	318.35	34.85	321.75	30.78	323.35	39.61
323.85	48.53	326.65	30.77	323.95	28.48	327.75	24.49	329.55	30.95
329.55	38.97	332.55	25.31	329.55	23.54	333.65	19.85	335.85	24.86
335.15	31.89	338.65	21.20	335.15	19.91	339.55	16.32	342.15	20.70
340.75	26.47	344.45	17.80	340.75	17.07	345.45	13.58	348.35	17.60
346.35	22.23	350.35	15.43	346.45	14.72	351.35	11.51	354.55	15.22
351.95	18.89	356.25	13.30	351.95	12.87	357.25	9.89	360.75	13.18
357.65	16.26	362.25	11.72	357.55	11.36			367.05	11.31
		368.05	10.19						

***T* / K**	***η* / mPa·s**	***T* / K**	***η* / mPa·s**	***T* / K**	***η* / mPa·s**	***T* / K**	***η* / mPa·s**	***T* / K**	***η* / mPa·s**
$[Aze_{14}]^+$		$[Aze_{1(2o1)}]^+$		$[Aze_{1(2o2o1)}]^+$		$[Aze_{(2o1)2}]^+$		$[Aze_{(2o2o1)2}]^+$	
290.15	561.30	290.15	221.80	292.15	185.00	292.05	206.50	289.55	395.30
295.95	361.70	295.95	154.70	298.35	125.90	298.05	136.90	294.95	264.30
301.55	243.60	301.55	111.70	304.65	89.44	304.05	95.23	300.25	183.10
307.15	170.70	307.15	82.89	310.85	65.90	309.95	68.33	305.55	131.30
312.75	124.00	312.75	63.33	317.15	49.85	315.85	51.02	310.85	97.02
318.35	92.44	318.35	49.58	323.35	38.45	321.75	39.23	316.15	73.76
323.95	70.83	323.95	39.71	329.65	30.28	327.65	31.10	321.35	57.65
329.65	55.15	329.65	32.21	335.85	24.33	333.65	25.12	326.75	45.96
335.25	43.97	335.25	26.52	342.05	19.96	339.55	20.61	332.05	37.11
340.85	35.50	340.85	22.40	348.35	16.65	345.45	17.07	337.35	30.31
346.35	29.05	346.35	18.92	354.55	14.18	351.35	14.32	342.55	25.00
352.05	24.21	352.05	16.18	360.75	12.24	357.35	12.20	347.95	21.39
357.65	20.29	357.65	14.04	366.95	10.64				

Table S 4. Experimental conductivity, *σ*, as a function of temperature of the pyrrolidinium, piperidinium and azepanium-based $[TFSI]^-$-based ILs. Column titles highlight the cation abbreviation of the respective $[TFSI]^-$ IL.

***T* / K**	***σ* / mS·cm^{-1}**	***T* / K**	***σ* / mS·cm^{-1}**	***T* / K**	***σ* / mS·cm^{-1}**	***T* / K**	***σ* / mS·cm^{-1}**	***T* / K**	***σ* / mS·cm^{-1}**
$[\text{Pyrr}_{14}]^+$		$[\text{Pyrr}_{1(2o1)}]^+$		$[\text{Pyrr}_{1(2o2o1)}]^+$		$[\text{Pyrr}_{(2o1)2}]^+$		$[\text{Pyrr}_{(2o2o1)2}]^+$	
293.35	2.20	292.85	2.99	293.75	2.34	291.65	1.19	293.15	0.96
296.05	2.49	293.65	3.08	299.35	2.93	293.75	1.33	298.75	1.27
299.25	2.85	296.15	3.40	304.85	3.61	298.95	1.83	303.95	1.64
301.65	3.14	298.75	3.75	308.15	4.06	304.05	2.32	313.55	2.45
304.95	3.58	304.35	4.64	313.15	4.79	313.35	3.42	322.45	3.36
309.25	4.19	313.45	6.27	323.55	6.51	323.05	4.87	333.05	4.67
313.15	4.81	321.55	7.95	332.35	8.18	332.85	6.61	342.55	6.03
319.95	6.00	325.05	8.75	343.75	10.65	342.95	8.58	353.95	7.92
323.15	6.60	333.15	10.72	352.95	12.86	354.25	11.16	363.35	9.65
333.45	8.80	343.45	13.62	363.15	15.60	363.75	13.63		
342.05	10.85	353.95	16.82						
353.55	13.98	364.35	20.20						
361.85	16.49								

***T* / K**	***σ* / mS·cm^{-1}**	***T* / K**	***σ* / mS·cm^{-1}**	***T* / K**	***σ* / mS·cm^{-1}**	***T* / K**	***σ* / mS·cm^{-1}**	***T* / K**	***σ* / mS·cm^{-1}**
$[\text{Pip}_{14}]^+$		$[\text{Pip}_{1(2o1)}]^+$		$[\text{Pip}_{1(2o2o1)}]^+$		$[\text{Pip}_{(2o1)2}]^+$		$[\text{Pip}_{(2o2o1)2}]^+$	
293.45	0.79	293.65	1.37	292.35	1.20	293.05	1.25	292.65	0.72
298.95	1.08	299.75	1.89	293.35	1.26	298.45	1.67	293.55	0.75
304.65	1.43	303.45	2.25	298.35	1.65	303.15	2.06	293.65	0.76
312.85	2.20	313.15	3.37	304.25	2.16	312.65	3.03	299.05	1.02
322.05	3.20	323.45	4.84	312.95	3.05	321.85	4.19	303.25	1.25
324.85	3.52	333.15	6.60	323.35	4.37	332.95	5.87	313.55	2.04
333.35	4.75	342.85	8.68	334.55	6.12	343.35	7.73	323.15	2.93
343.15	6.45	354.85	11.51	343.85	7.80	353.45	9.79	333.05	4.07
349.55	7.74	363.45	13.77	353.95	9.87	363.65	12.07	343.85	5.56
354.25	8.79			363.85	12.11			353.85	7.15
363.75	11.04							363.85	8.96

***T* / K**	***σ* / mS·cm^{-1}**	***T* / K**	***σ* / mS·cm^{-1}**	***T* / K**	***σ* / mS·cm^{-1}**	***T* / K**	***σ* / mS·cm^{-1}**	***T* / K**	***σ* / mS·cm^{-1}**
$[\text{Aze}_{14}]^+$		$[\text{Aze}_{1(2o1)}]^+$		$[\text{Aze}_{1(2o2o1)}]^+$		$[\text{Aze}_{(2o1)2}]^+$		$[\text{Aze}_{(2o2o1)2}]^+$	
293.85	0.45	293.45	0.84	293.55	0.84	293.15	0.52	293.75	0.42
299.05	0.64	296.45	1.00	299.35	1.16	298.45	0.71	298.75	0.58
302.95	0.81	298.75	1.12	303.55	1.44	304.25	0.97	303.75	0.77
307.45	1.05	303.15	1.40	314.45	2.39	314.25	1.59	312.65	1.21
313.45	1.43	314.15	2.50	325.15	3.59	323.85	2.34	323.35	1.98
323.35	2.35	323.05	3.60	334.25	4.82	332.65	3.27	333.45	2.89
333.15	3.47	333.85	5.22	343.45	6.29	343.15	4.54	343.75	4.03
344.65	5.09	344.05	7.09	355.15	8.45	353.85	6.06	353.95	5.41
355.25	7.00	354.95	9.36	362.95	10.06	363.65	7.68	363.85	6.98
363.55	8.62	363.65	11.45						

Table S 5. Calculated values of fluidity, η^{-1} and molar conductivity, Λ, as a function of temperature for the range of alkyl and ether functionalised $[TFSI]^-$-based ILs.

***T* / K**	***η*$^{-1}$ / Poise^{-1}**	***Λ* / S·cm^2·mol^{-1}**	***η*$^{-1}$ / Poise^{-1}**	***Λ* / S·cm^2·mol^{-1}**	***η*$^{-1}$ / Poise^{-1}**	***Λ* / S·cm^2·mol^{-1}**	***η*$^{-1}$ / Poise^{-1}**	***Λ* / S·cm^2·mol^{-1}**	***η*$^{-1}$ / Poise^{-1}**	***Λ* / S·cm^2·mol^{-1}**
	$[Pyrr_{14}]^+$		$[Pyrr_{1(2o1)}]^+$		$[Pyrr_{1(2o2o1)}]^+$		$[Pyrr_{(2o1)2}]^+$		$[Pyrr_{(2o2o1)2}]^+$	
293.15	1.023	0.653	1.625	0.877	1.552	0.753	1.035	0.425	0.564	0.380
298.15	1.296	0.818	2.032	1.076	1.954	0.929	1.341	0.563	0.803	0.494
303.15	1.615	1.007	2.496	1.300	2.415	1.128	1.706	0.725	1.101	0.629
313.15	2.402	1.461	3.600	1.829	3.524	1.598	2.631	1.127	1.886	0.964
323.15	3.399	2.020	4.941	2.464	4.884	2.167	3.848	1.631	2.936	1.388
333.15	4.620	2.685	6.511	3.206	6.491	2.832	5.383	2.234	4.252	1.905
343.15	6.070	3.454	8.301	4.052	8.339	3.593	7.258	2.931	5.823	2.513
***T* / K**	***η*$^{-1}$ / Poise^{-1}**	***Λ* / S·cm^2·mol^{-1}**	***η*$^{-1}$ / Poise^{-1}**	***Λ* / S·cm^2·mol^{-1}**	***η*$^{-1}$ / Poise^{-1}**	***Λ* / S·cm^2·mol^{-1}**	***η*$^{-1}$ / Poise^{-1}**	***Λ* / S·cm^2·mol^{-1}**	***η*$^{-1}$ / Poise^{-1}**	***Λ* / S·cm^2·mol^{-1}**
	$[Pip_{14}]^+$		$[Pip_{1(2o1)}]^+$		$[Pip_{1(2o2o1)}]^+$		$[Pip_{(2o1)2}]^+$		$[Pip_{(2o2o1)2}]^+$	
293.15	0.429	0.244	0.725	0.405	0.917	0.433	0.650	0.431	0.524	0.309
298.15	0.586	0.329	0.957	0.526	1.196	0.560	0.926	0.557	0.728	0.411
303.15	0.780	0.432	1.235	0.669	1.526	0.708	1.270	0.704	0.978	0.534
313.15	1.295	0.704	1.941	1.026	2.350	1.072	2.185	1.067	1.630	0.847
323.15	2.003	1.069	2.861	1.481	3.406	1.531	3.422	1.523	2.498	1.254
333.15	2.925	1.537	4.006	2.038	4.701	2.086	4.991	2.075	3.588	1.761
343.15	4.075	2.113	5.379	2.698	6.235	2.735	6.884	2.720	4.897	2.369
***T* / K**	***η*$^{-1}$ / Poise^{-1}**	***Λ* / S·cm^2·mol^{-1}**	***η*$^{-1}$ / Poise^{-1}**	***Λ* / S·cm^2·mol^{-1}**	***η*$^{-1}$ / Poise^{-1}**	***Λ* / S·cm^2·mol^{-1}**	***η*$^{-1}$ / Poise^{-1}**	***Λ* / S·cm^2·mol^{-1}**	***η*$^{-1}$ / Poise^{-1}**	***Λ* / S·cm^2·mol^{-1}**
	$[Aze_{14}]^+$		$[Aze_{1(2o1)}]^+$		$[Aze_{1(2o2o1)}]^+$		$[Aze_{(2o1)2}]^+$		$[Aze_{(2o2o1)2}]^+$	
293.15	0.226	0.140	0.546	0.255	0.577	0.289	0.524	0.190	0.333	0.175
298.15	0.326	0.198	0.741	0.350	0.783	0.388	0.737	0.259	0.476	0.241
303.15	0.455	0.271	0.980	0.466	1.035	0.507	1.002	0.344	0.659	0.324
313.15	0.822	0.474	1.604	0.767	1.695	0.810	1.712	0.566	1.162	0.547
323.15	1.360	0.762	2.441	1.166	2.586	1.207	2.684	0.866	1.876	0.855
333.15	2.101	1.147	3.508	1.665	3.725	1.702	3.938	1.251	2.826	1.258
343.15	3.070	1.638	4.811	2.266	5.124	2.296	5.481	1.726	4.027	1.763

Table S 6. Coefficients of the 3rd order polynomial correlation of the Arrhenius-type plot of the temperature dependence of viscosity as per the equation; $\ln(\eta) = A_o + A_1*1/T + A_2*(1/T)^2 + A_3*(1/T)^3$. The derived average activation energy barrier, E_a^η, is also shown.

Ionic liquid cation	A_o	A_1	A_2	A_3	E_a^η
$[Pyrr_{14}]^+$	-4.351	4.335	-1.476	0.285	30.21
$[Pyrr_{1(2o1)}]^+$	-15.485	15.468	-5.193	0.687	27.85
$[Pyrr_{1(2o2o1)}]^+$	-22.100	21.835	-7.256	0.912	28.73
$[Pyrr_{(2o1)2}]^+$	10.680	-9.844	2.827	-0.136	33.19
$[Pyrr_{(2o2o1)2}]^+$	-93.479	94.193	-31.800	3.713	41.15
$[Pip_{14}]^+$	-8.707	9.298	-3.428	0.562	38.42
$[Pip_{1(2o1)}]^+$	-15.926	16.149	-5.554	0.766	34.37
$[Pip_{1(2o2o1)}]^+$	36.812	-34.323	10.519	-0.943	32.31
$[Pip_{(2o1)2}]^+$	-48.661	49.776	-17.239	2.129	40.93
$[Pip_{(2o2o1)2}]^+$	75.809	-71.126	21.978	-2.109	37.46
$[Aze_{14}]^+$	-31.245	31.420	-10.741	1.389	44.82
$[Aze_{1(2o1)}]^+$	-8.245	9.310	-3.587	0.590	37.18
$[Aze_{1(2o2o1)}]^+$	-31.932	30.939	-10.178	1.259	37.64
$[Aze_{(2o1)2}]^+$	-22.731	24.254	-8.811	1.204	40.34
$[Aze_{(2o2o1)2}]^+$	-40.829	40.638	-13.720	1.702	43.04

Table S 7. Coefficients of the 3rd order polynomial correlation of the Arrhenius-type plot of the temperature dependence of molar conductivity as per the equation; $\ln(\Lambda) = A_o' + A_1'*1/T + A_2'*(1/T)^2 + A_3'*(1/T)^3$. The derived average activation energy barrier, E_a^Λ, is also shown.

Ionic liquid cation	A_o'	A_1'	A_2'	A_3'	E_a^Λ
$[Pyrr_{14}]^+$	6.446	-2.835	1.056	-0.239	28.27
$[Pyrr_{1(2o1)}]^+$	8.744	-4.655	1.499	-0.263	26.02
$[Pyrr_{1(2o2o1)}]^+$	12.309	-8.530	2.858	-0.422	26.60
$[Pyrr_{(2o1)2}]^+$	60.833	-56.899	18.992	-2.232	33.53
$[Pyrr_{(2o2o1)2}]^+$	14.193	-11.407	4.170	-0.624	32.32
$[Pip_{14}]^+$	-12.892	14.642	-4.085	0.228	36.55
$[Pip_{1(2o1)}]^+$	20.392	-16.902	5.808	-0.787	32.48
$[Pip_{1(2o2o1)}]^+$	59.319	-54.227	17.691	-2.041	31.92
$[Pip_{(2o1)2}]^+$	55.118	-50.292	16.464	-1.914	31.91
$[Pip_{(2o2o1)2}]^+$	-19.194	20.606	-5.985	0.438	34.50
$[Aze_{14}]^+$	-35.203	35.644	-10.578	0.875	41.66
$[Aze_{1(2o1)}]^+$	-98.501	96.223	-29.919	2.949	36.01
$[Aze_{1(2o2o1)}]^+$	15.933	-13.081	4.771	-0.707	35.44
$[Aze_{(2o1)2}]^+$	-2.050	4.764	-1.131	-0.068	37.43
$[Aze_{(2o2o1)2}]^+$	-32.601	33.747	-10.222	0.874	38.90

Table S 8. Correlation parameters, η_o^f, D^η, $T_o^{\eta f}$ and σ_o^f, D^σ, $T_o^{\sigma f}$, and the respective coefficients of determination R^2, for the temperature dependence of viscosity and conductivity, respectively, according to the modified VTF equation (Equations 9 and 10 in the main text).

**Ionic liquid cation**	η_o^f	D^η	$T_o^{\eta f}$	**Adj. R^2 (η)**	σ_o^f	D^σ	$T_o^{\sigma f}$	**Adj. R^2 (σ)**
$[Pyrr_{14}]^+$	0.126	5.37	162.21	0.99999	739.13	4.58	164.06	1.00000
$[Pyrr_{1(2o1)}]^+$	0.203	4.31	167.09	1.00000	581.18	3.94	167.56	1.00000
$[Pyrr_{1(2o2o1)}]^+$	0.163	4.71	163.77	1.00000	640.85	4.84	157.63	1.00000
$[Pyrr_{(2o1)2}]^+$	0.144	4.26	177.28	1.00000	611.85	4.00	177.18	0.99999
$[Pyrr_{(2o2o1)2}]^+$	0.093	5.01	175.20	0.99997	412.61	3.89	178.37	1.00000
$[Pip_{14}]^+$	0.108	5.34	172.88	1.00000	1007.98	4.96	173.14	1.00000
$[Pip_{1(2o1)}]^+$	0.197	4.15	179.41	1.00000	912.06	4.70	175.98	1.00000
$[Pip_{1(2o2o1)}]^+$	0.149	4.71	170.90	1.00000	604.97	4.41	170.87	1.00000
$[Pip_{(2o1)2}]^+$	0.096	4.33	184.38	0.99999	346.24	3.12	188.42	1.00000
$[Pip_{(2o2o1)2}]^+$	0.127	4.71	178.09	0.99999	526.87	4.20	178.85	1.00000
$[Aze_{14}]^+$	0.085	5.48	178.54	1.00000	1192.79	5.05	178.95	1.00000
$[Aze_{1(2o1)}]^+$	0.123	4.97	174.35	1.00000	1163.16	5.05	172.82	0.99999
$[Aze_{1(2o2o1)}]^+$	0.098	5.39	170.11	1.00000	875.65	5.03	170.13	1.00000
$[Aze_{(2o1)2}]^+$	0.127	4.22	185.60	1.00000	480.32	3.98	185.18	1.00000
$[Aze_{(2o2o1)2}]^+$	0.066	5.74	174.18	1.00000	823.43	5.14	174.82	1.00000

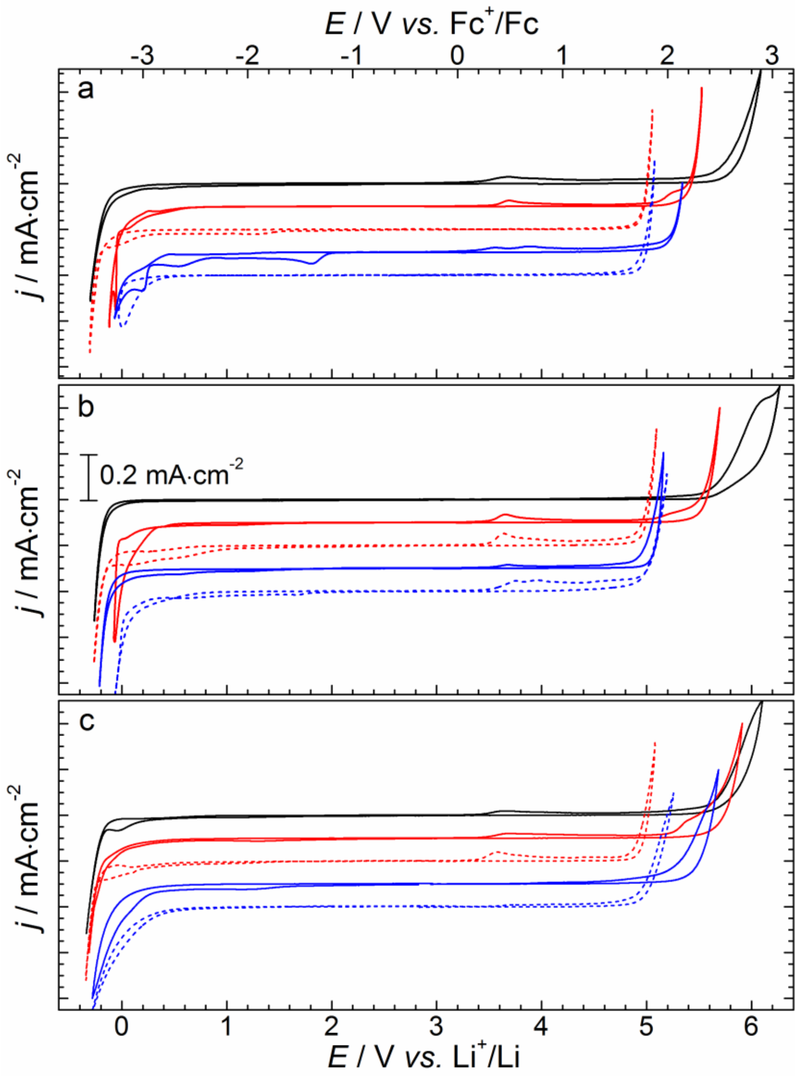

Figure S 3. Electrochemical windows of the pyrrolidinium (a) pyrrolidinium, (b) piperidinium and (c) azepanium-based [TFSI]$^-$-based ILs at the 3 mm diameter glassy carbon macro-disk working electrode. Line styles represent the represent the functional groups of a given cyclic alkylammonium cation; — = 14, — = 1(2o1), - - -= 1(2o2o1), — = $(2o1)_2$, - - - = $(2o2o1)_2$. A scan rate of 2 mV·s^{-1} was utilised in all cases.

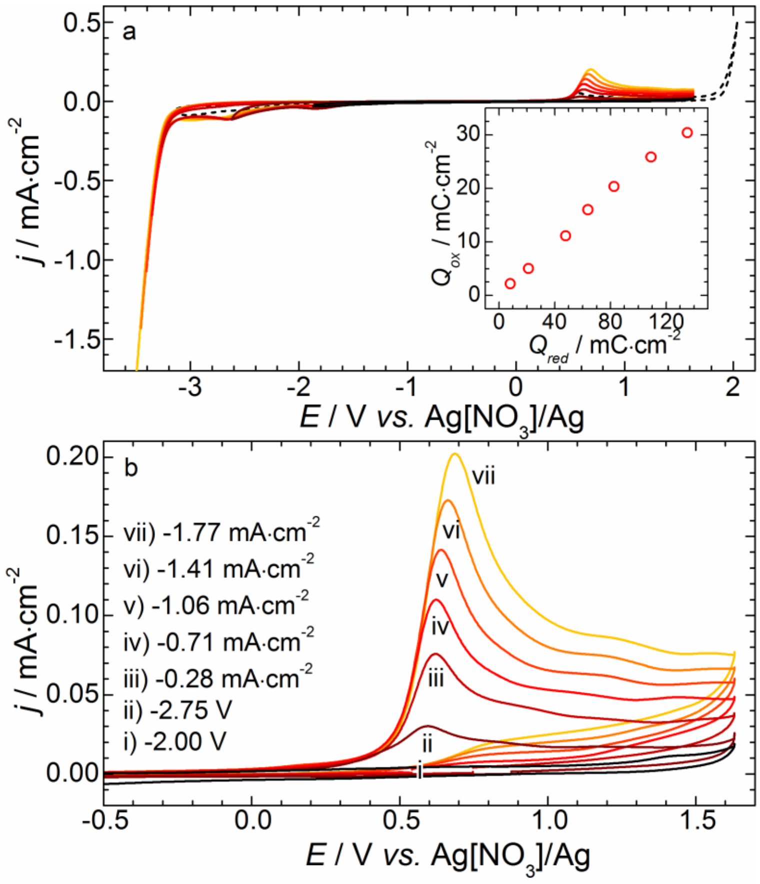

Figure S 4. a) Cyclic voltammetry of [$Pip_{1(2o2o1)}$][TFSI] at a glassy carbon working electrode wherein the reductive limits are increased. The dashed line shows the full electrochemical stability window of the IL. b) Expanded view of the voltage range between -0.5 and 1.7 V vs. Ag[NO_3]/Ag showing the oxidation of the supposed impurity. The legend in b) highlights the reductive current (or voltage) limits of the negative scan completed before the displayed oxidative sweep. The inset graph in a) shows the relationship between the integral charge passed upon the reductive sweep (Q_{red}) and the charge passed upon the oxidation of the supposed impurity (Q_{ox}).

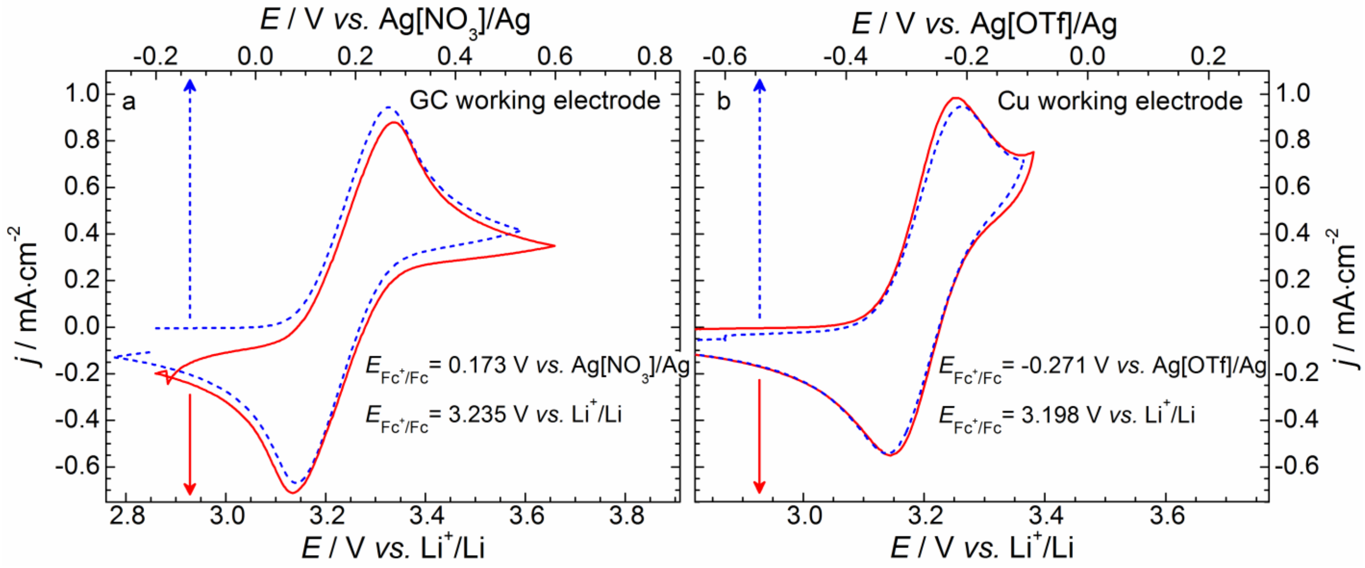

Figure S 5. Cyclic voltammetry of the ferrocene redox couple in $[Pyrr_{1(2o2o1)}]$[TFSI] as measured at (a) glassy carbon and (b) copper working electrodes versus a Li-metal strip reference electrode (solid red line, bottom *x*-axes) and either (a) the Ag[NO_3]/Ag or (b) the Ag[OTf]/Ag reference electrodes (dashed blue lines, top *y*-axes). A scan rate of 5 mV·s^{-1} was utilised and, for (a), a small amount of Li[TFSI] was dissolved in the IL prior to measurement using the Li-reference electrode. An estimation of the formal redox potential ($E_{Fc+/Fc}$) versus each reference electrode was calculated by taking the average of the oxidation and reduction peak potentials (i.e. $E_{Fc+/Fc} = (E_{pa} + E_{pc})/2$ where E_{pa} and E_{pc} represent the peak potentials of oxidation and reduction processes, respectively).

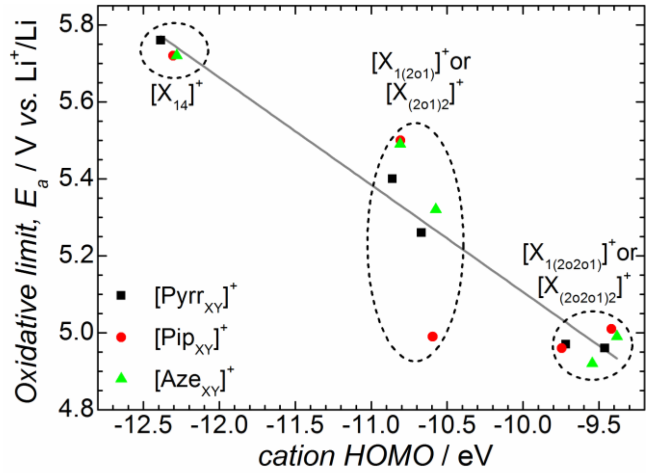

Figure S 6. Computed single ion (cation) HOMO energy levels vs. the oxidative potential limit, E_a, of the analogous [TFSI]$^-$ IL at the glassy carbon macro-disk electrode. The dashed circles highlight the grouping of IL data based on the length and number of ether functional groups where the symbol colour represents the different alkylammonium ring size.

Table S 9. HOMO and LUMO energies and surfaces of selected ions.

$[Pyrr_{14}]^+$				
	HOMO		LUMO	
Conformer	Surface	Energy (eV)	Surface	Energy (eV)
1	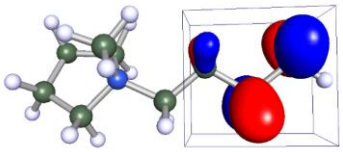	-12.294	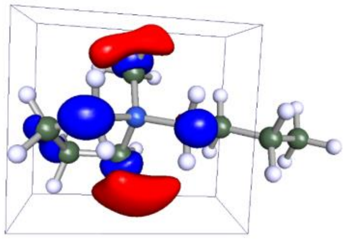	-3.535
2	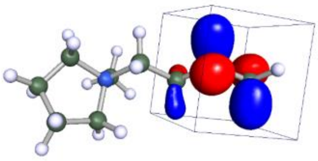	-12.300	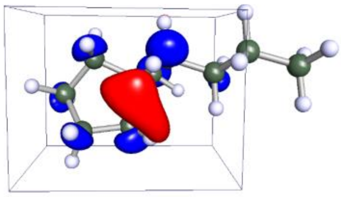	-3.396
3	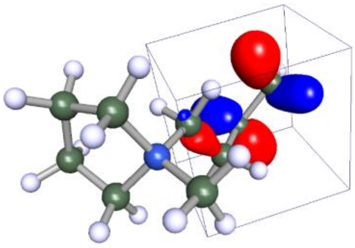	-12.403	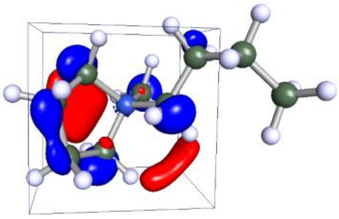	-3.467
4	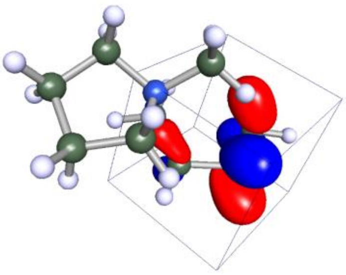	-12.547	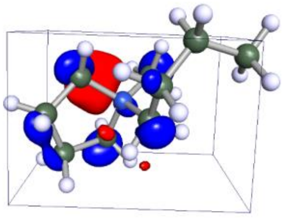	-3.459
Average		-12.386		-3.464

Table S 9. *Continued...*

	[$Pyrr_{1(2o1)}$]$^{+}$			
	HOMO		LUMO	
Conformer	Surface	Energy (eV)	Surface	Energy (eV)
1	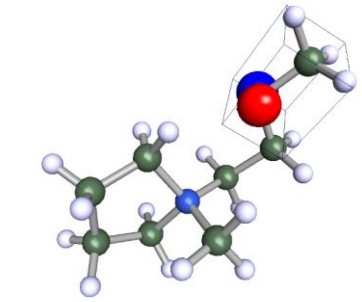	-10.942	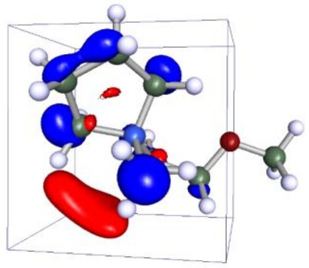	-3.478
2	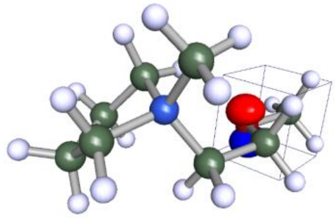	-10.939	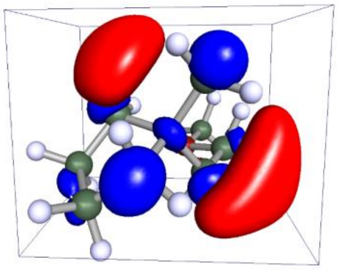	-3.429
3	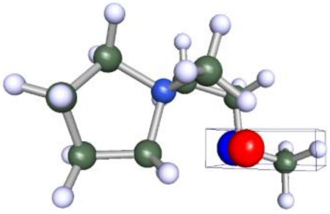	-10.966	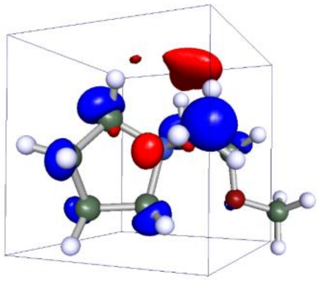	-3.287
4	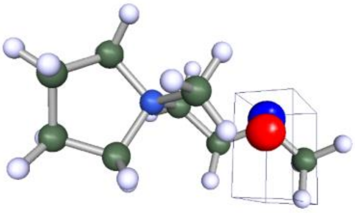	-10.836	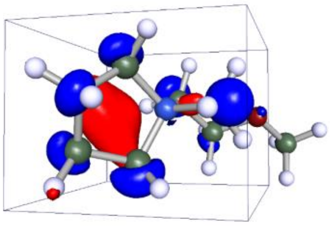	-3.355

Table S 9. *Continued...*

	$[Pyrr_{1(2o1)}]^+$			
	HOMO		LUMO	
Conformer	Surface	Energy (eV)	Surface	Energy (eV)
5	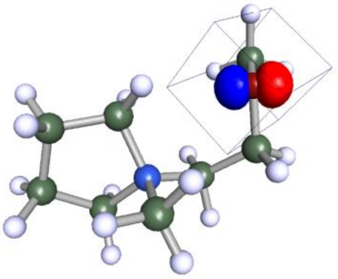	-11.002	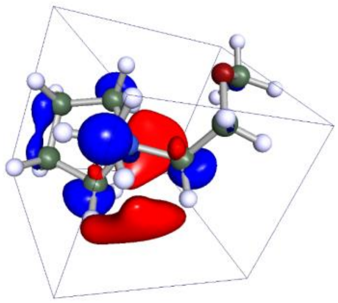	-3.573
6	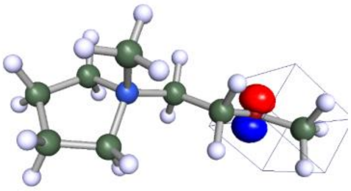	-10.686	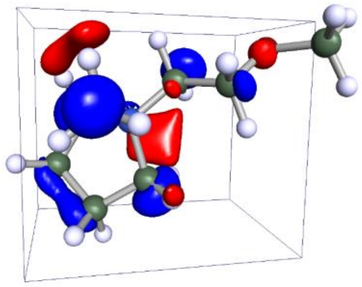	-3.641
7	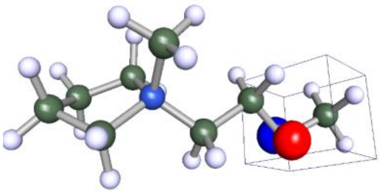	-10.700	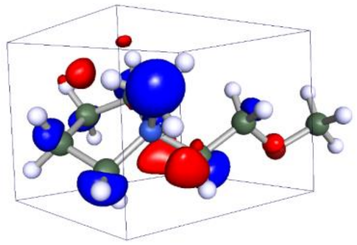	-3.472
8	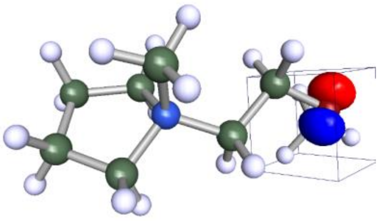	-10.808	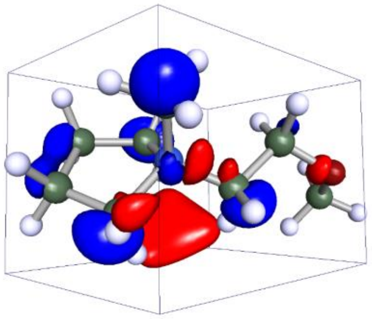	-3.706
Average		-10.860		-3.493

Table S 9. *Continued...*

$[Pyrr_{1(2o2o1)}]^+$				
	HOMO		LUMO	
Conformer	Surface	Energy (eV)	Surface	Energy (eV)
1	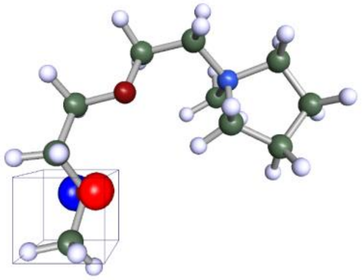	-9.739	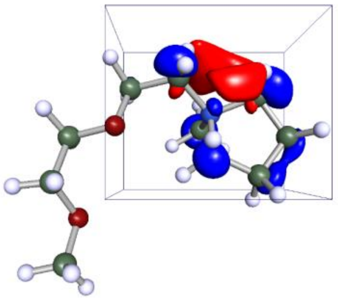	-3.336
2	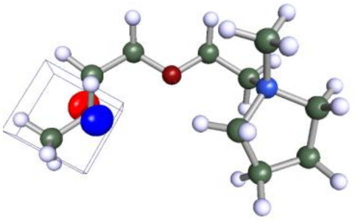	-9.608	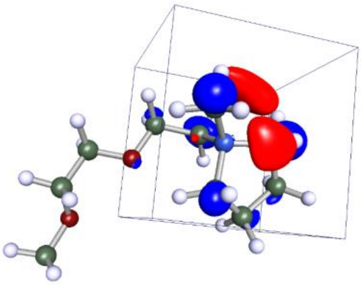	-3.344
3	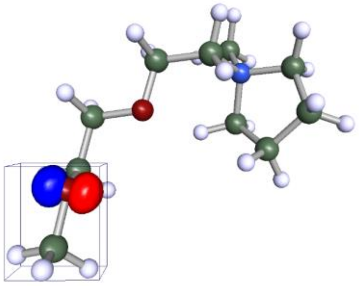	-9.551	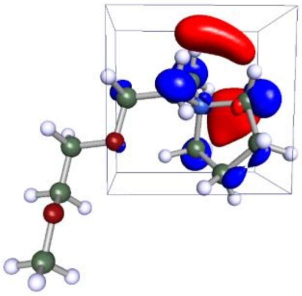	-3.355
4	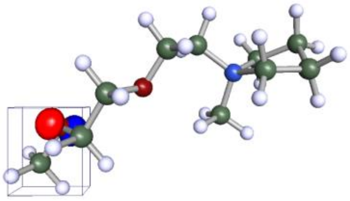	-9.497	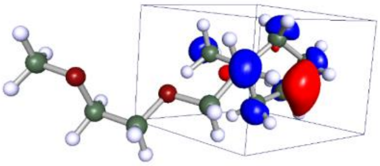	-3.252

Table S 9. *Continued...*

$[Pyrr_{1(2o2o1)}]^+$				
	HOMO		LUMO	
Conformer	Surface	Energy (eV)	Surface	Energy (eV)
5	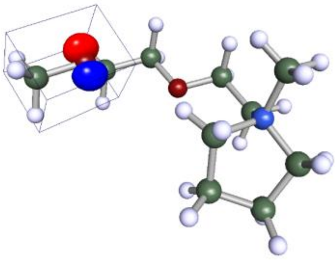	-9.704	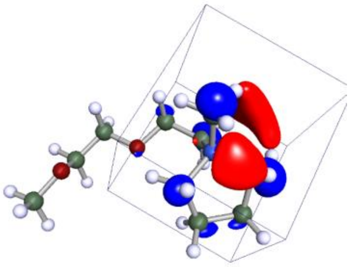	-3.303
6	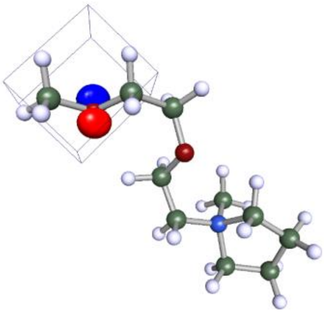	-9.780	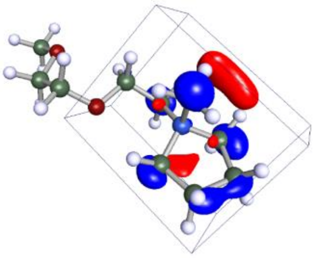	-3.325
7	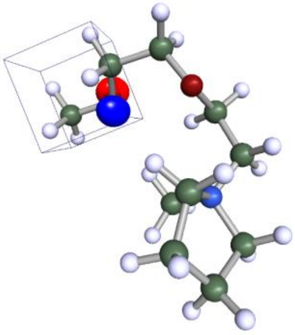	-10.283	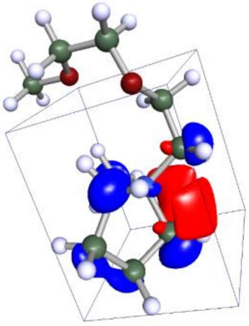	-3.282
8	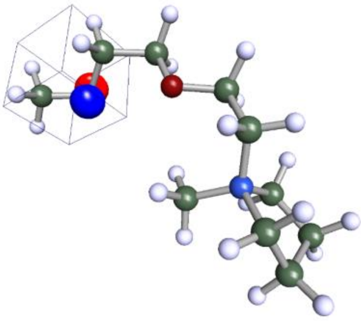	-9.595	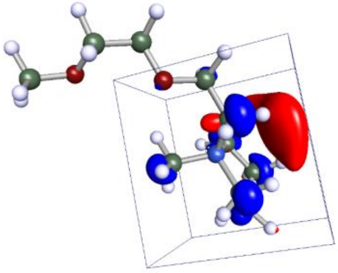	-3.249

Table S 9. *Continued...*

$[Pyrr_{1(2o2o1)}]^+$				
	HOMO		LUMO	
Conformer	Surface	Energy (eV)	Surface	Energy (eV)
9	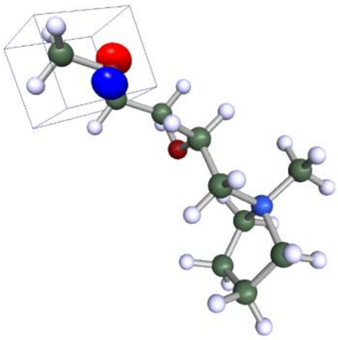	-9.796	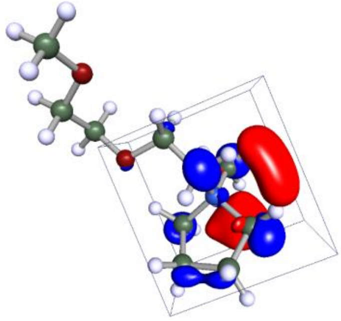	-3.274
10	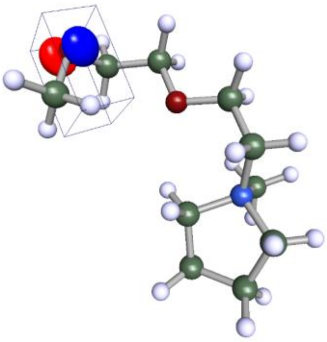	-9.622	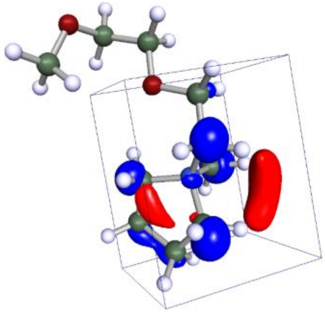	-3.529
Average		-9.717		-3.325

$[Pyrr_{(2o1)2}]^+$				
	HOMO		LUMO	
Conformer	Surface	Energy (eV)	Surface	Energy (eV)
1	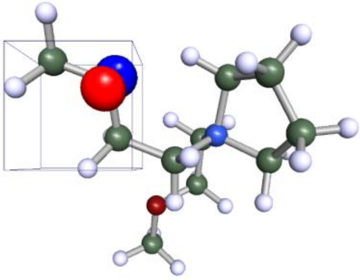	-10.615	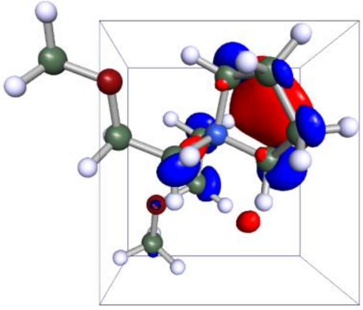	-3.146

Table S 9. *Continued...*

[Pyrr$_{(2o1)2}$]$^{+}$				
	HOMO		LUMO	
Conformer	Surface	Energy (eV)	Surface	Energy (eV)
2	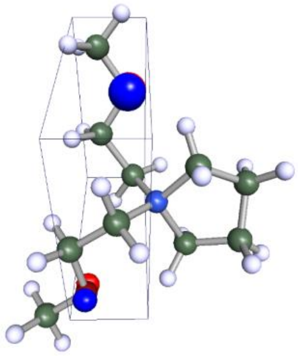	-10.795	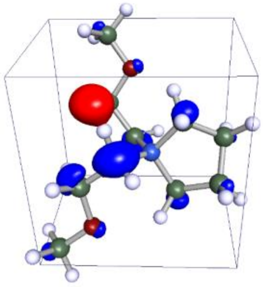	-2.936
3	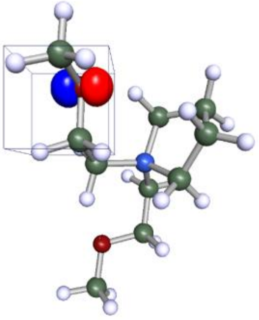	-10.757	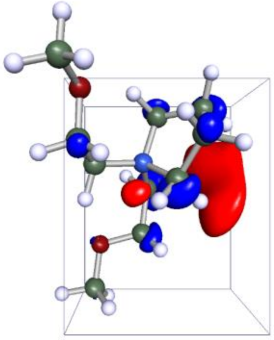	-2.963
4	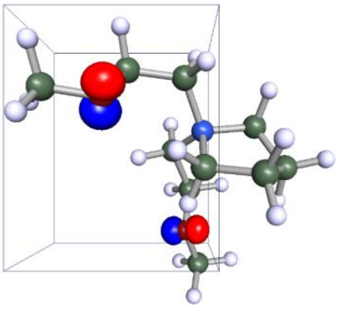	-10.808	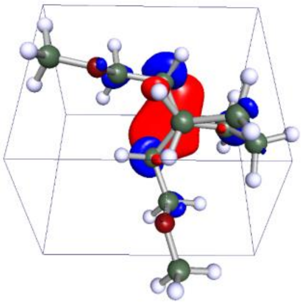	-2.980
5	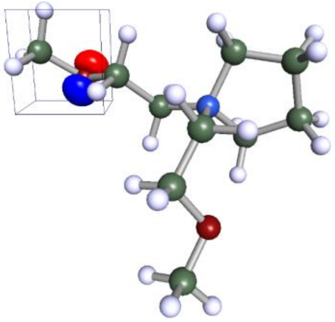	-10.618	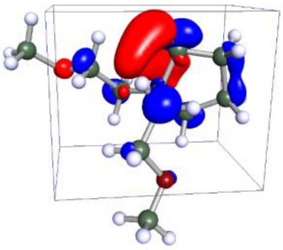	-3.189

Table S 9. *Continued...*

		$[Pyrr_{(2o1)2}]^+$		
	HOMO		LUMO	
Conformer	Surface	Energy (eV)	Surface	Energy (eV)
6	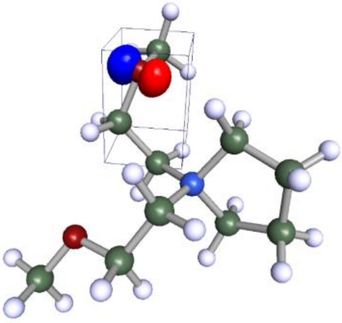	-10.656	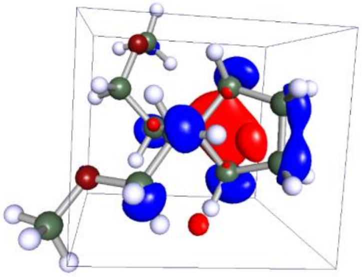	-3.241
7	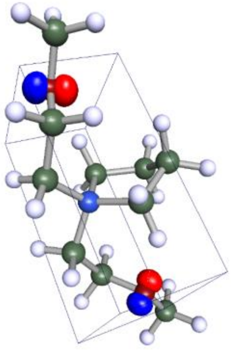	-10.800	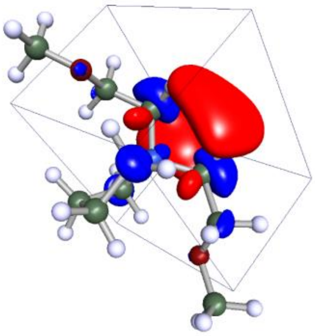	-2.947
8	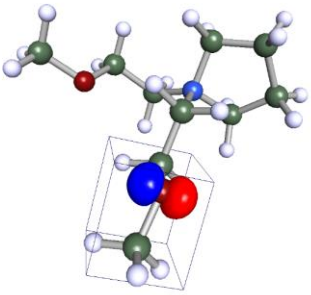	-10.370	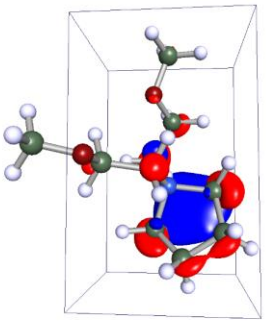	-3.293
9	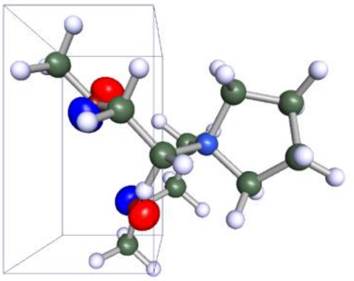	-10.604	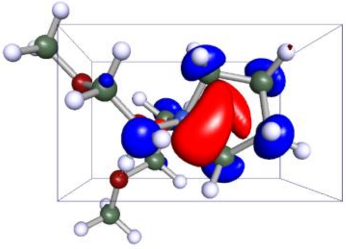	-3.031
Average		-10.669		-3.081

Table S 9. *Continued...*

$[Pyrr_{(2o2o1)2}]^+$				
	HOMO		LUMO	
Conformer	Surface	Energy (eV)	Surface	Energy (eV)
1	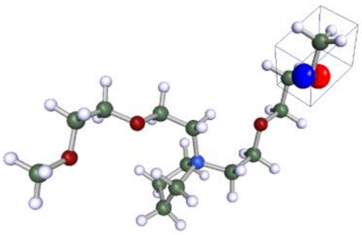	-9.423	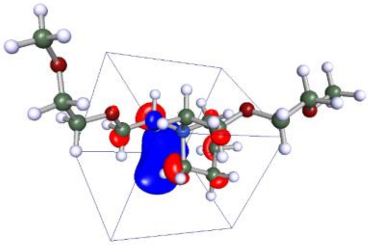	-2.778
2	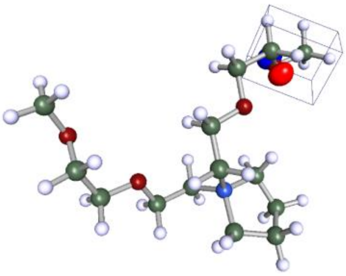	-9.459	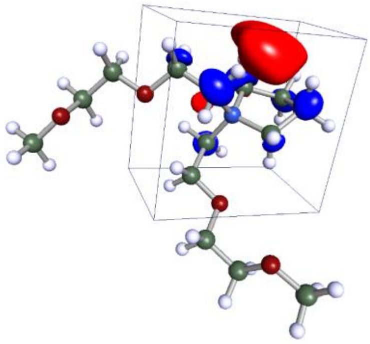	-2.852
3	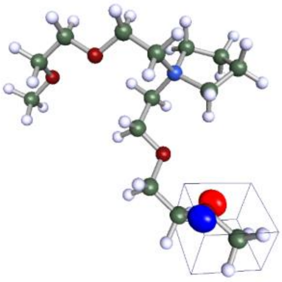	-9.431	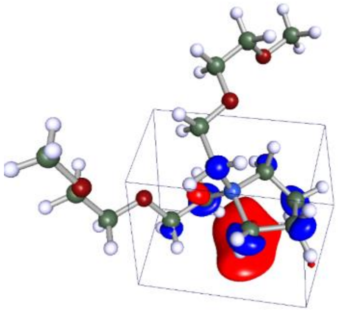	-2.803
4	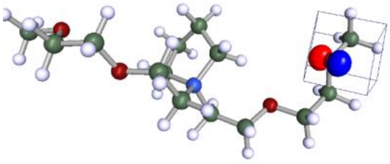	-9.535	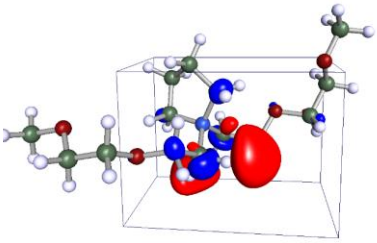	-2.691

Table S 9. *Continued...*

$[Pyrr_{(2o2o1)2}]^+$				
	HOMO		LUMO	
Conformer	Surface	Energy (eV)	Surface	Energy (eV)
5	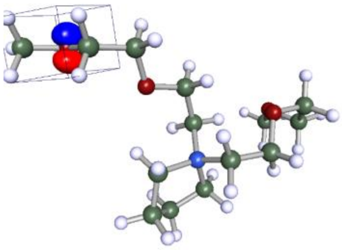	-9.298	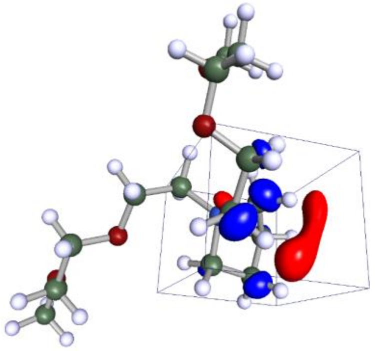	-2.871
6	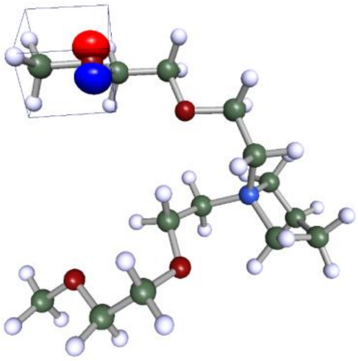	-9.442	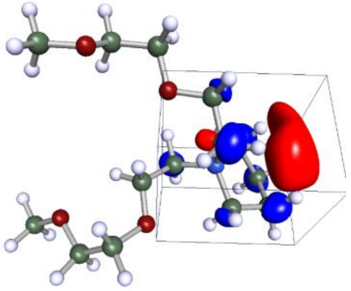	-2.844
7	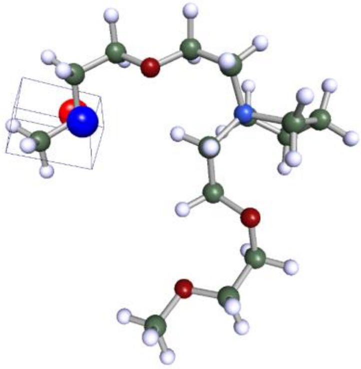	-9.568	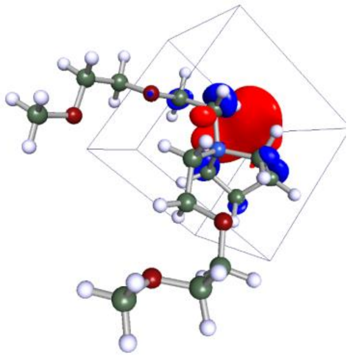	-2.743
8	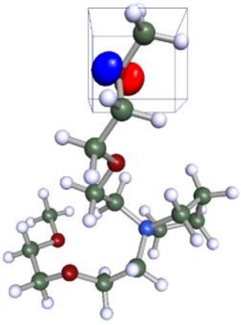	-9.323	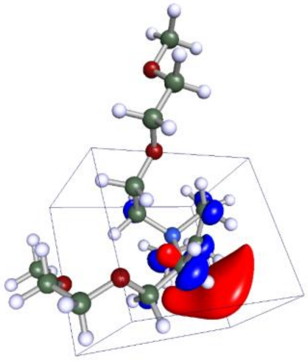	-2.827

Table S 9. *Continued...*

[Pyrr$_{(2o2o1)2}$]$^+$				
	HOMO		LUMO	
Conformer	Surface	Energy (eV)	Surface	Energy (eV)
9	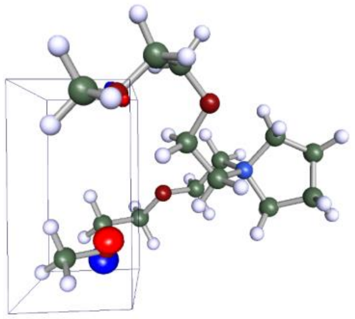	-9.573	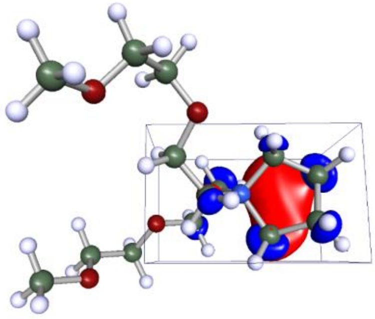	-2.800
10	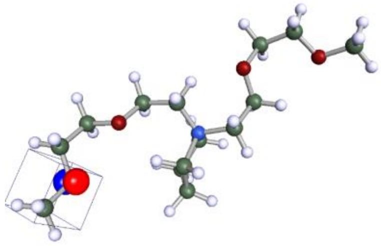	-9.554	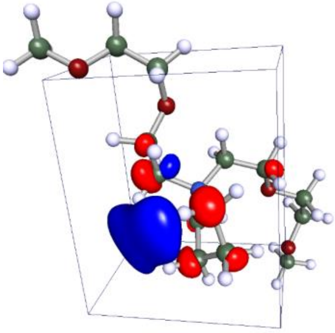	-2.659
Average		-9.461		-2.787

Table S 9. *Continued...*

[Pip$_{14}$]$^+$				
	HOMO		LUMO	
Conformer	Surface	Energy (eV)	Surface	Energy (eV)
1	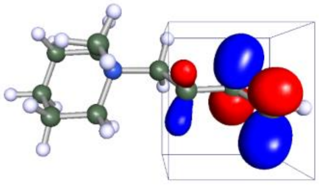	-12.264	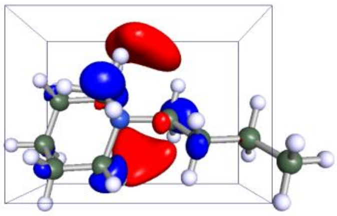	-3.274
2	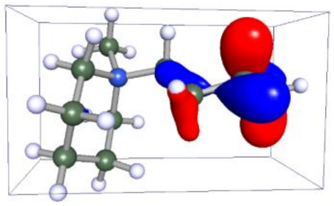	-12.346	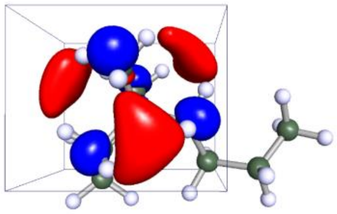	-3.399
Average		-12.305		-3.336

[Pip$_{1(2o1)}$]$^+$				
	HOMO		LUMO	
Conformer	Surface	Energy (eV)	Surface	Energy (eV)
1	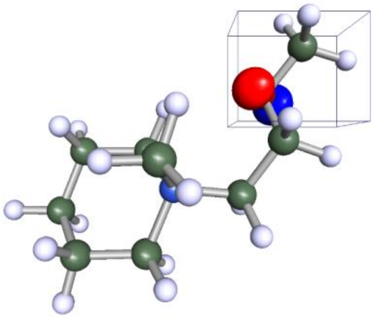	-10.898	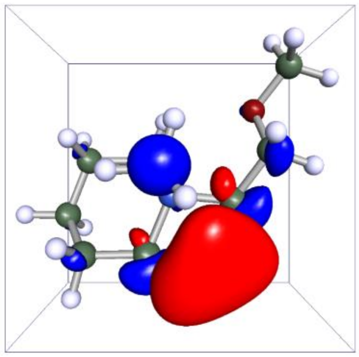	-3.263

Table S 9. *Continued...*

	[Pip$_{1(2o1)}$]$^+$			
	HOMO		LUMO	
Conformer	Surface	Energy (eV)	Surface	Energy (eV)
2	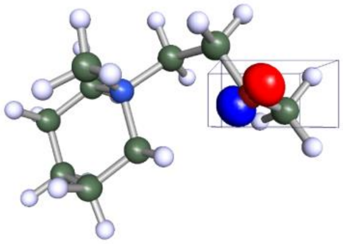	-10.923	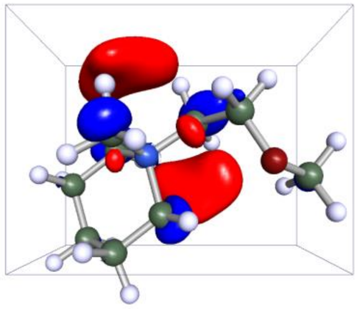	-3.361
3	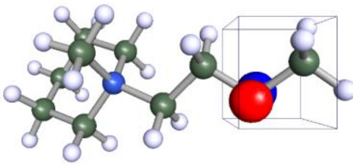	-10.645	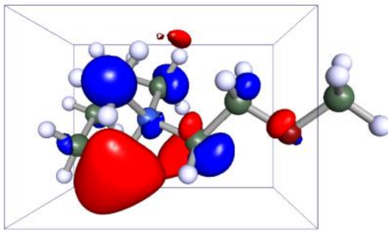	-3.380
4	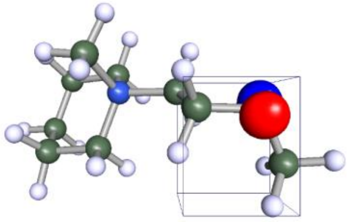	-10.762	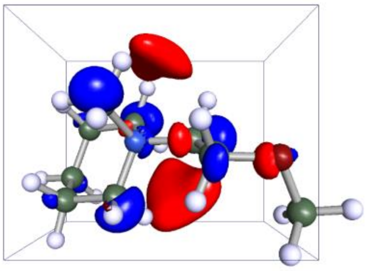	-3.440
Average		-10.807		-3.361

Table S 9. *Continued...*

$[Pip_{1(2o2o1)}]^+$				
	HOMO		LUMO	
Conformer	Surface	Energy (eV)	Surface	Energy (eV)
1	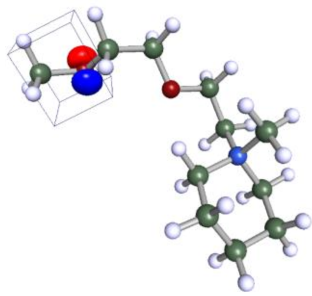	-9.540	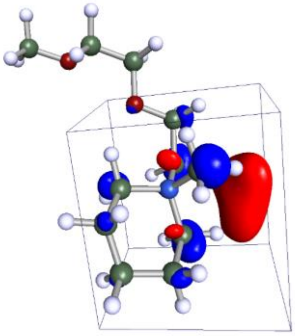	-3.184
2	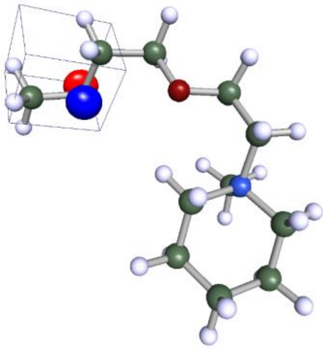	-9.682	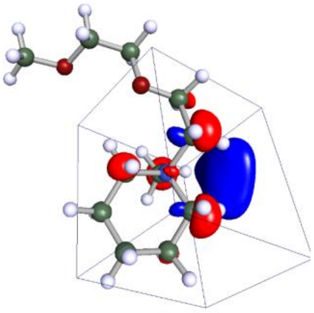	-3.132
3	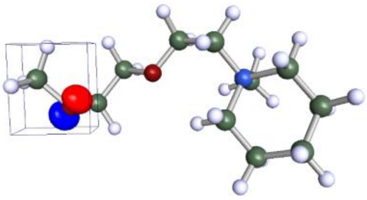	-9.587	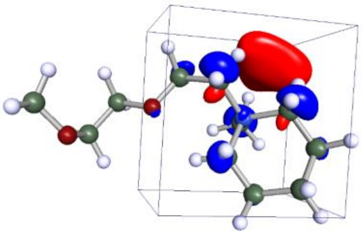	-3.206
4	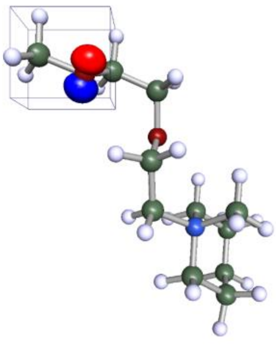	-9.761	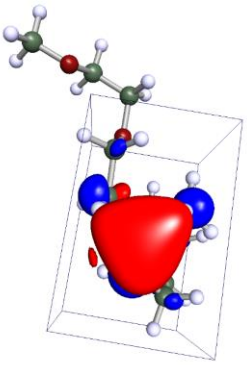	-3.094

Table S 9. *Continued...*

$[Pip_{1(2o2o1)}]^+$				
	HOMO		LUMO	
Conformer	Surface	Energy (eV)	Surface	Energy (eV)
5	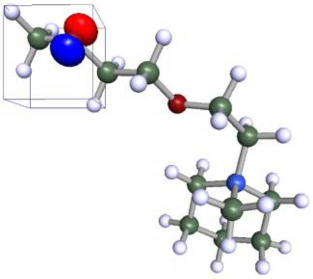	-9.519	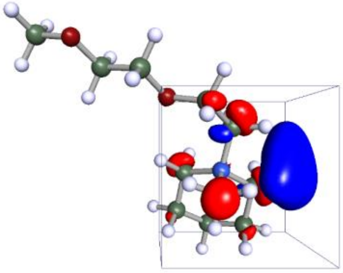	-3.260
6	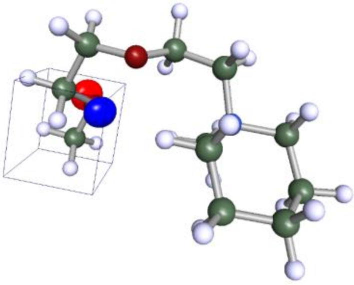	-10.226	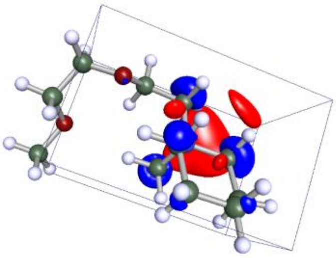	-3.053
7	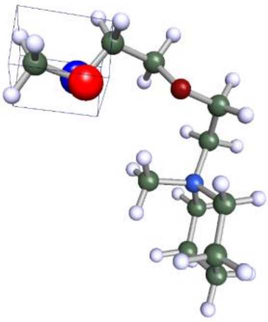	-9.772	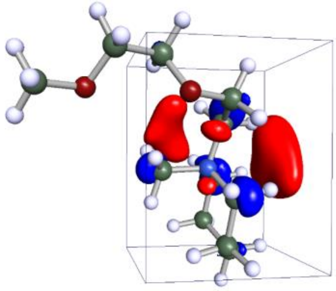	-3.173
8	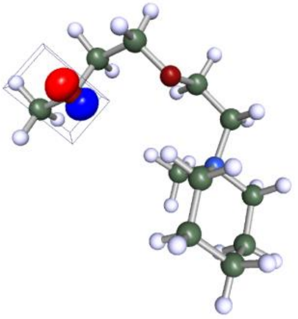	-9.997	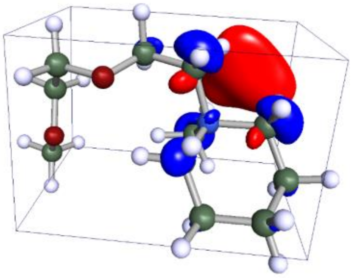	-3.113

Table S 9. *Continued...*

$[Pip_{1(2o2o1)}]^{+}$				
	HOMO		LUMO	
Conformer	Surface	Energy (eV)	Surface	Energy (eV)
9	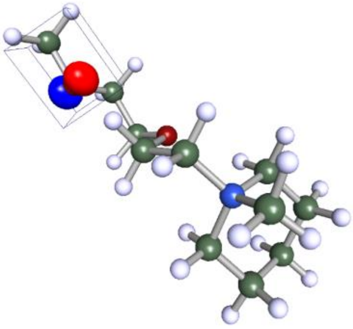	-9.780	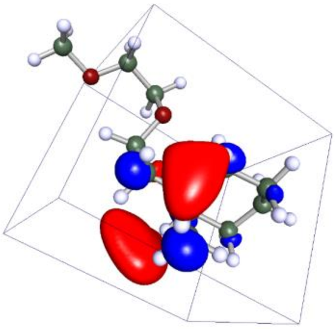	-3.088
10	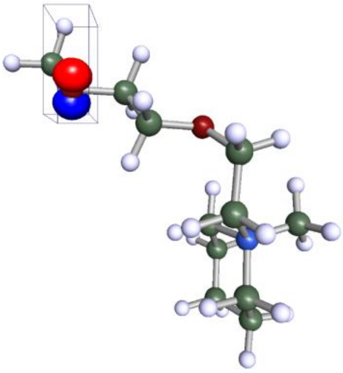	-9.584	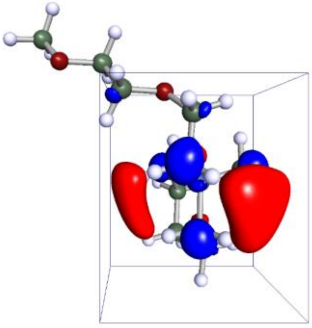	-3.306
Average		-9.745		-3.161

$[Pip_{(2o1)2}]^{+}$				
	HOMO		LUMO	
Conformer	Surface	Energy (eV)	Surface	Energy (eV)
1	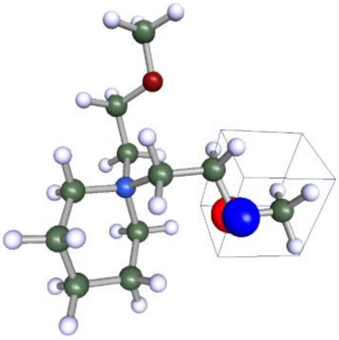	-10.531	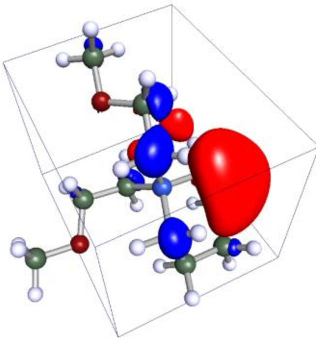	-3.018

Table S 9. *Continued...*

	$[Pip_{(2o1)2}]^+$			
	HOMO		LUMO	
Conformer	Surface	Energy (eV)	Surface	Energy (eV)
2	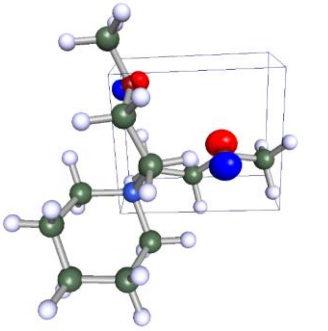	-10.642	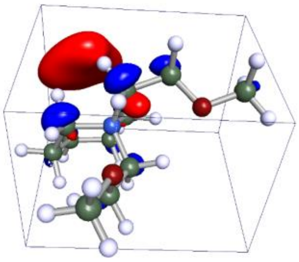	-2.912
3	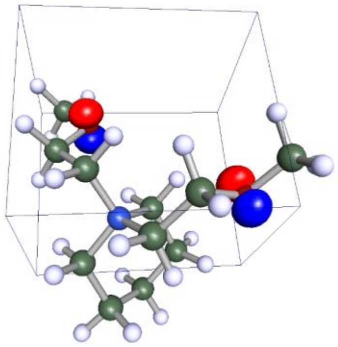	-10.664	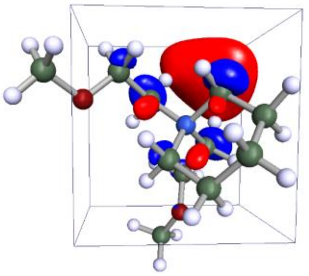	-3.010
4	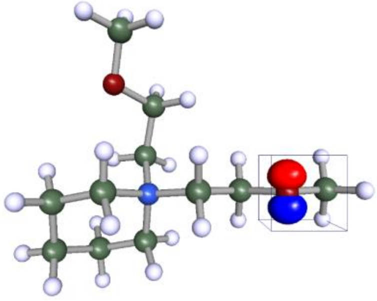	-10.585	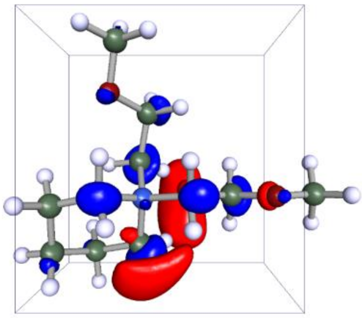	-3.001
5	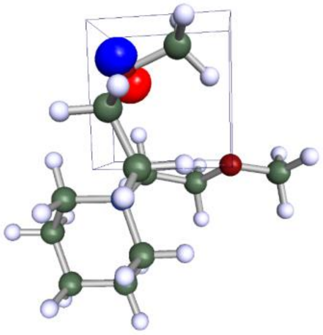	-10.574	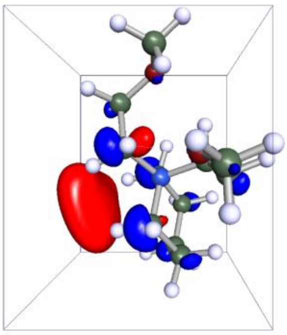	-2.933

Table S 9. *Continued...*

$[Pip_{(2o1)2}]^+$				
	HOMO		LUMO	
Conformer	Surface	Energy (eV)	Surface	Energy (eV)
6	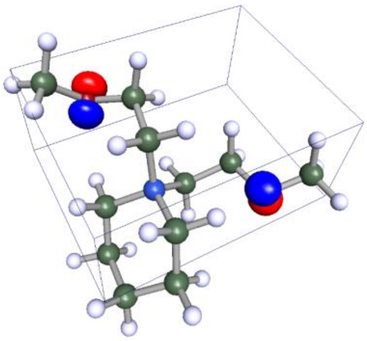	-10.803	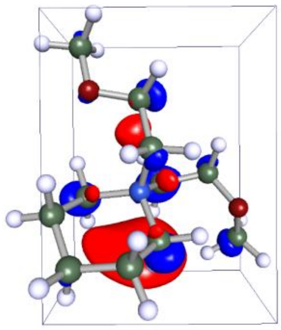	-3.007
7	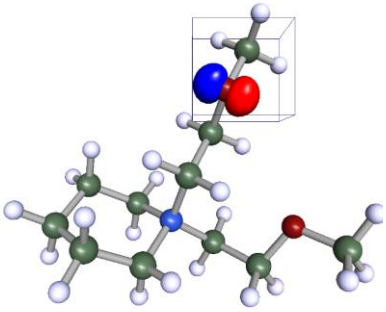	-10.324	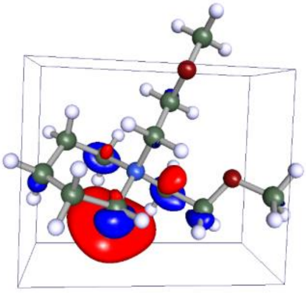	-3.094
8	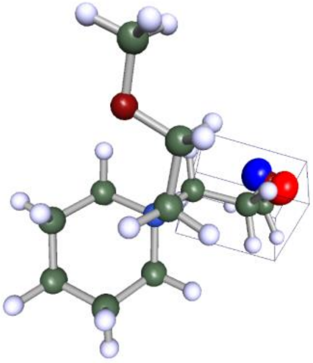	-10.667	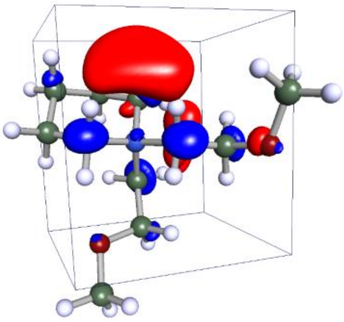	-3.072
9	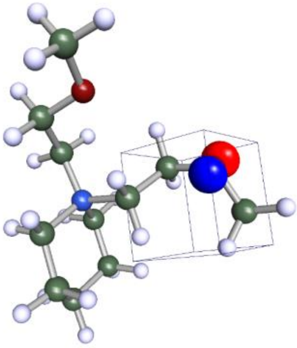	-10.457	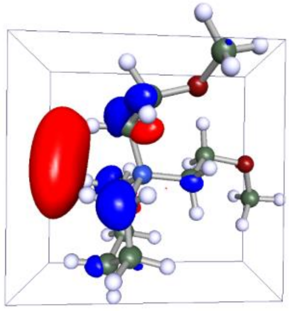	-3.124

Table S 9. *Continued...*

$[Pip_{(2o1)2}]^+$				
	HOMO		LUMO	
Conformer	Surface	Energy (eV)	Surface	Energy (eV)
10	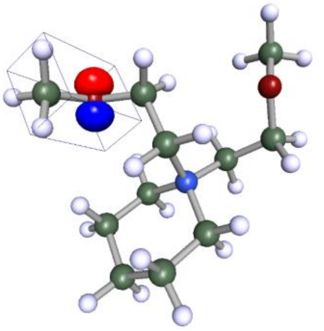	-10.705	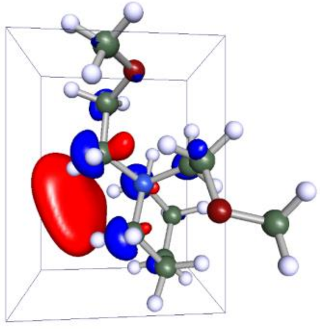	-3.026
Average		-10.595		-3.020

$[Pip_{(2o2o1)2}]^+$				
	HOMO		LUMO	
Conformer	Surface	Energy (eV)	Surface	Energy (eV)
1	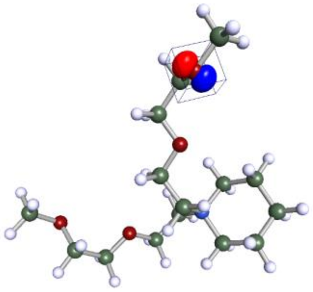	-9.353	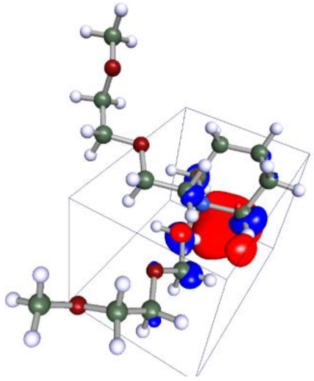	-2.895
2	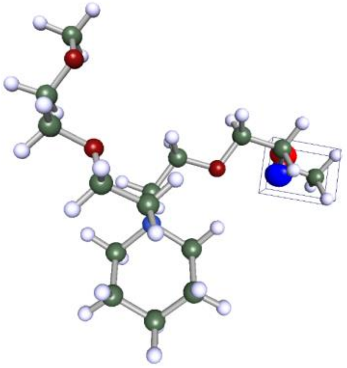	-9.344	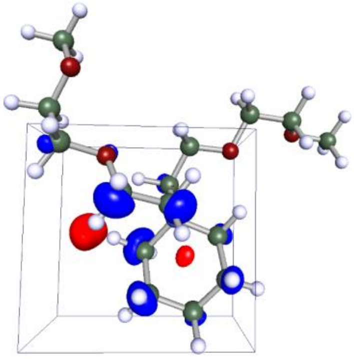	-2.765

Table S 9. *Continued...*

	$[Pip_{(2o2o1)2}]^+$			
	HOMO		LUMO	
Conformer	Surface	Energy (eV)	Surface	Energy (eV)
3	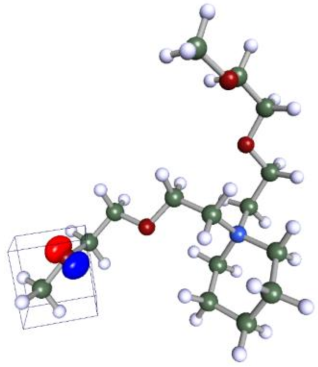	-9.339	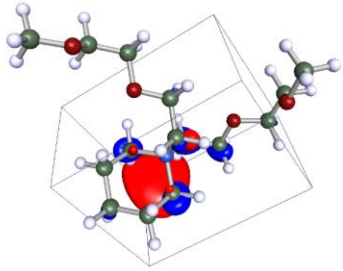	-2.844
4	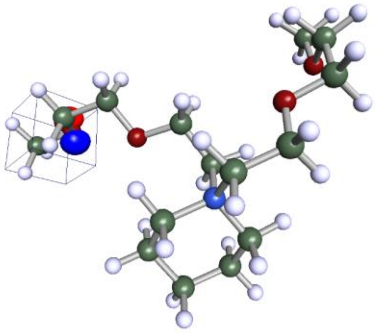	-9.344	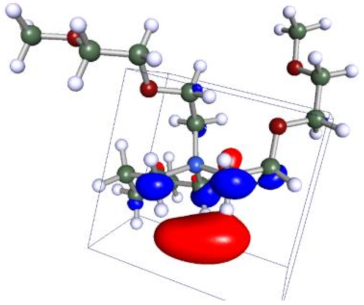	-2.846
5	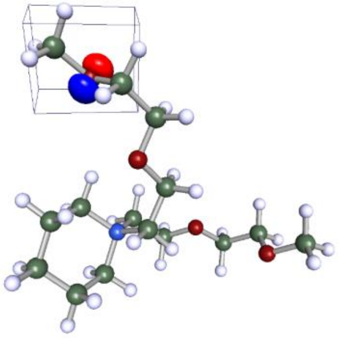	-9.472	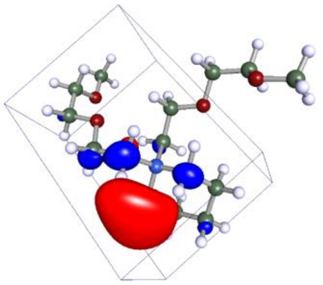	-2.778
6	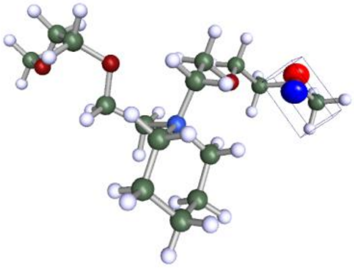	-9.369	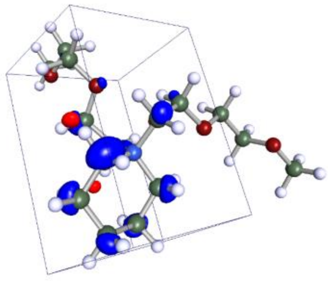	-2.642

Table S 9. *Continued...*

$[Pip_{(2o2o1)2}]^+$				
	HOMO		LUMO	
Conformer	Surface	Energy (eV)	Surface	Energy (eV)
7	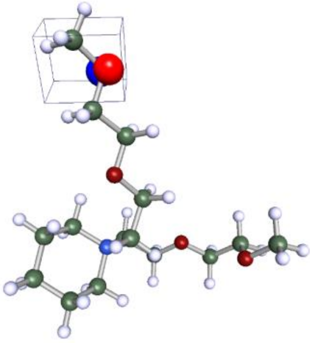	-9.347	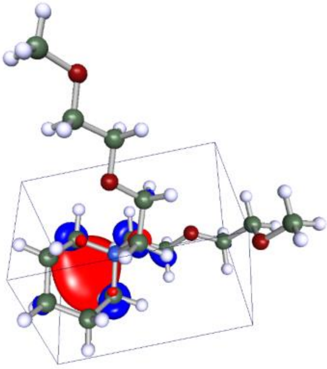	-2.882
8	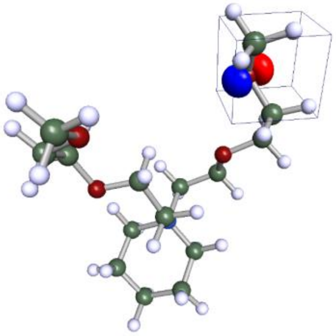	-9.442	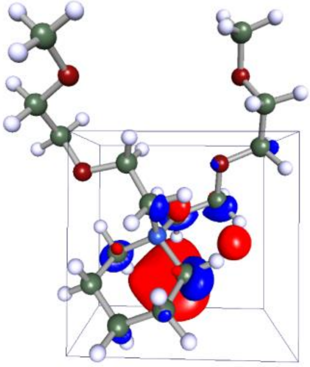	-2.816
9	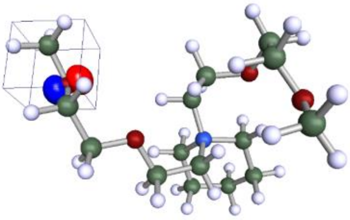	-9.704	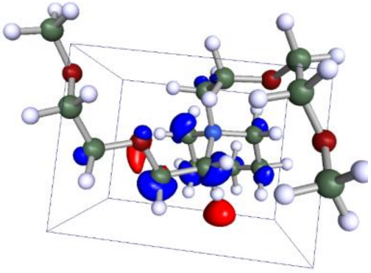	-2.669
10	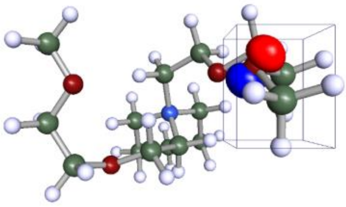	-9.475	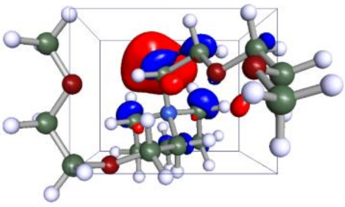	-2.765
Average		-9.419		-2.790

Table S 9. *Continued...*

$[Aze_{14}]^+$				
	HOMO		LUMO	
Conformer	Surface	Energy (eV)	Surface	Energy (eV)
1	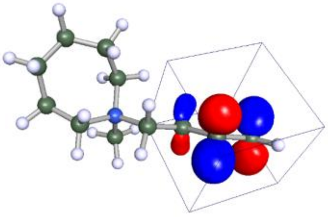	-12.248	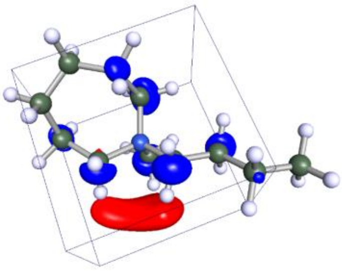	-3.124
2	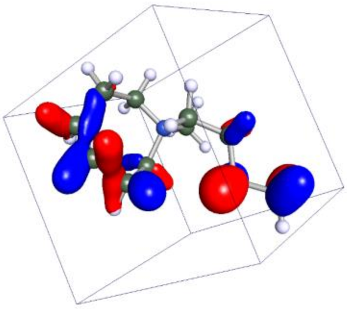	-12.313	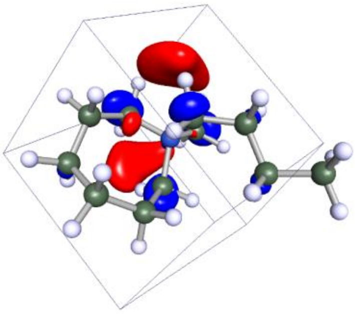	-3.241
Average		-12.281		-3.182

$[Aze_{1(2o1)}]^+$				
	HOMO		LUMO	
Conformer	Surface	Energy (eV)	Surface	Energy (eV)
1	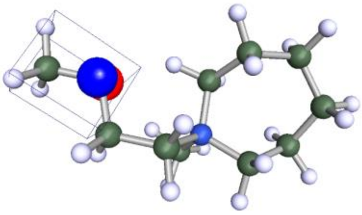	-10.895	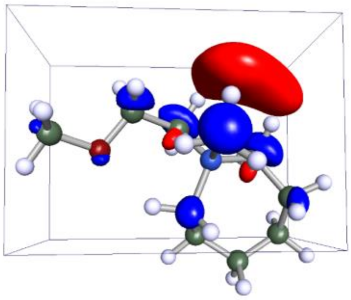	-3.083

Table S 9. *Continued...*

$[Aze_{1(2o1)}]^+$				
	HOMO		LUMO	
Conformer	Surface	Energy (eV)	Surface	Energy (eV)
2	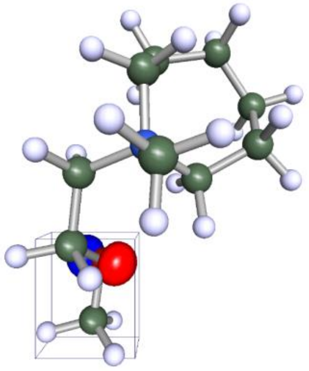	-10.776	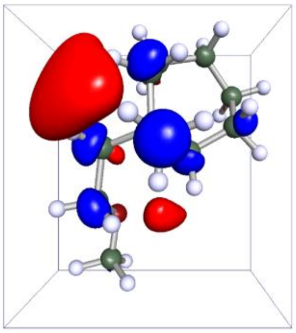	-3.132
3	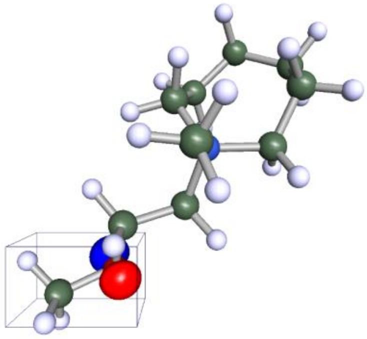	-10.637	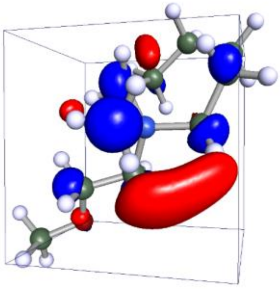	-3.206
4	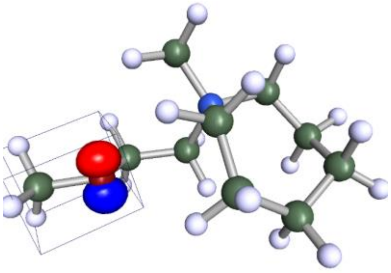	-10.923	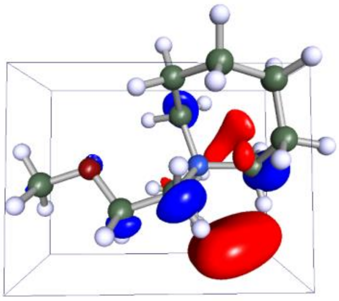	-3.208
5	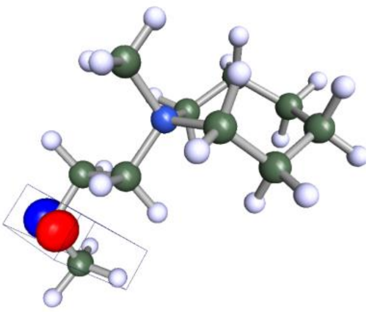	-10.724	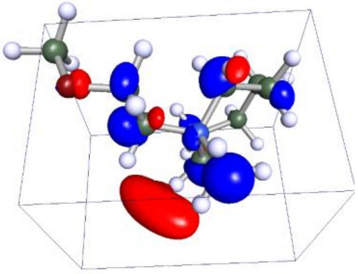	-3.222

Table S 9. *Continued...*

$[Aze_{1(2o1)}]^+$				
	HOMO		LUMO	
Conformer	Surface	Energy (eV)	Surface	Energy (eV)
6	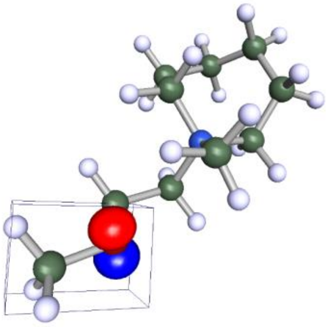	-10.778	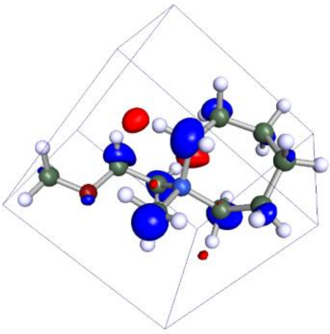	-3.176
7	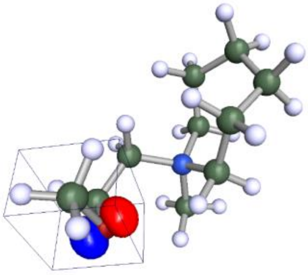	-10.934	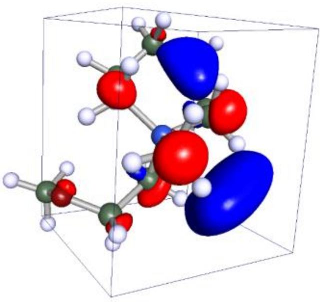	-3.230
Average		-10.810		-3.179

$[Aze_{1(2o2o1)}]^+$				
	HOMO		LUMO	
Conformer	Surface	Energy (eV)	Surface	Energy (eV)
1	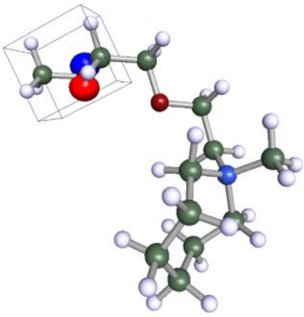	-9.464	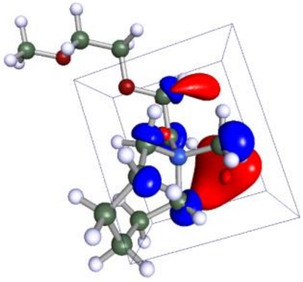	-3.072

Table S 9. *Continued...*

	$[Aze_{1(2o2o1)}]^+$			
	HOMO		LUMO	
Conformer	Surface	Energy (eV)	Surface	Energy (eV)
2	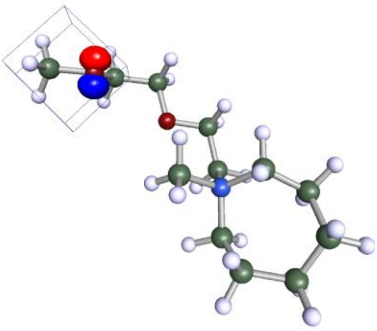	-9.543	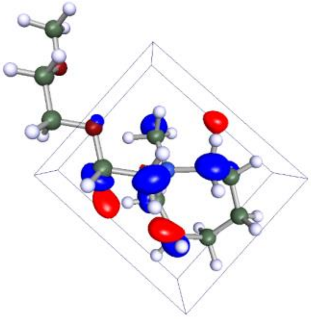	-2.933
3	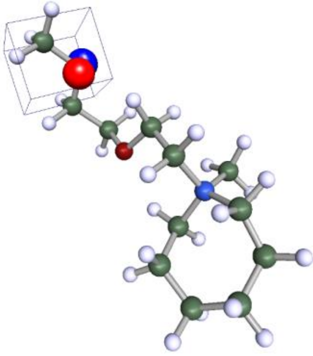	-9.753	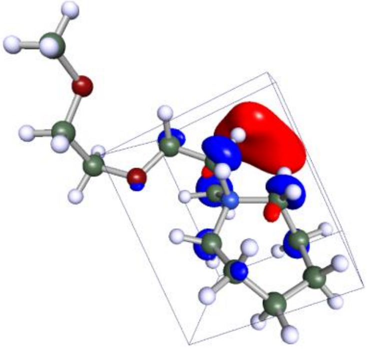	-2.906
4	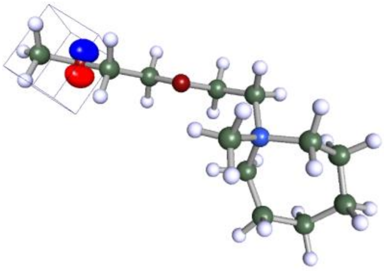	-9.497	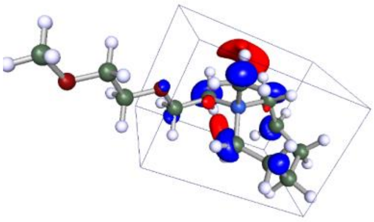	-3.012
5	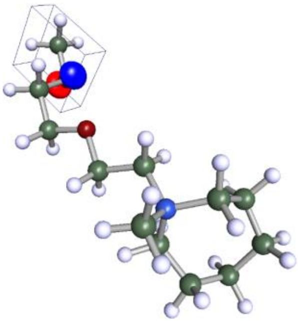	-9.268	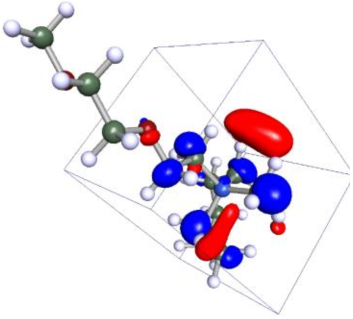	-3.108

Table S 9. *Continued...*

[Aze$_{1(2o2o1)}$]$^+$				
	HOMO		LUMO	
Conformer	Surface	Energy (eV)	Surface	Energy (eV)
6	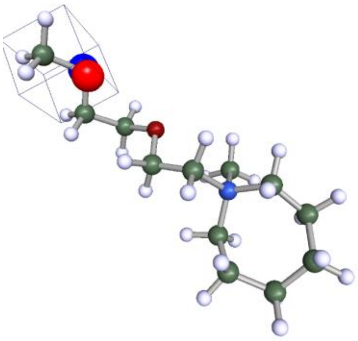	-9.540	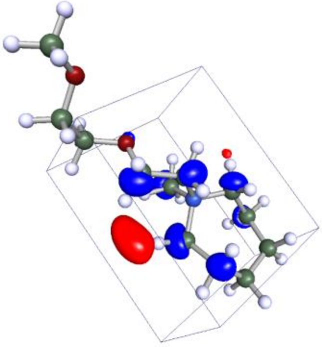	-2.980
7	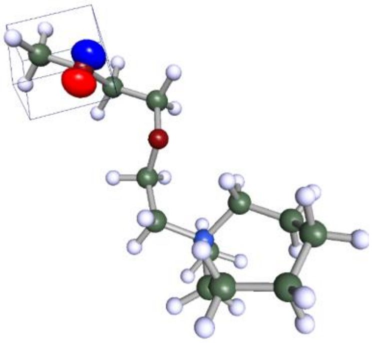	-9.554	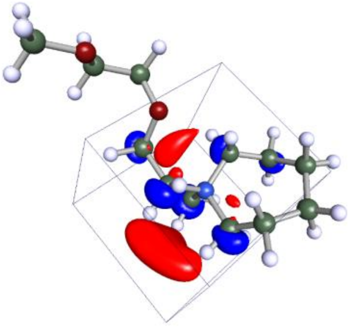	-3.067
8	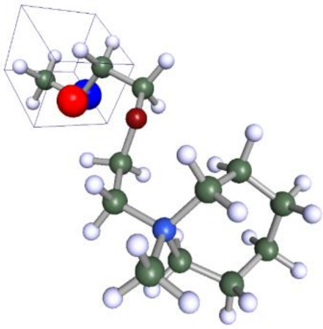	-9.747	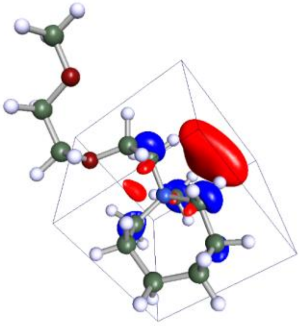	-3.004
9	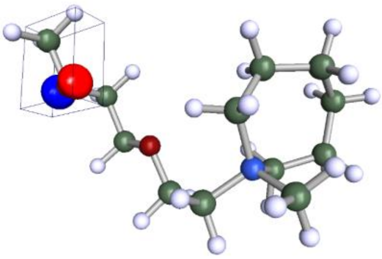	-9.562	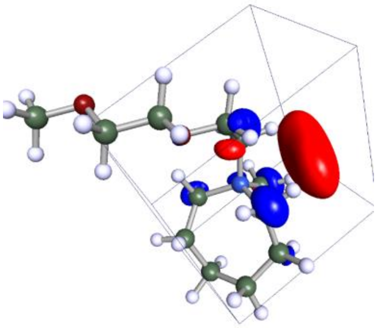	-3.069

Table S 9. *Continued...*

$[Aze_{1(2o2o1)}]^+$				
	HOMO		LUMO	
Conformer	Surface	Energy (eV)	Surface	Energy (eV)
10	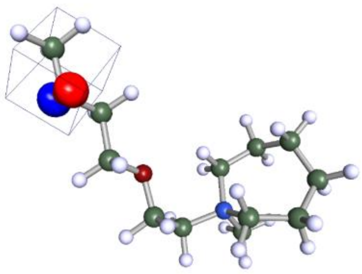	-9.516	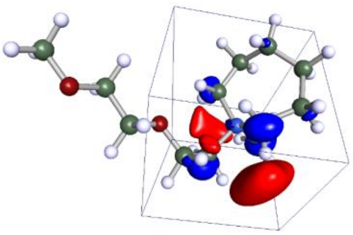	-3.244
Average		-9.544		-3.040

$[Aze_{(2o1)2}]^+$				
	HOMO		LUMO	
Conformer	Surface	Energy (eV)	Surface	Energy (eV)
1	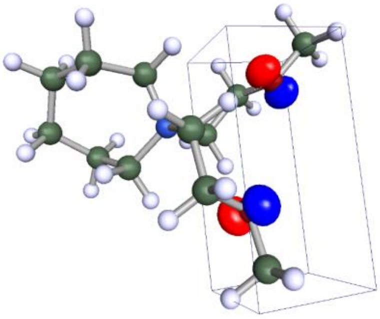	-10.599	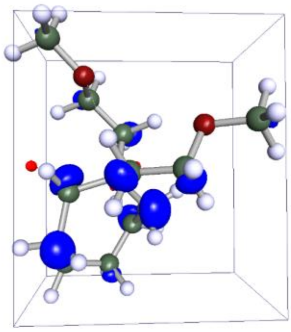	-2.718
2	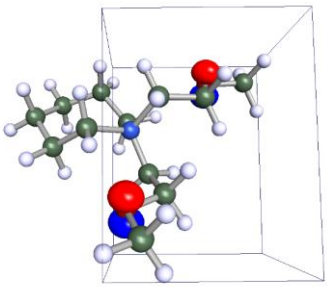	-10.716	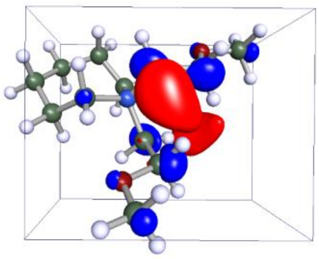	-2.841

Table S 9. *Continued...*

	$[Aze_{(2o1)2}]^{+}$			
	HOMO		LUMO	
Conformer	Surface	Energy (eV)	Surface	Energy (eV)
3	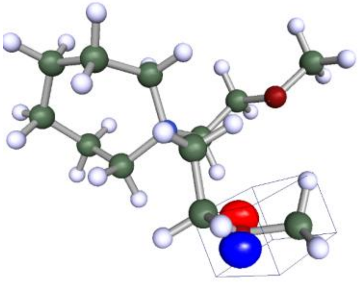	-10.534	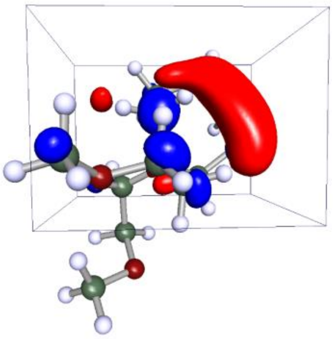	-2.767
4	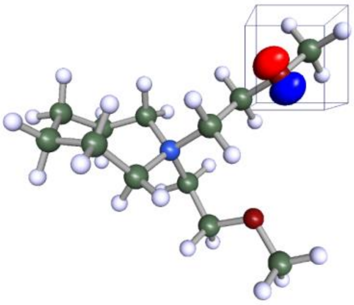	-10.305	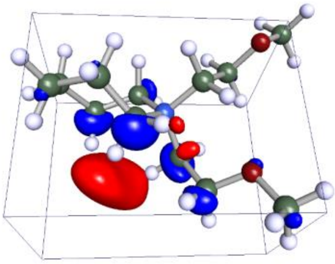	-2.863
5	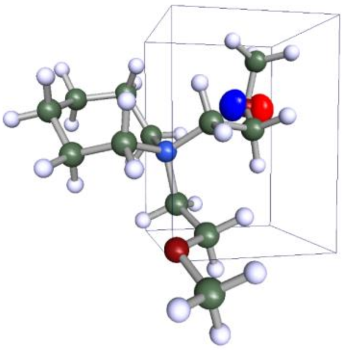	-10.792	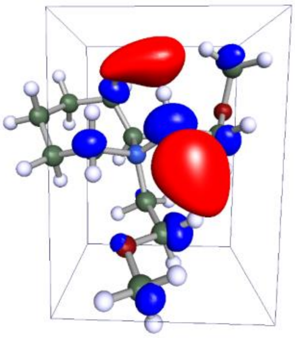	-2.849
6	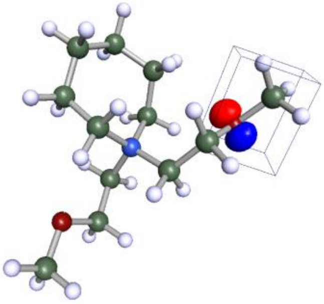	-10.694	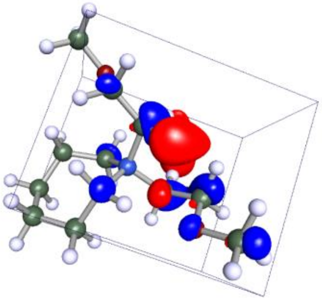	-2.767

Table S 9. *Continued...*

	$[Aze_{(2o1)2}]^+$			
	HOMO		LUMO	
Conformer	Surface	Energy (eV)	Surface	Energy (eV)
7	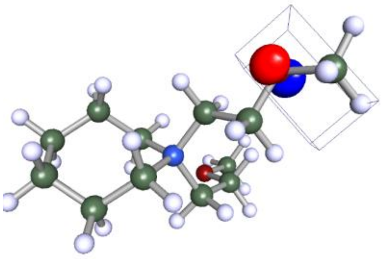	-10.558	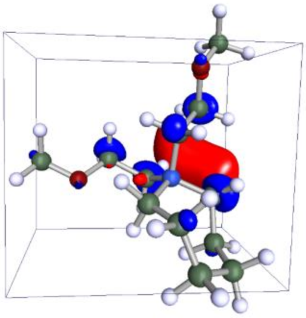	-2.874
8	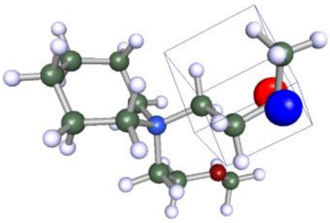	-10.417	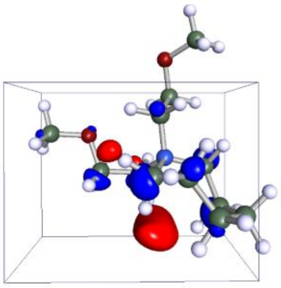	-2.901
9	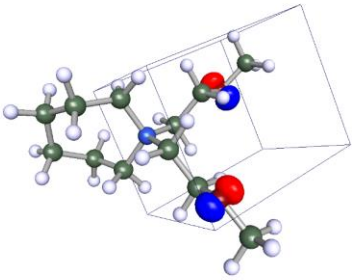	-10.555	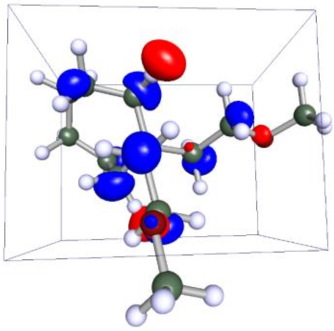	-2.874
10	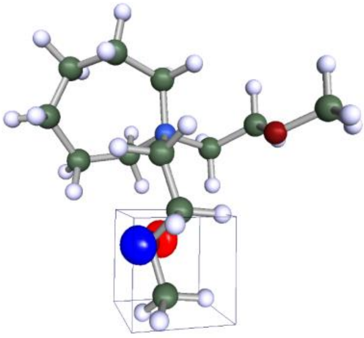	-10.574	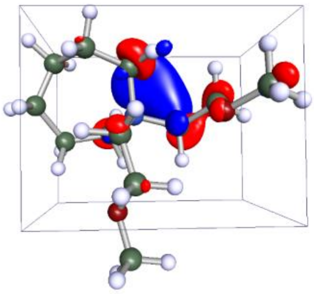	-2.901
Average		-10.574		-2.835

Table S 9. *Continued...*

	$[Aze_{(2o2o1)2}]^+$			
	HOMO		LUMO	
Conformer	Surface	Energy (eV)	Surface	Energy (eV)
1	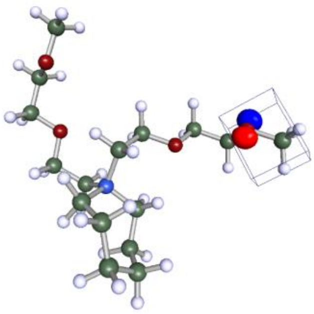	-9.287	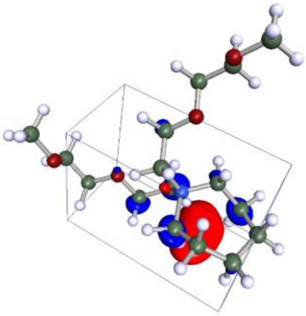	-2.680
2	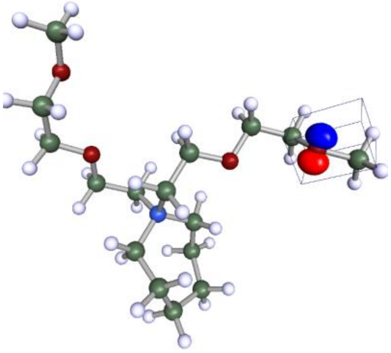	-9.306	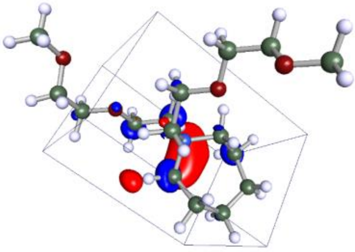	-2.710
3	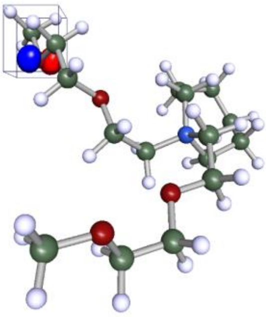	-9.309	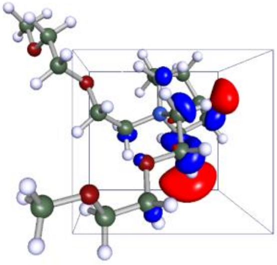	-2.762
4	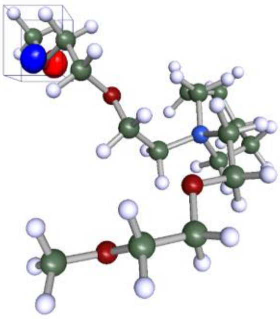	-9.274	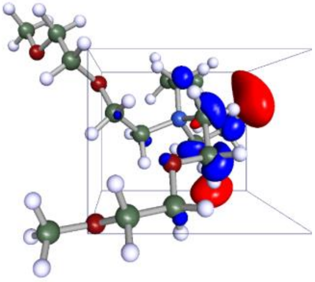	-2.708

Table S 9. *Continued...*

$[Aze_{(2o2o1)2}]^{+}$				
	HOMO		LUMO	
Conformer	Surface	Energy (eV)	Surface	Energy (eV)
5	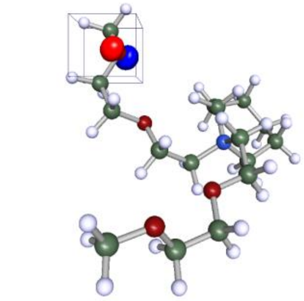	-9.440	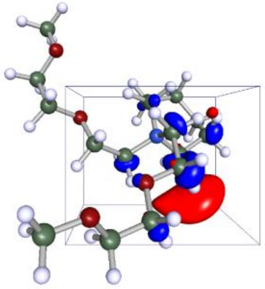	-2.716
6	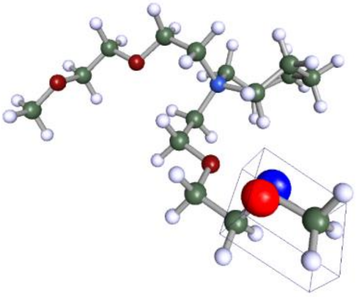	-9.434	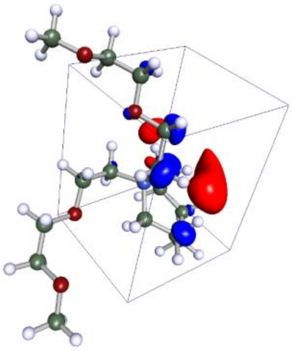	-2.640
7	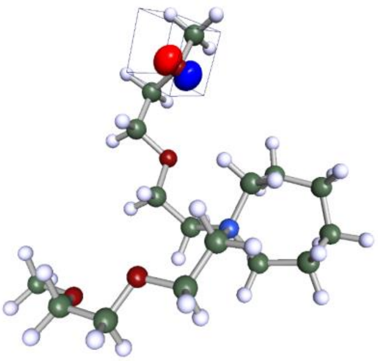	-9.429	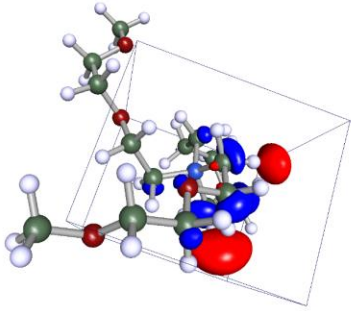	-2.642
8	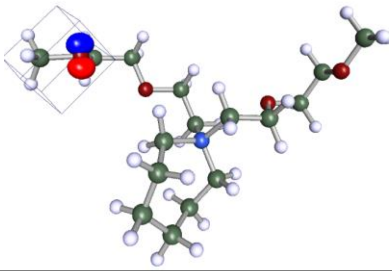	-9.426	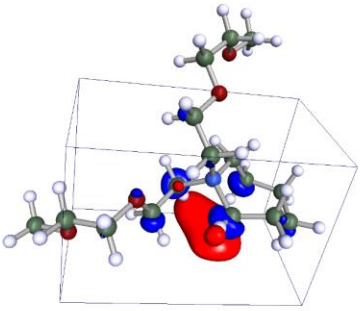	-2.506

Table S 9. *Continued...*

		$[Aze_{(2o2o1)2}]^+$		
	HOMO		LUMO	
Conformer	Surface	Energy (eV)	Surface	Energy (eV)
9	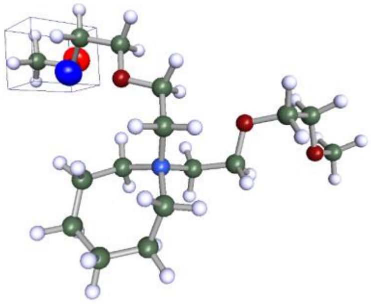	-9.423	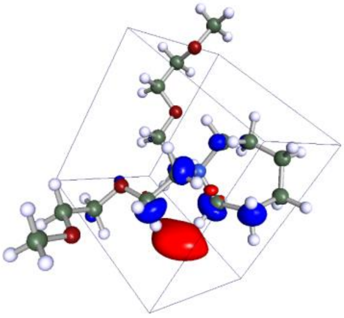	-2.571
10	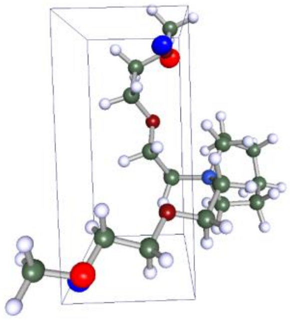	-9.483	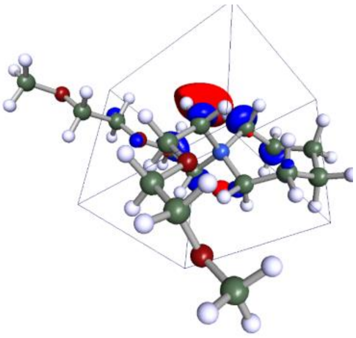	-2.754
Average		-9.381		-2.669

Table S 9. *Continued...*

[TFSI]⁻				
	HOMO		LUMO	
Conformer	Surface	Energy (eV)	Surface	Energy (eV)
trans	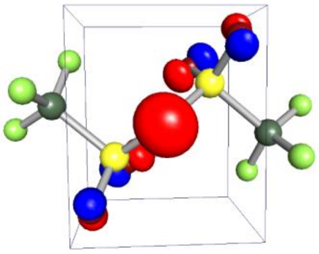	-4.188	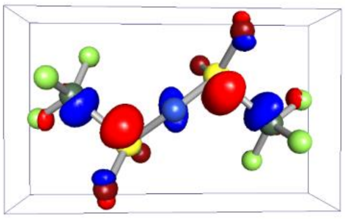	3.347
cis	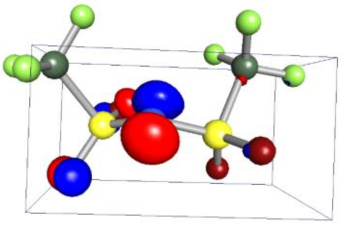	-3.967	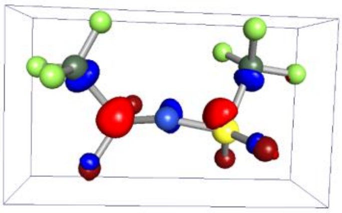	3.380
3	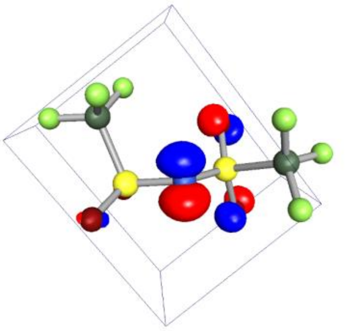	-3.889	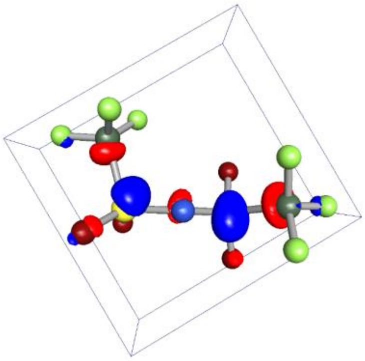	3.358
Average		-4.015		3.362

Table S8. *Continued...*

[$Pyrr_{14}$][TFSI]				
	HOMO		LUMO	
Conformer	Surface	Energy (eV)	Surface	Energy (eV)
trans	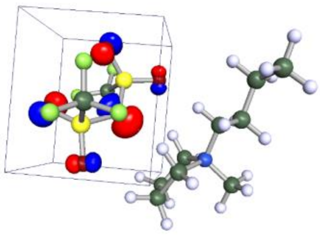	-6.626	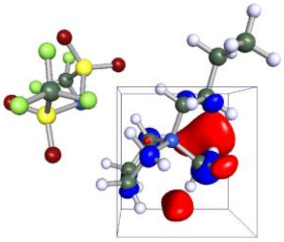	-1.186
Total energy = -2236.64899 Hartree				
cis	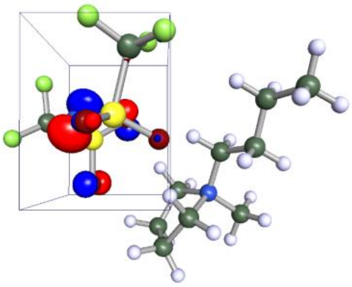	-6.348	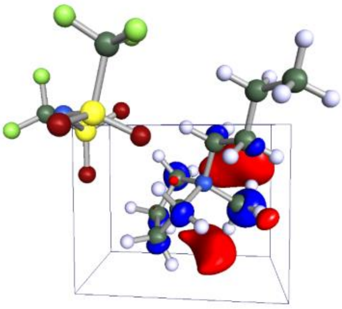	-1.369
Total energy = -2236.64616 Hartree				

Table S8. *Continued...*

$[Pyrr_{1(2o2o1)}][TFSI]$				
	HOMO		LUMO	
Conformer	Surface	Energy (eV)	Surface	Energy (eV)
trans	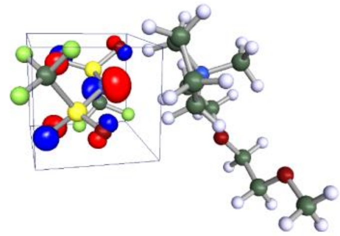	-6.471	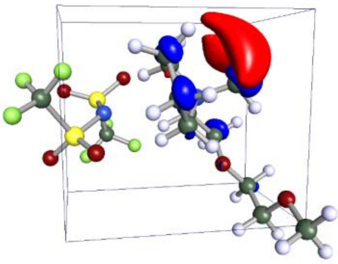	-1.140
Total energy = -2426.34916 Hartree				
cis	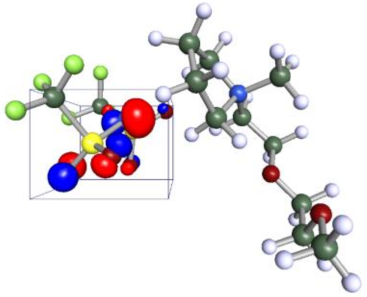	-6.191	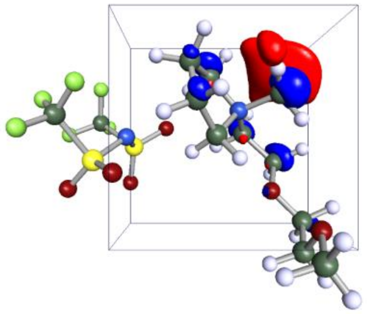	-1.325
Total energy = -2426.34638 Hartree				

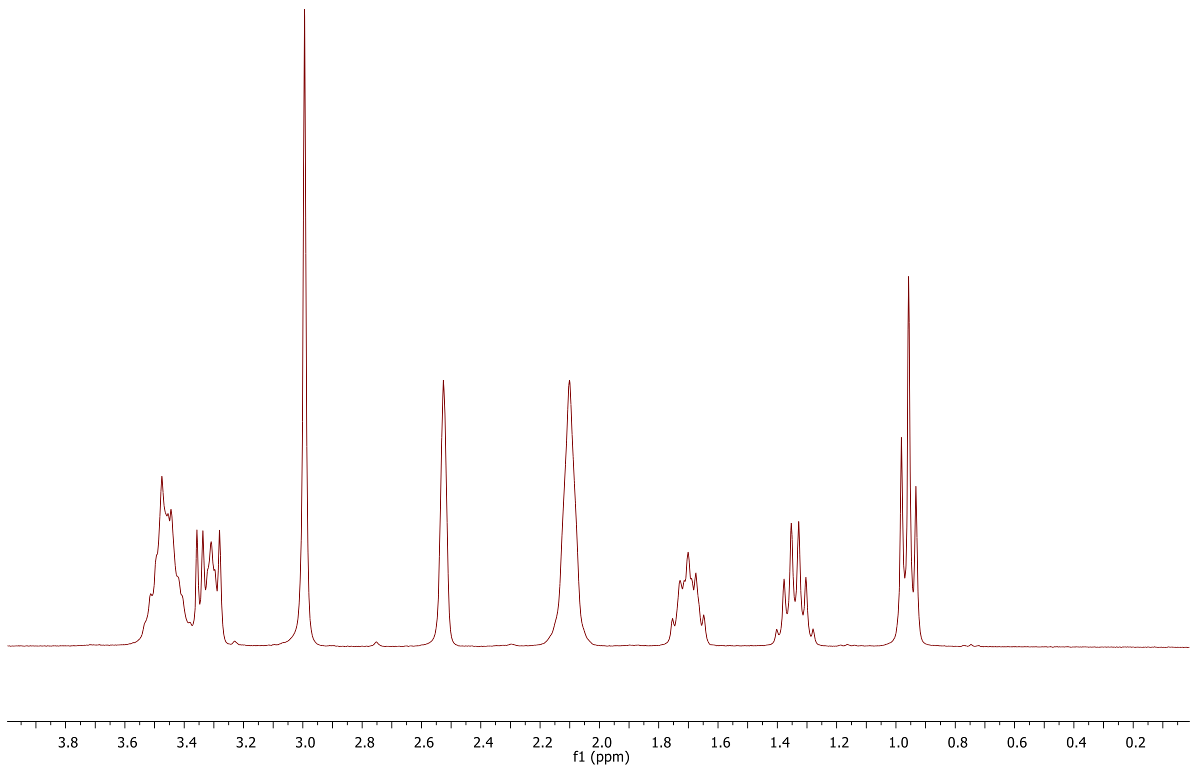

Figure S 7. ^{1}H NMR of [$Pyrr_{14}$][TFSI] in d_6-DMSO.

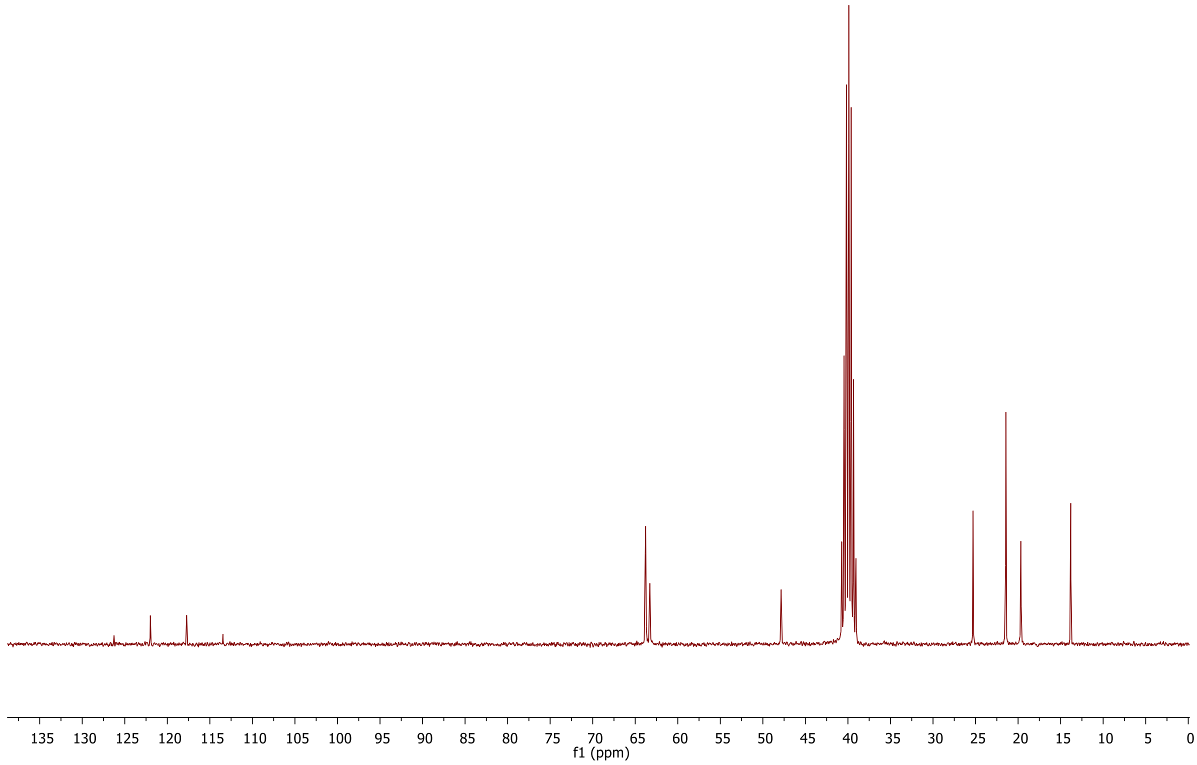

Figure S 8. ^{13}C NMR of [$Pyrr_{14}$][TFSI] in d_6-DMSO.

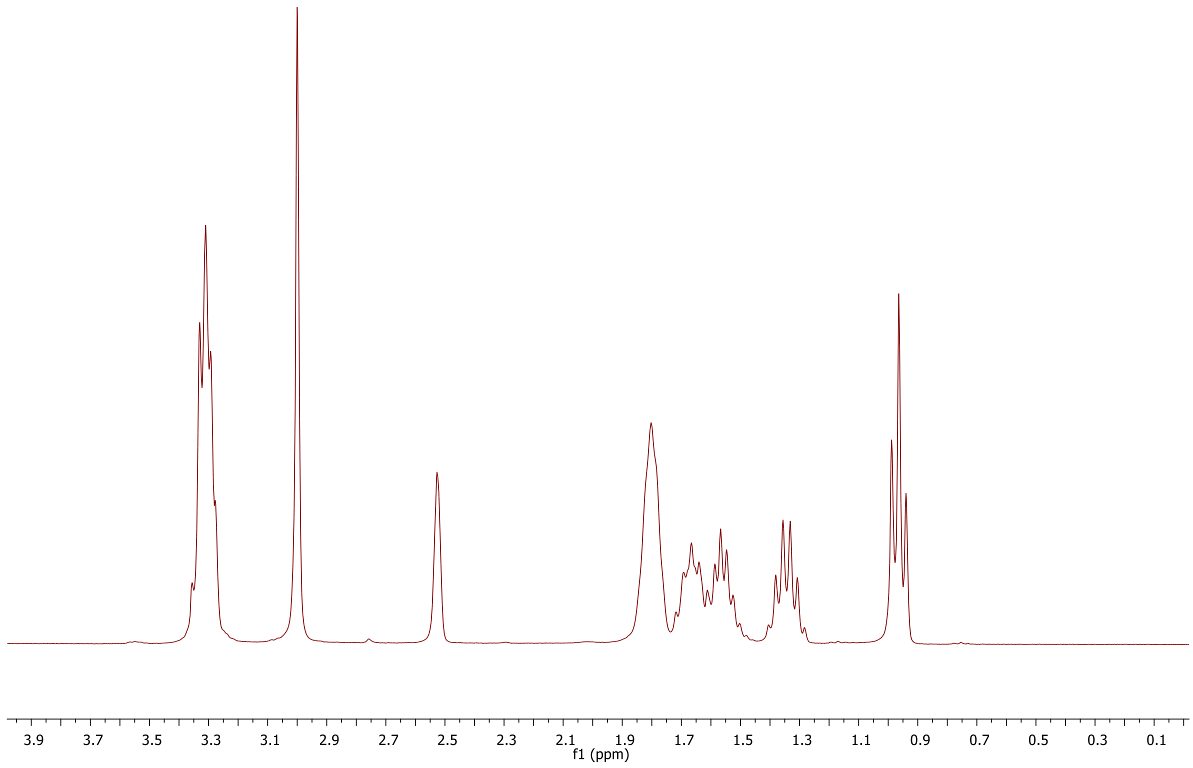

Figure S 9. ^{1}H NMR of [Pip$_{14}$][TFSI] in d$_6$-DMSO.

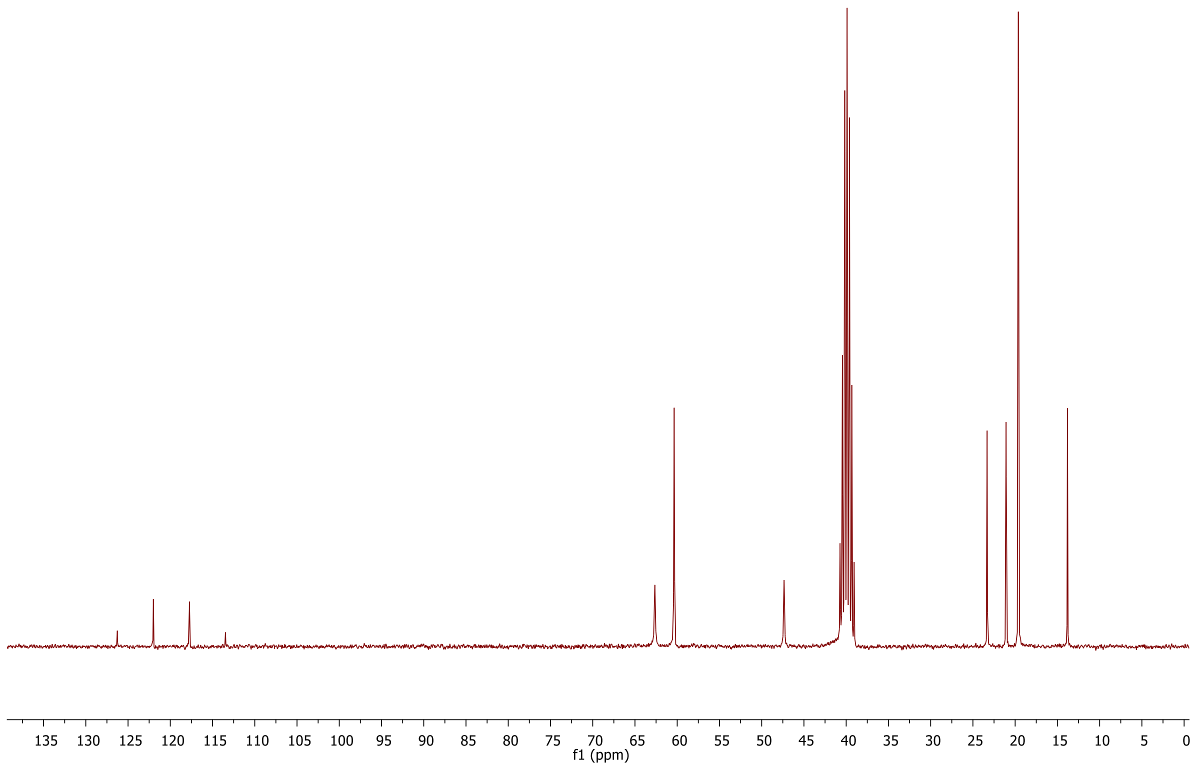

Figure S 10. ^{13}C NMR of [Pip$_{14}$][TFSI] in d$_6$-DMSO.

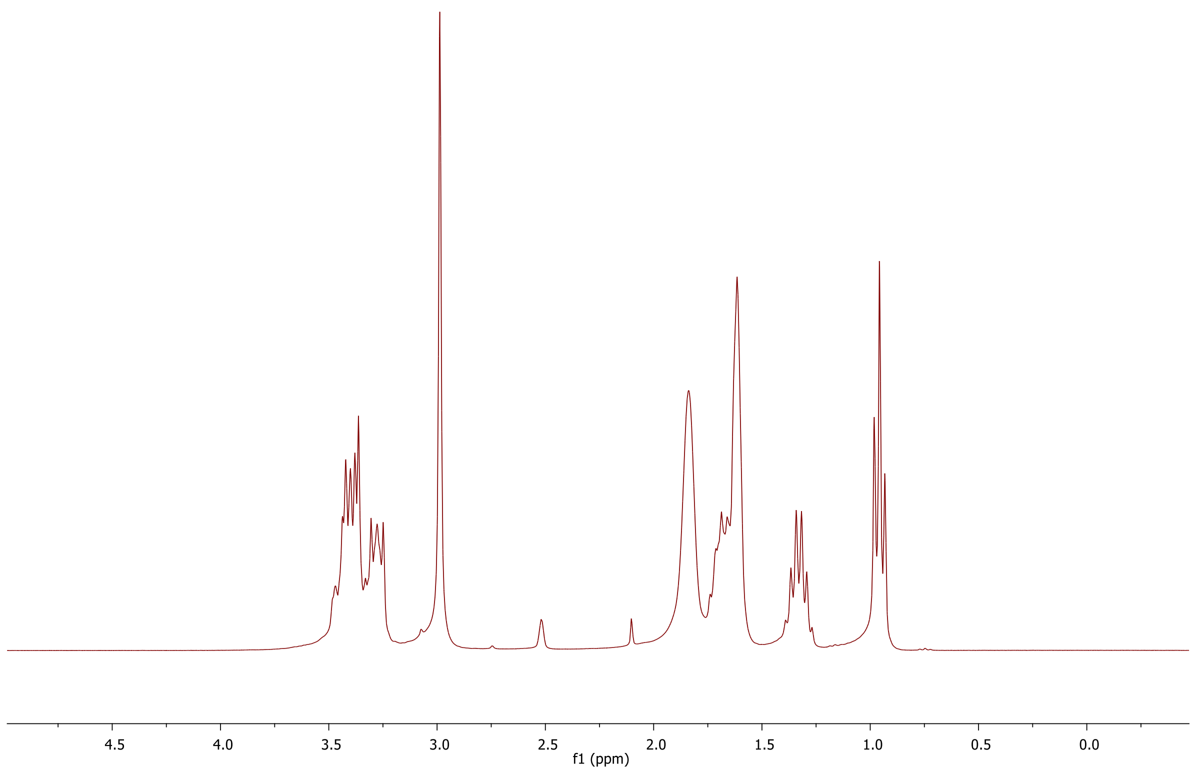

Figure S 11. ^{1}H NMR of [Aze$_{14}$][TFSI] in d_6-DMSO.

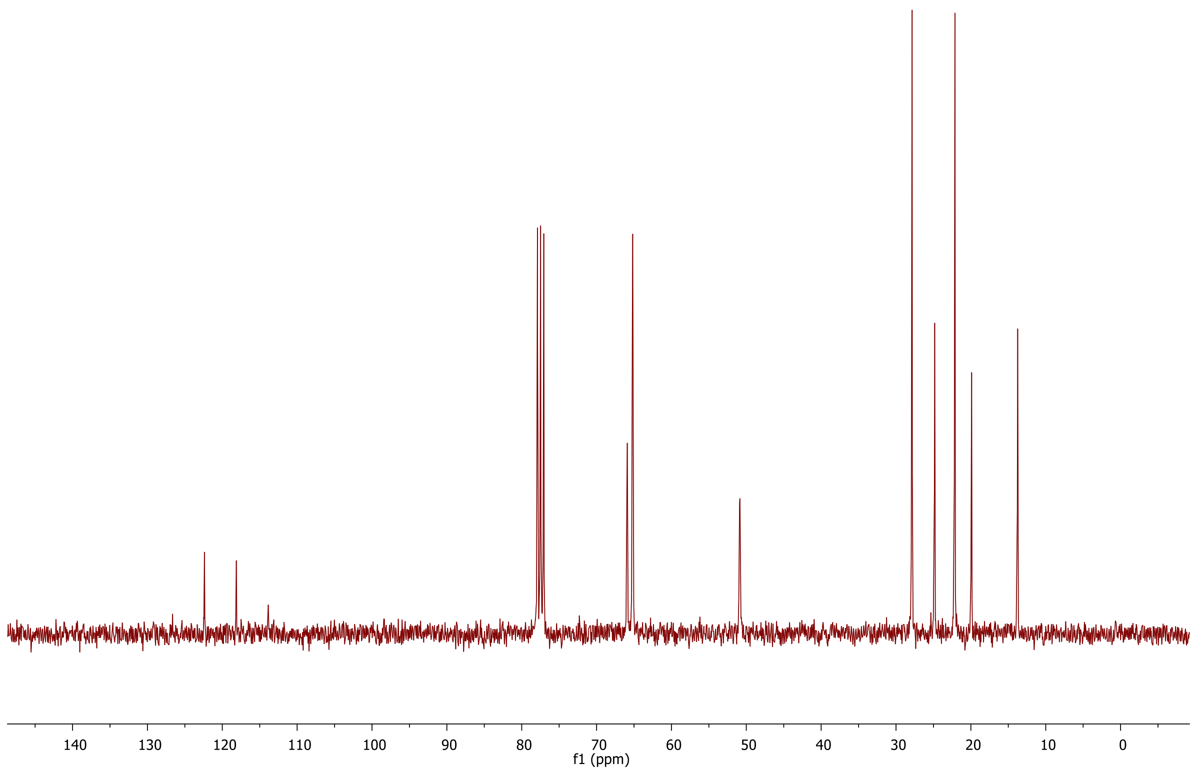

Figure S 12. ^{13}C NMR of [Aze$_{14}$][TFSI] in $CDCl_3$.

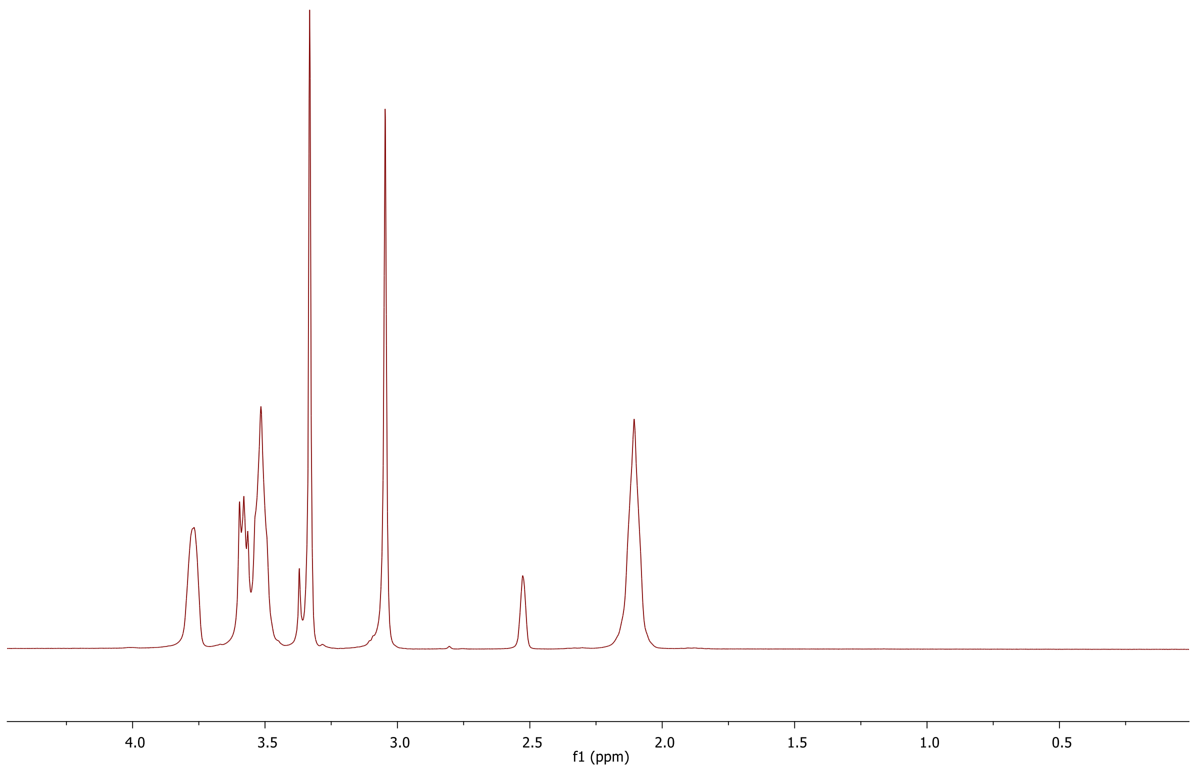

Figure S 13. ^{1}H NMR of [$Pyrr_{1(2o1)}$][TFSI] in d_6-DMSO.

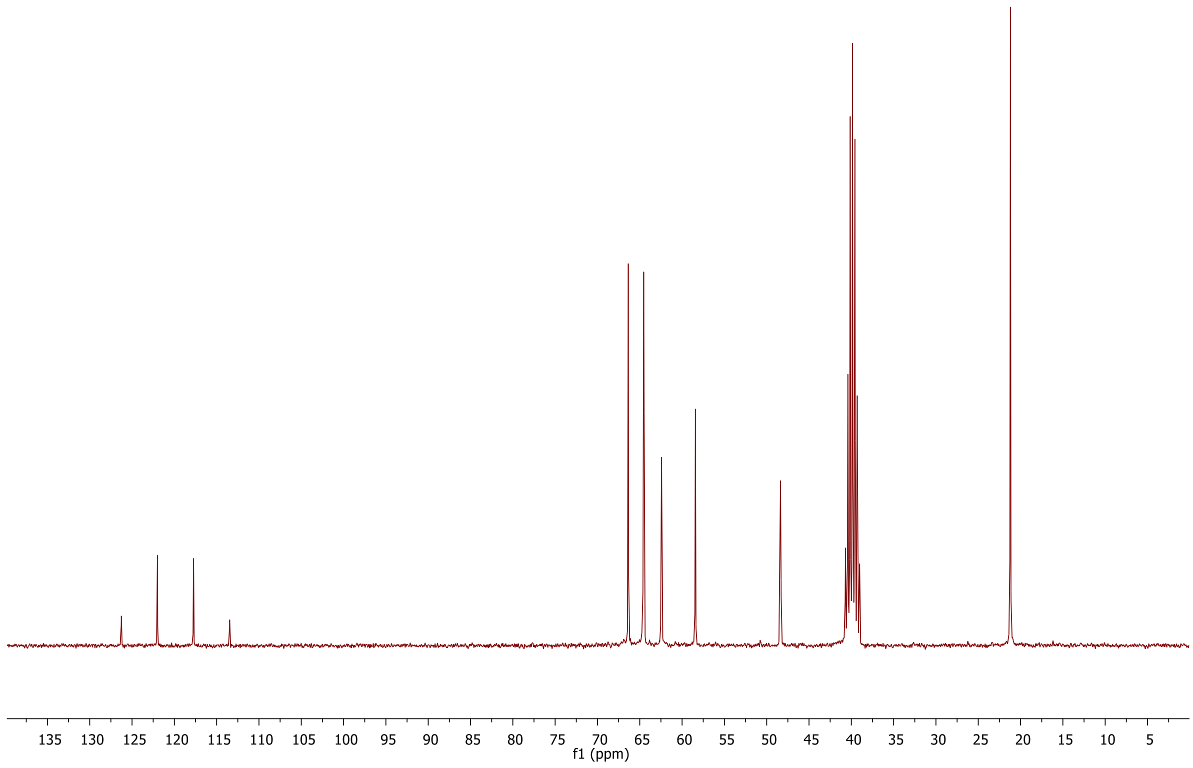

Figure S 14. ^{13}C NMR of [$Pyrr_{1(2o1)}$][TFSI] in d_6-DMSO.

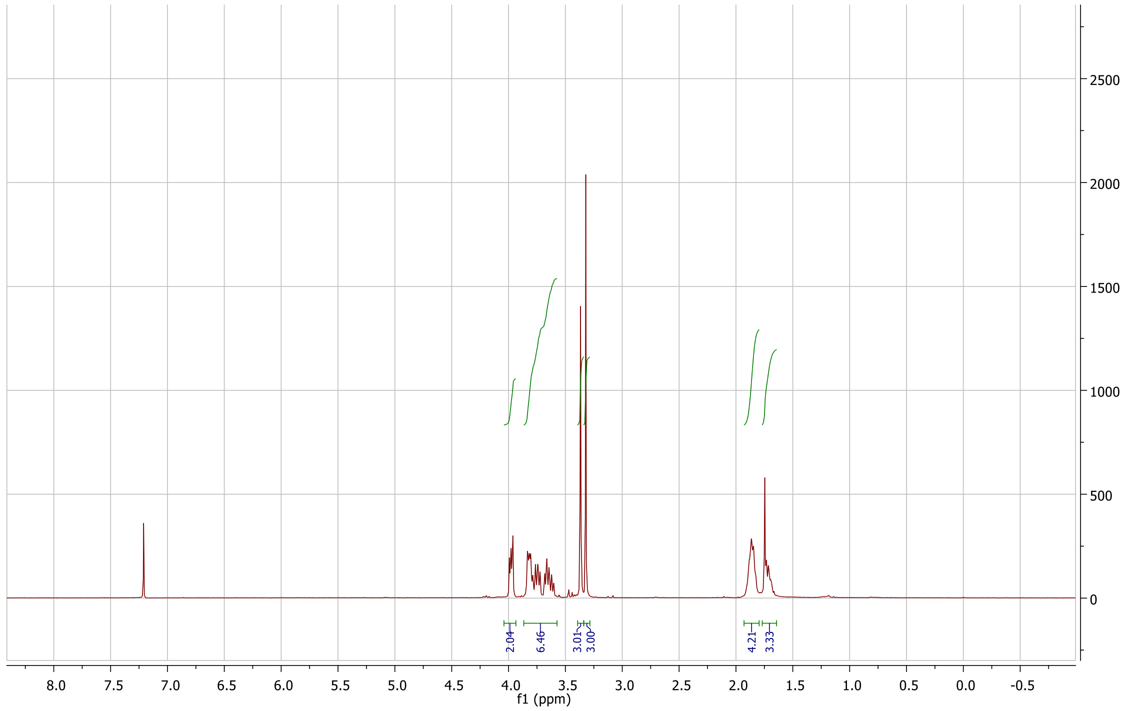

Figure S 15. ^{1}H NMR of $[Pip_{1(2o1)}][TFSI]$ in $CDCl_3$.

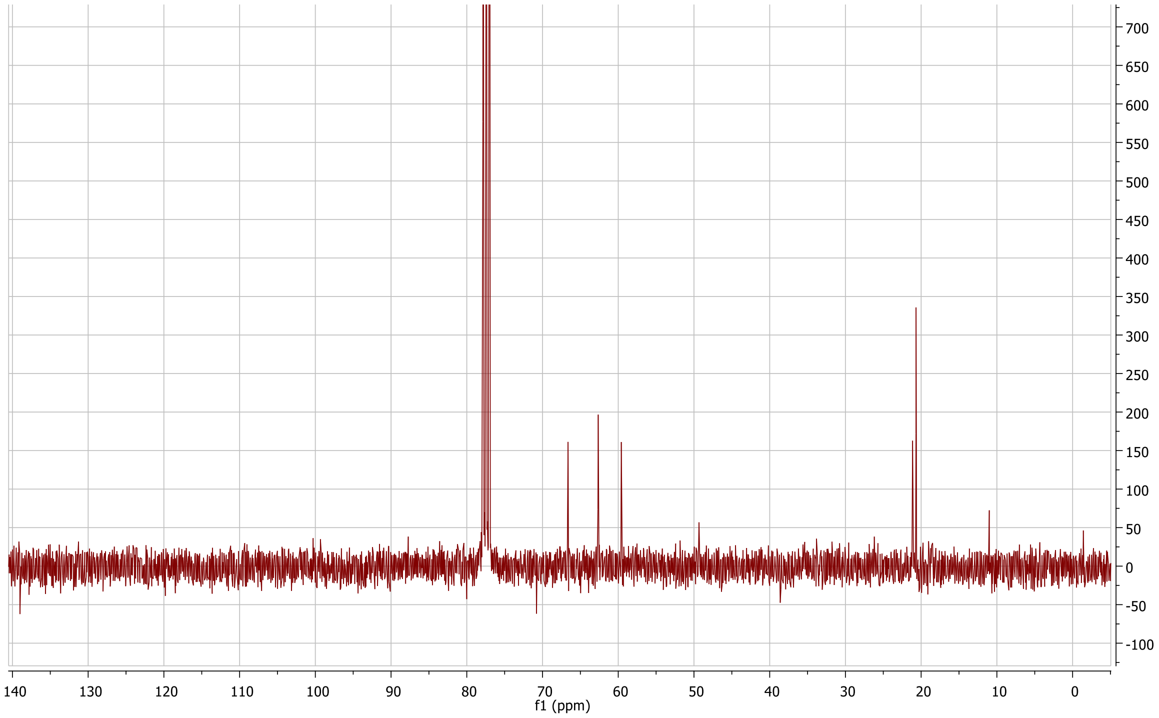

Figure S 16. ^{13}C NMR of $[Pip_{1(2o1)}][TFSI]$ in $CDCl_3$.

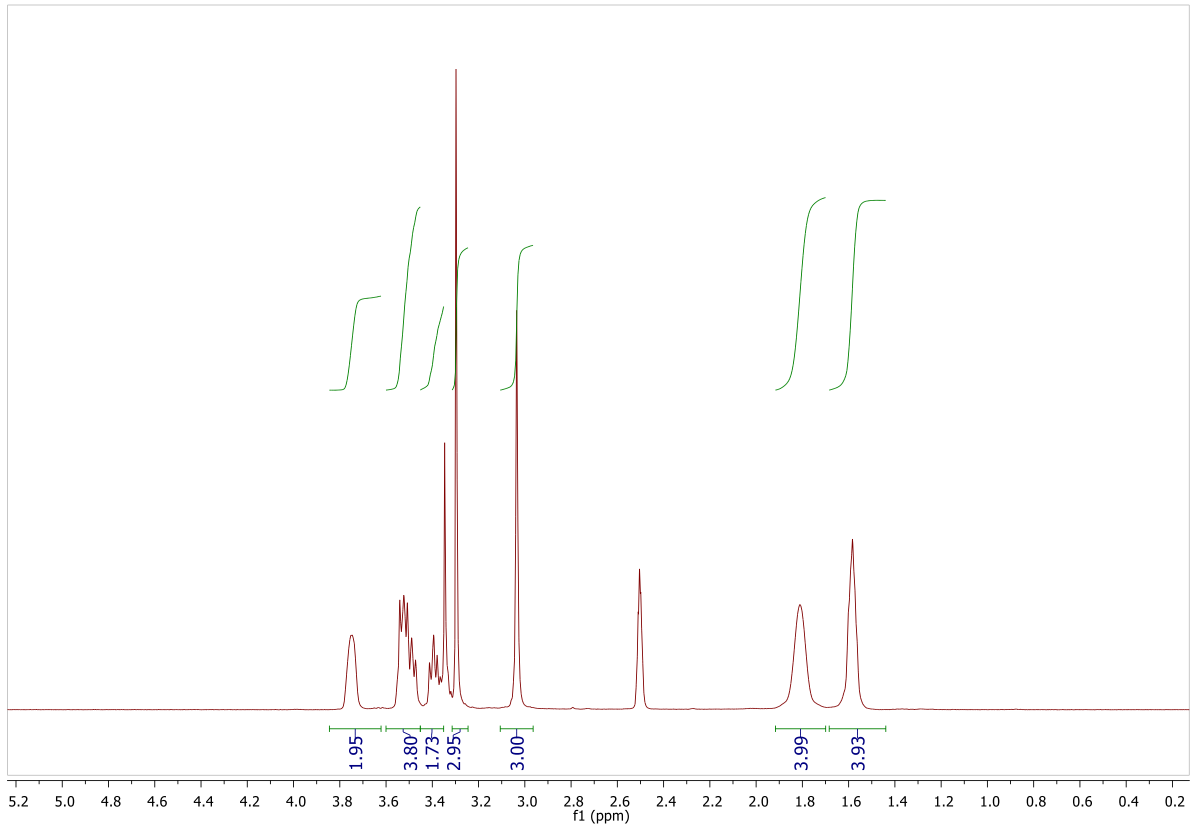

Figure S 17. ^{1}H NMR of [$Aze_{1(2o1)}$][TFSI] in d_6-DMSO.

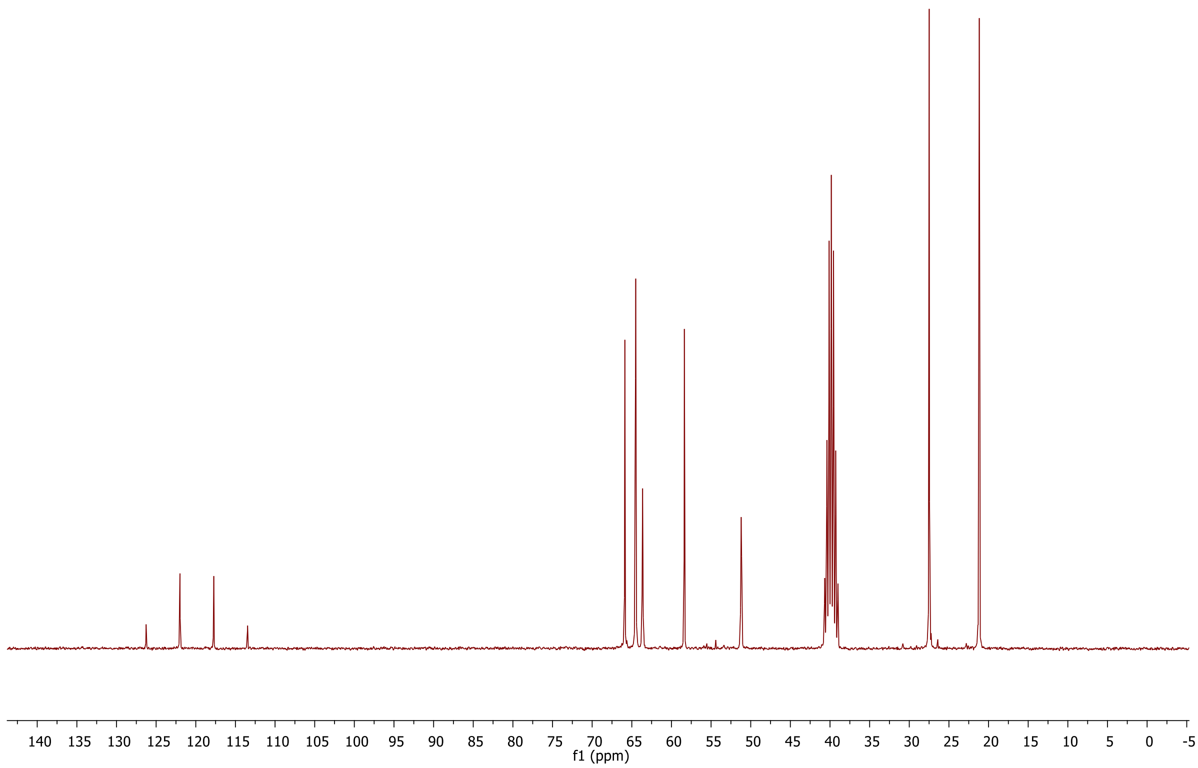

Figure S 18. ^{13}C NMR of [$Aze_{1(2o1)}$][TFSI] in d_6-DMSO.

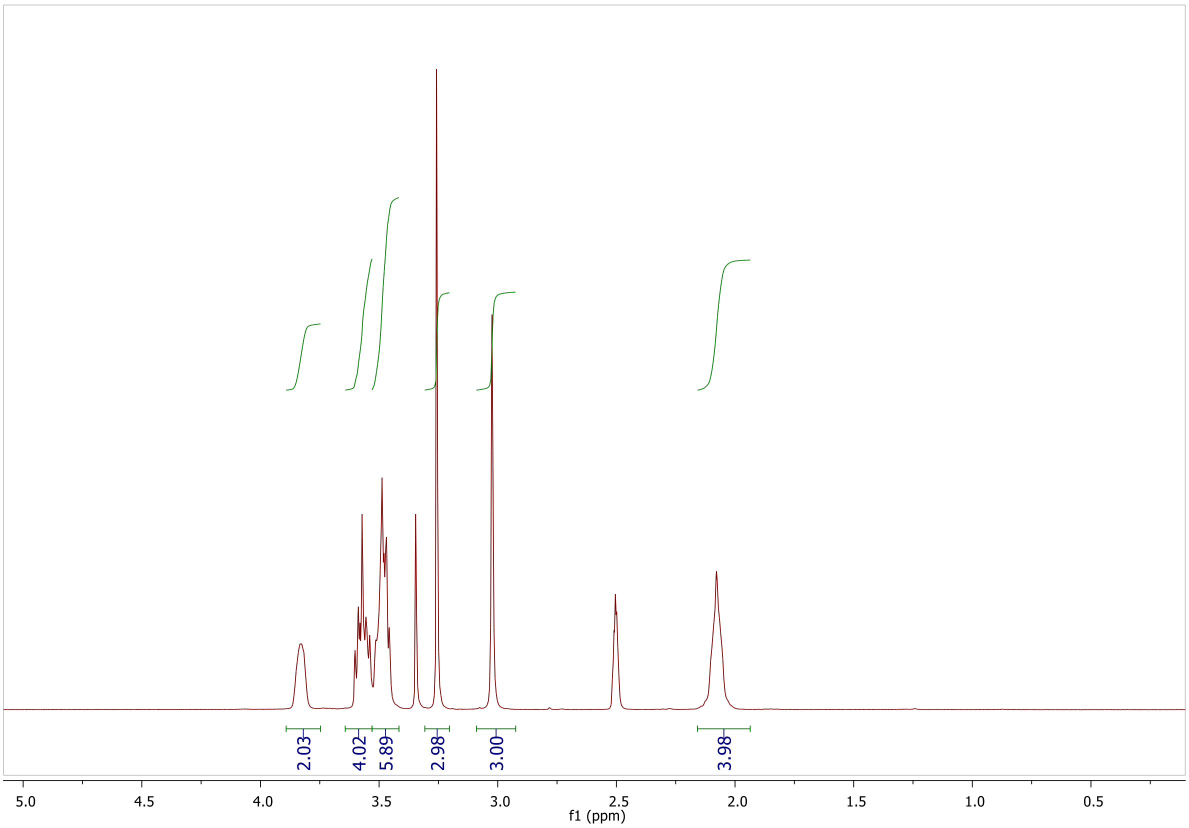

Figure S 19. ^{1}H NMR of [Pyrr$_{1(2o2o1)}$][TFSI] in d_6-DMSO.

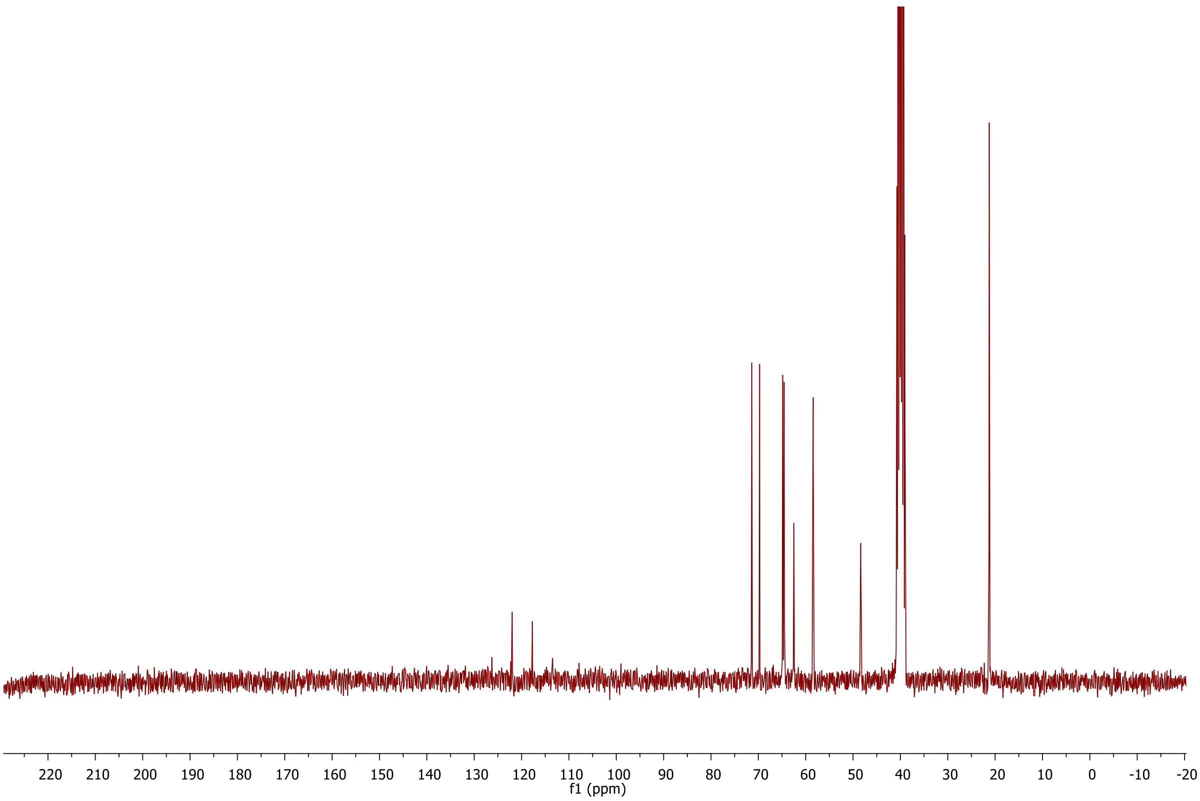

Figure S 20. ^{13}C NMR of [Pyrr$_{1(2o2o1)}$][TFSI] in d_6-DMSO.

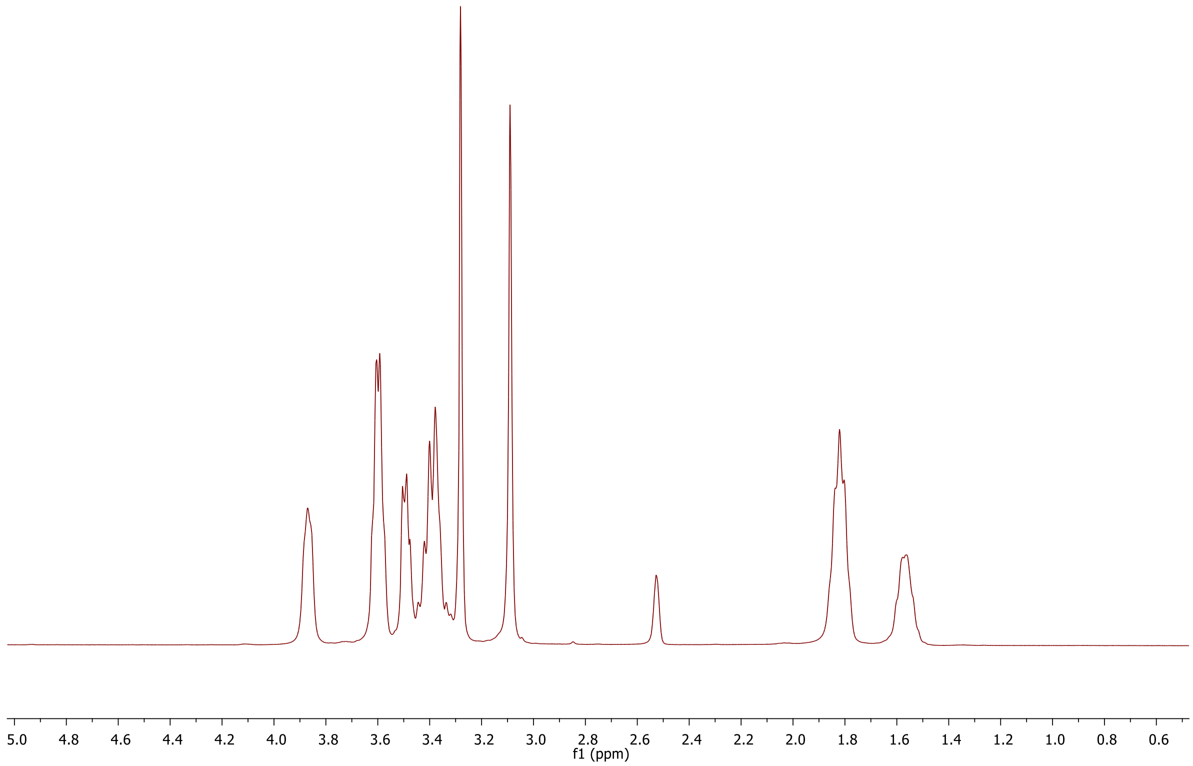

Figure S 21. ^{1}H NMR of $[Pip_{1(2o2o1)}]$[TFSI] in d_6-DMSO.

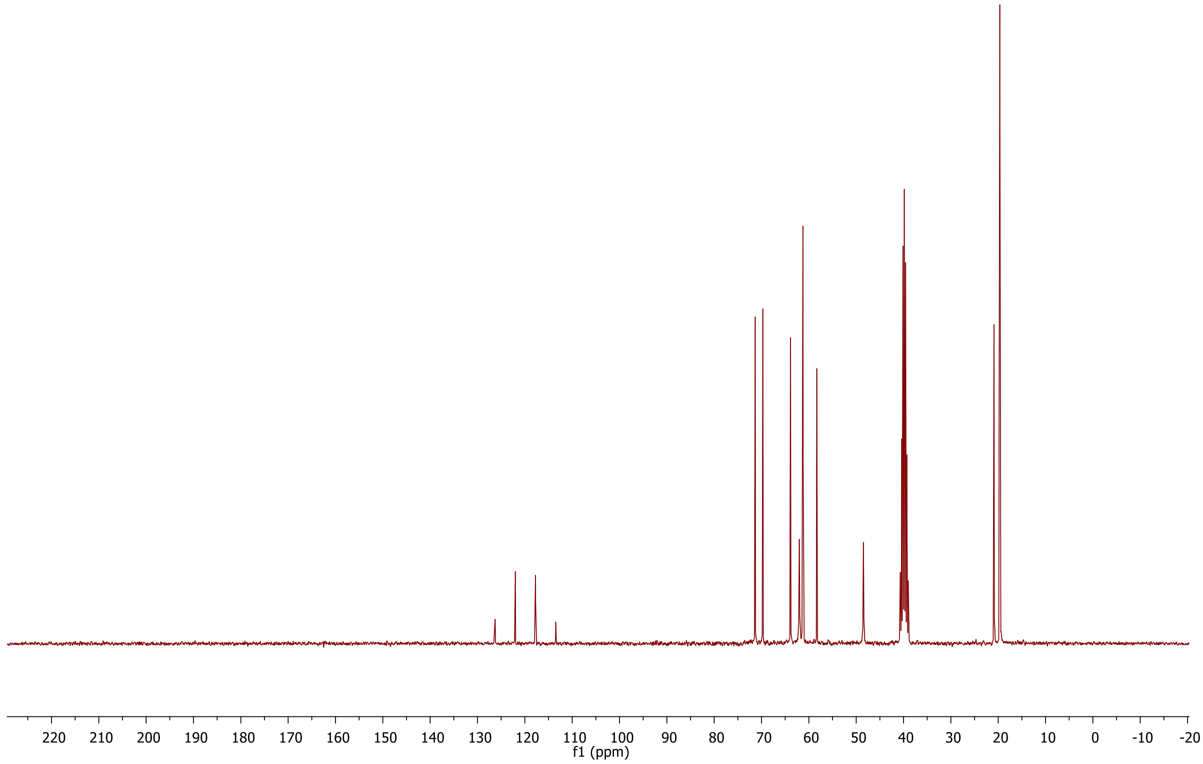

Figure S 22. ^{13}C NMR of $[Pip_{1(2o2o1)}]$[TFSI] in d_6-DMSO.

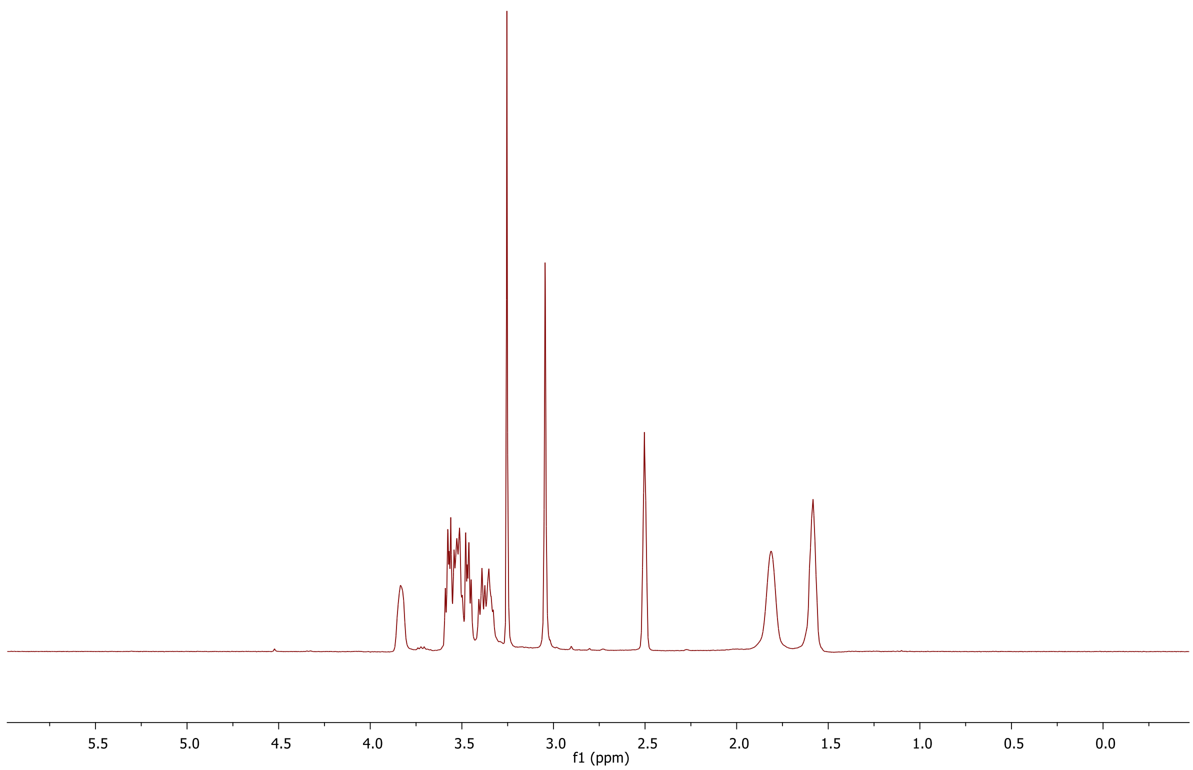

Figure S 23. ^{1}H NMR of [$Aze_{1(2o2o1)}$][TFSI] in d_6-DMSO.

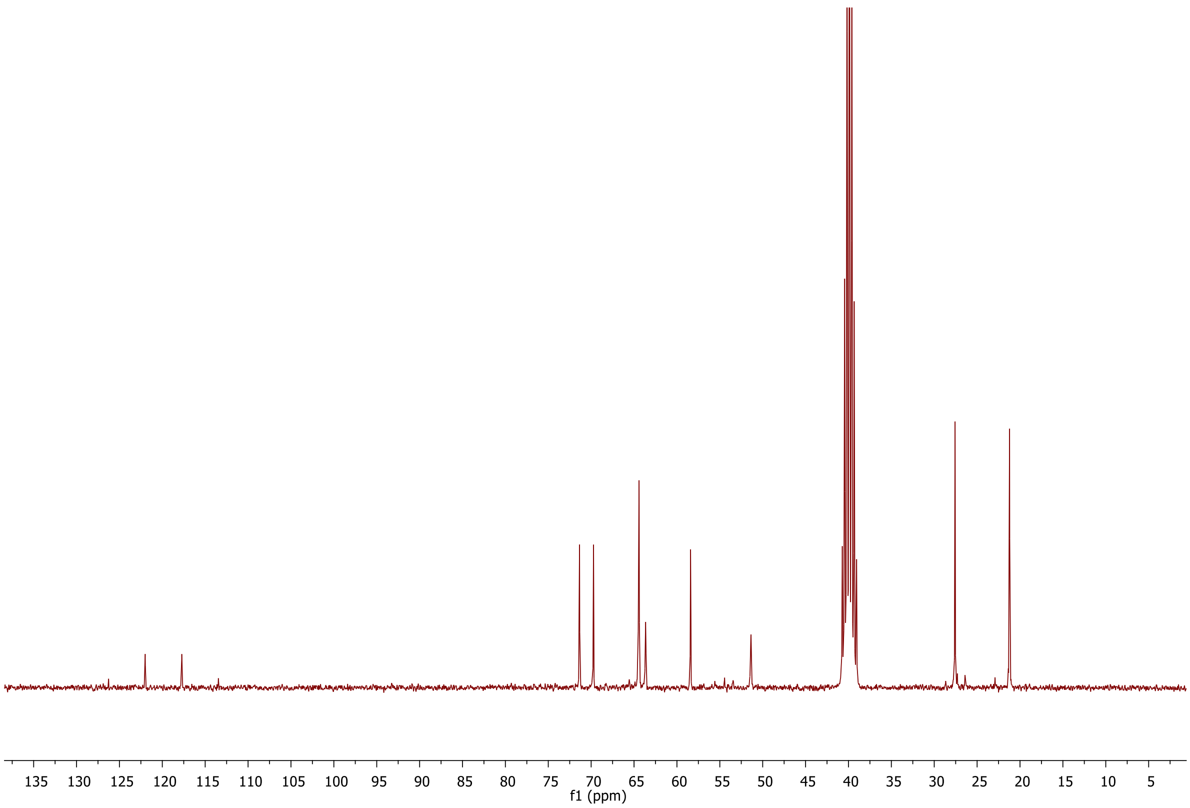

Figure S 24. ^{13}C NMR of [$Aze_{1(2o2o1)}$][TFSI] in d_6-DMSO.

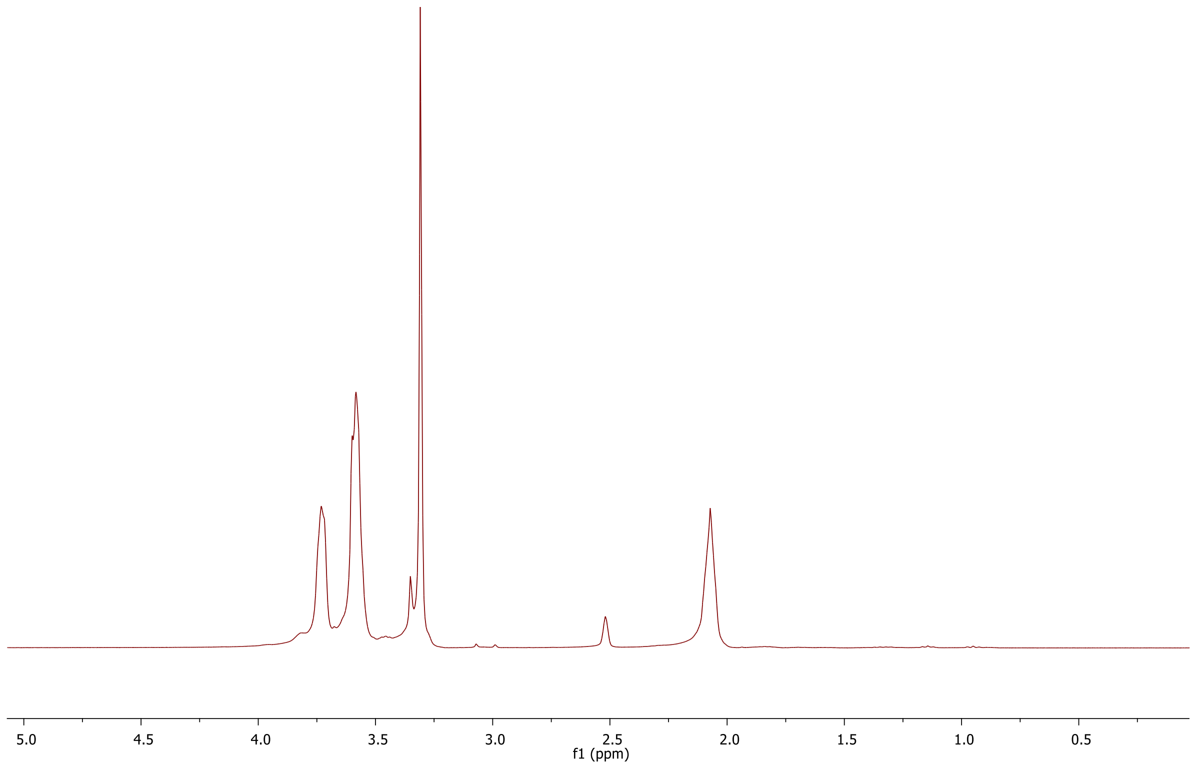

Figure S 25. ^{1}H NMR of $[Pyrr_{(2o1)2}][TFSI]$ in d_6-DMSO.

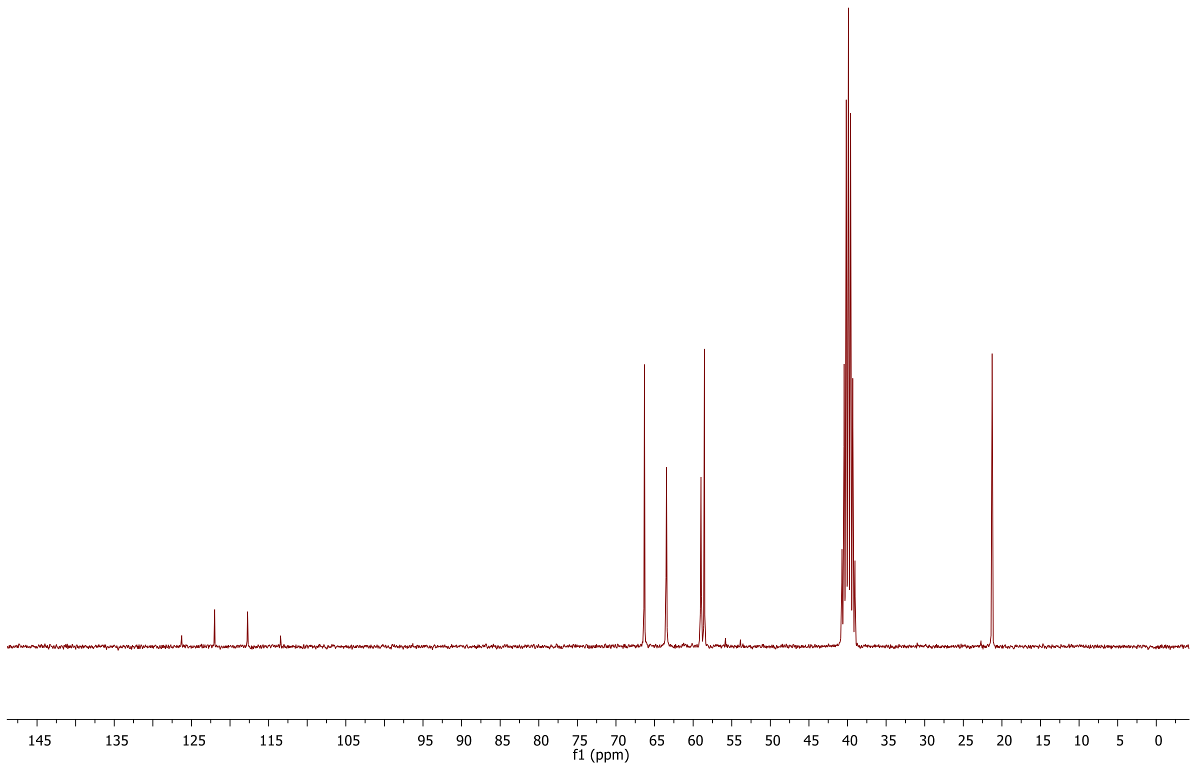

Figure S 26. ^{13}C NMR of $[Pyrr_{(2o1)2}][TFSI]$ in d_6-DMSO.

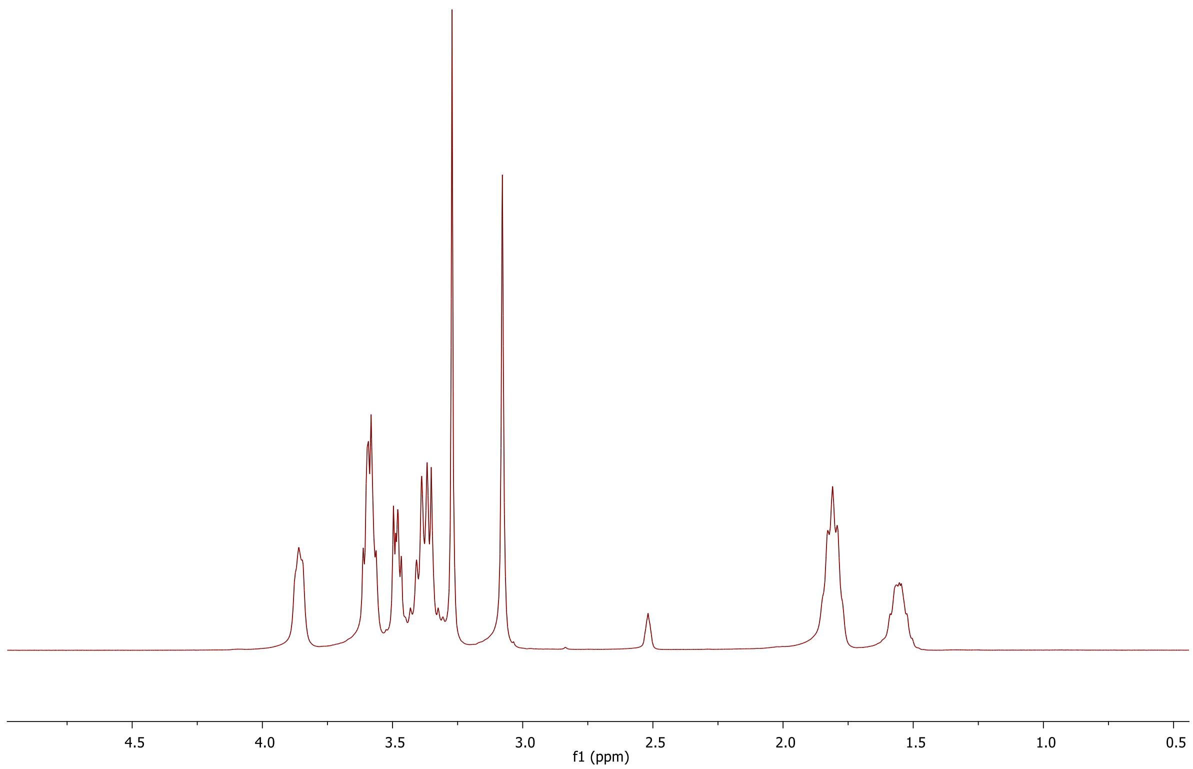

Figure S 27. ^{1}H NMR of $[Pip_{(2o1)2}][TFSI]$ in d_6-DMSO.

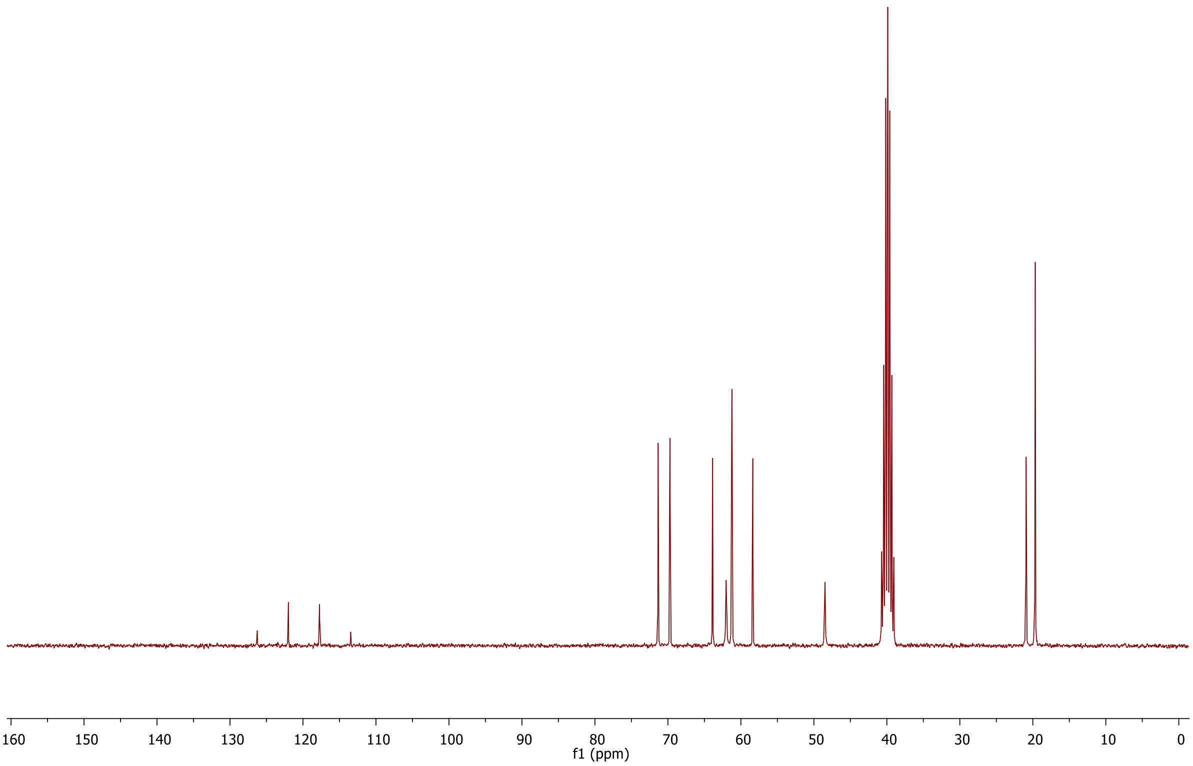

Figure S 28. ^{13}C NMR of $[Pip_{(2o1)2}][TFSI]$ in d_6-DMSO.

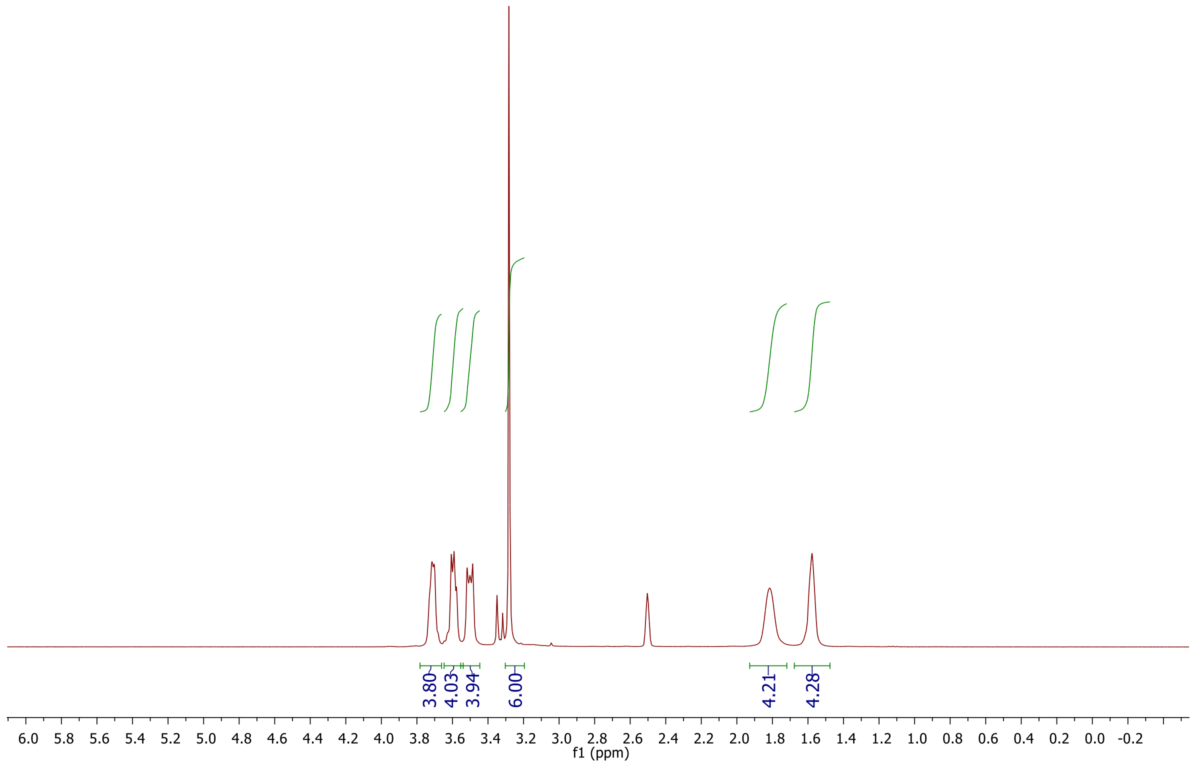

Figure S 29. ^{1}H NMR of $[Aze_{(2o1)2}]$[TFSI] in d_6-DMSO.

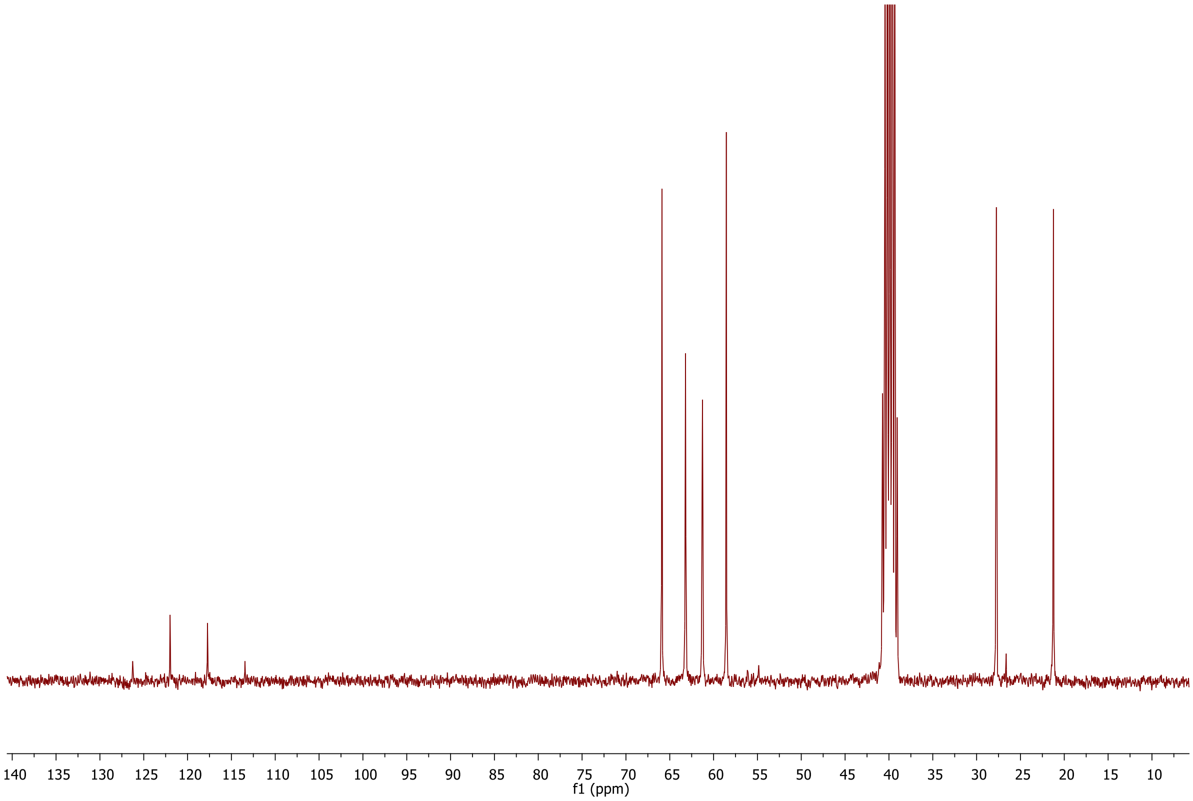

Figure S 30. ^{13}C NMR of $[Aze_{(2o1)2}]$[TFSI] in d_6-DMSO.

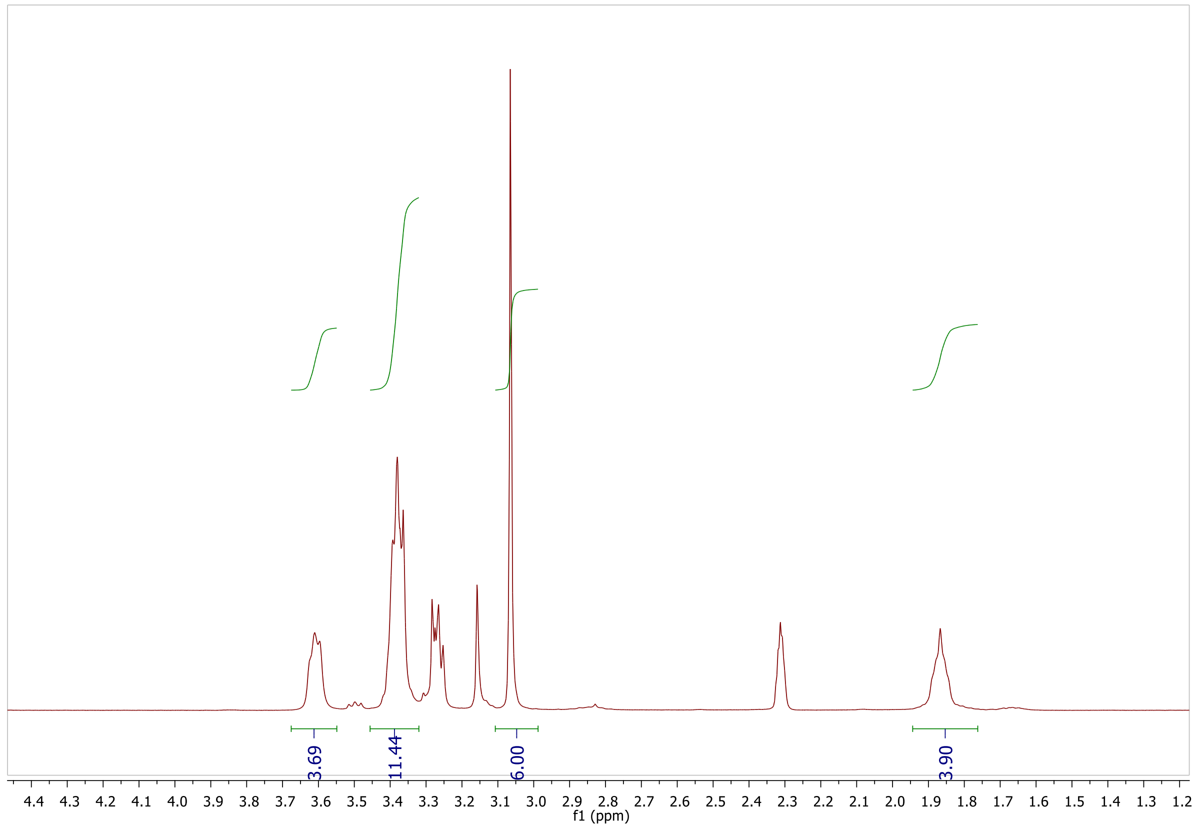

Figure S 31. ^{1}H NMR of [Pyrr$_{(2o2o1)2}$][TFSI] in d$_6$-DMSO.

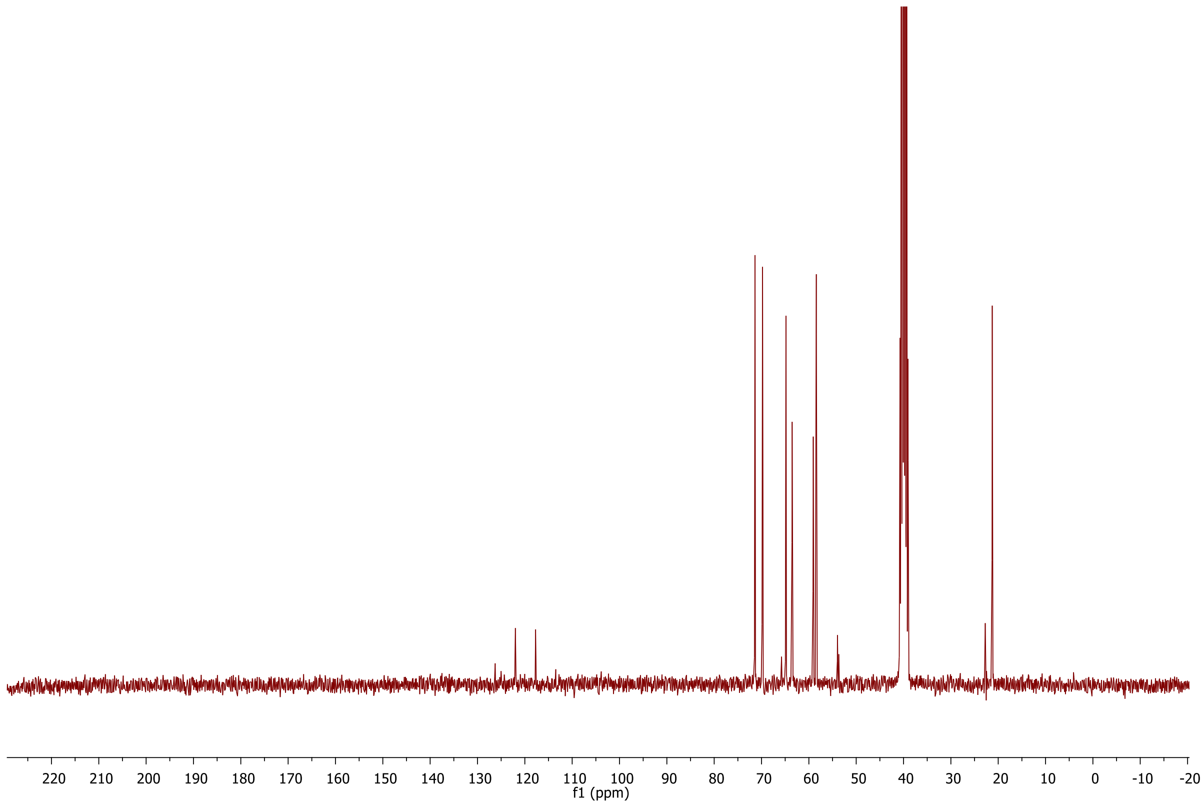

Figure S 32. ^{13}C NMR of [Pyrr$_{(2o2o1)2}$][TFSI] in d$_6$-DMSO.

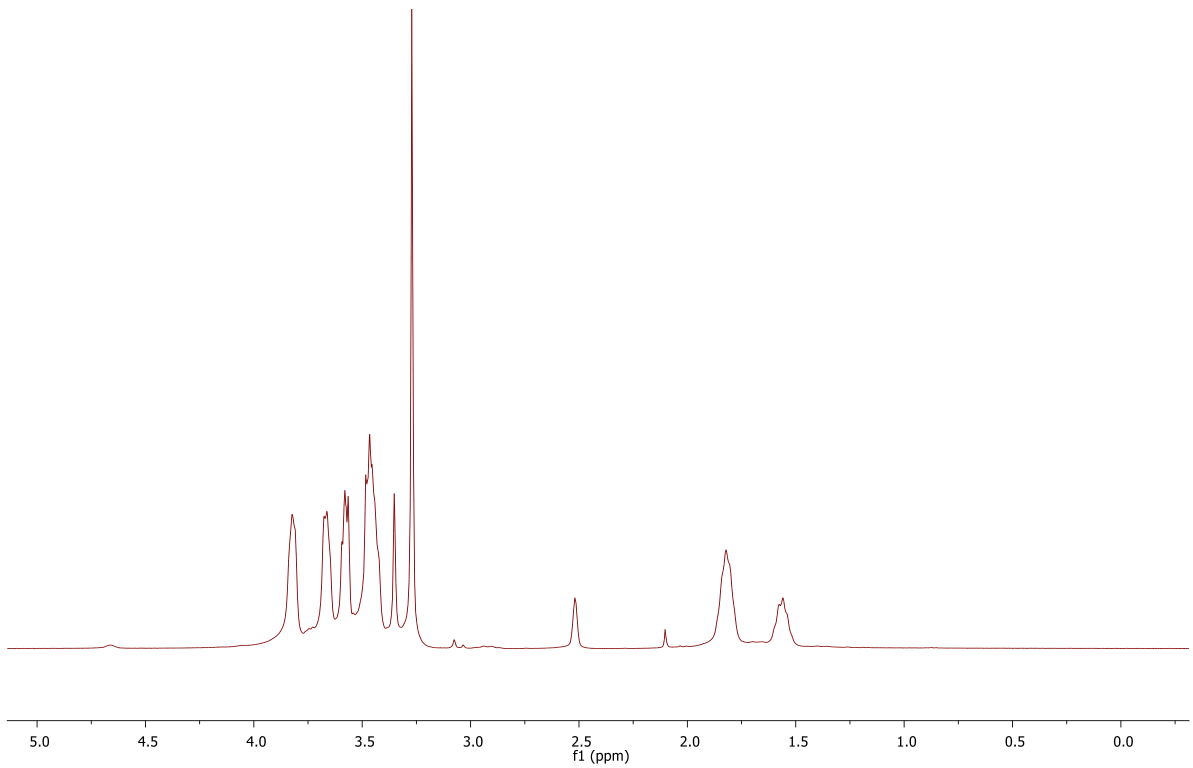

Figure S 33. ^{1}H NMR of $[Pip_{(2o2o1)2}][TFSI]$ in d_6-DMSO.

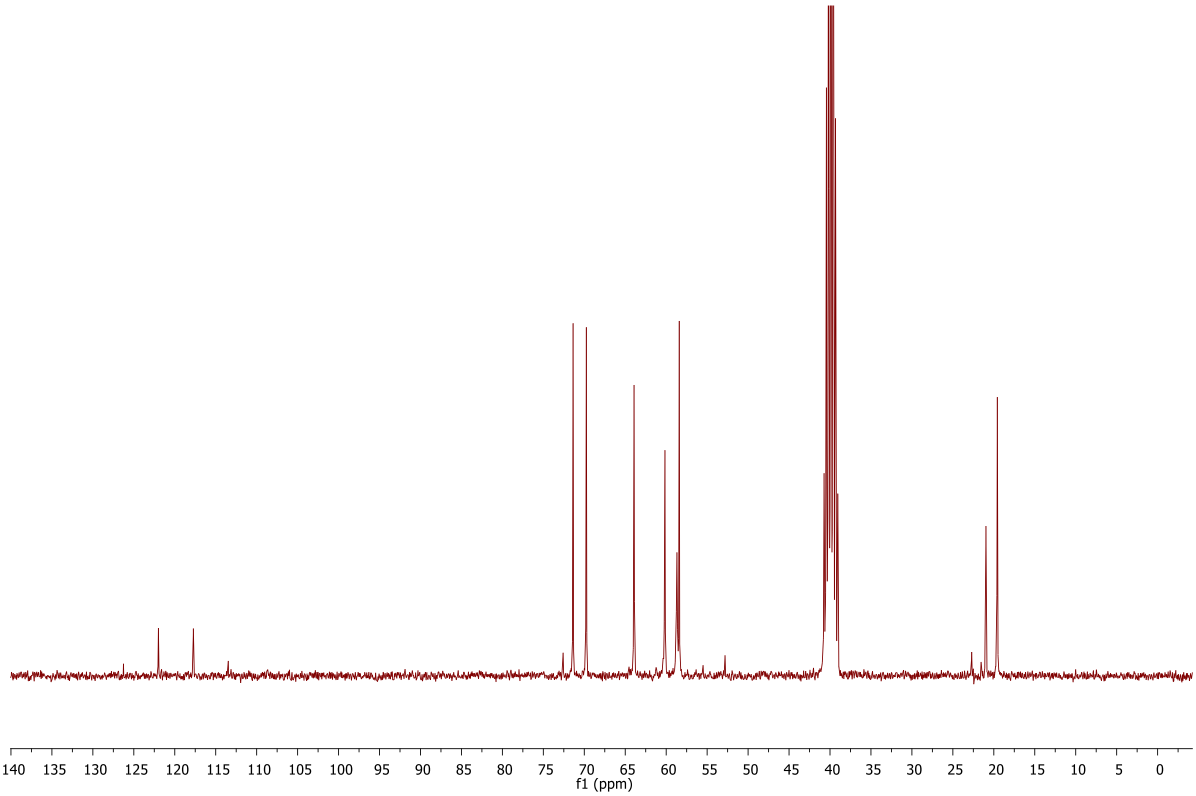

Figure S 34. ^{13}C NMR of $[Pip_{(2o2o1)2}][TFSI]$ in d_6-DMSO.

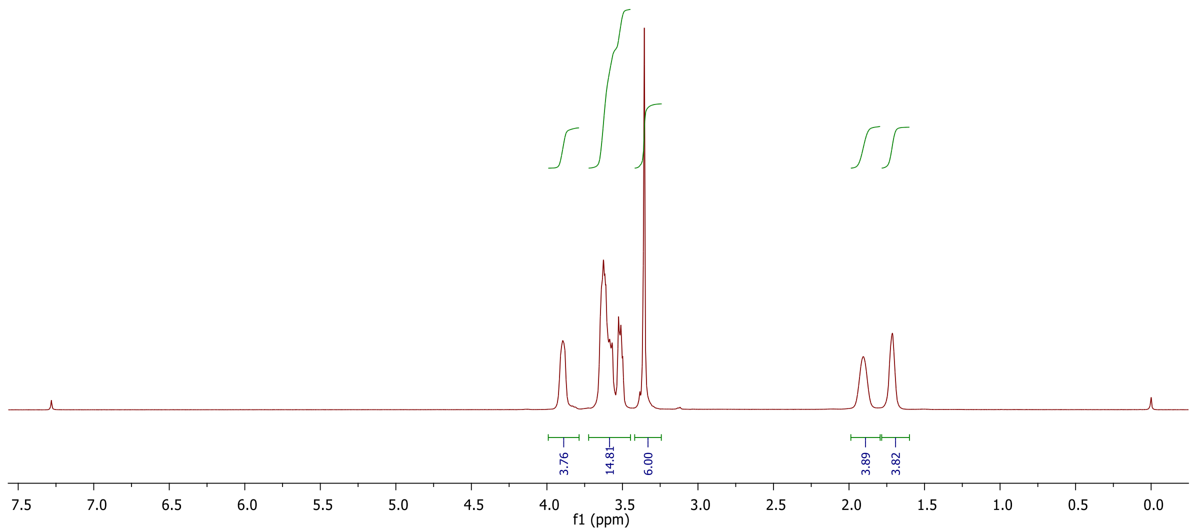

Figure S 35. ^{1}H NMR of [Aze$_{(2o2o1)2}$][TFSI] in $CDCl_3$.

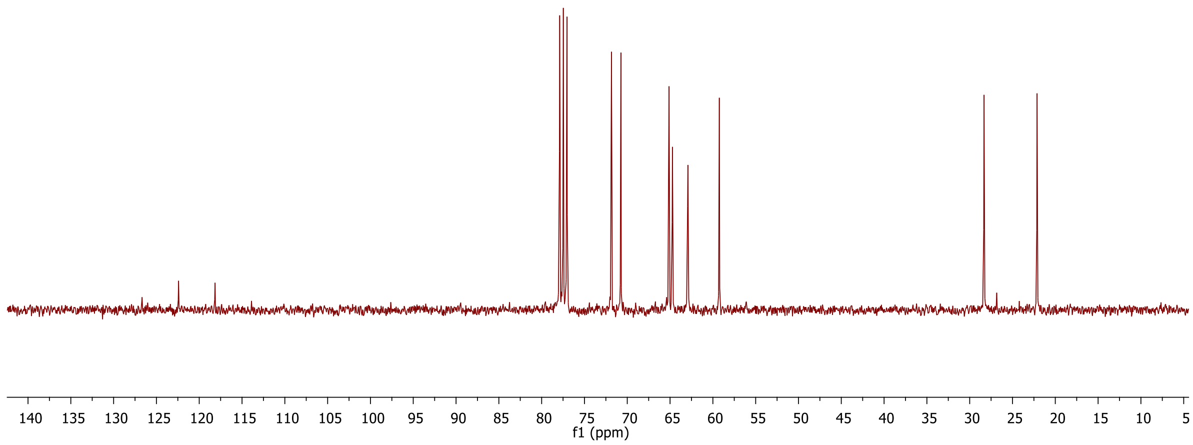

Figure S 36. ^{13}C NMR of [Aze$_{(2o2o1)2}$][TFSI] in $CDCl_3$.
